# Differing perspectives on artificial intelligence in mental healthcare among patients: a cross-sectional survey study

**DOI:** 10.3389/fdgth.2024.1410758

**Published:** 2024-11-29

**Authors:** Meghan Reading Turchioe, Pooja Desai, Sarah Harkins, Jessica Kim, Shiveen Kumar, Yiye Zhang, Rochelle Joly, Jyotishman Pathak, Alison Hermann, Natalie Benda

**Affiliations:** ^1^ Columbia University School of Nursing, New York, NY, United States; ^2^ Department of Biomedical Informatics, Columbia University, New York, NY, United States; ^3^ Department of Population Health Sciences, Weill Cornell Medicine, New York, NY, United States; ^4^ College of Agriculture and Life Sciences, Cornell University, Ithaca, NY, United States; ^5^ Department of Obstetrics and Gynecology, Weill Cornell Medicine, New York, NY, United States; ^6^ Department of Psychiatry, Weill Cornell Medicine, New York, NY, United States

**Keywords:** artificial intelligence, mental health, patient engagement, bioethics aspects, machine learning

## Abstract

**Introduction:**

Artificial intelligence (AI) is being developed for mental healthcare, but patients' perspectives on its use are unknown. This study examined differences in attitudes towards AI being used in mental healthcare by history of mental illness, current mental health status, demographic characteristics, and social determinants of health.

**Methods:**

We conducted a cross-sectional survey of an online sample of 500 adults asking about general perspectives, comfort with AI, specific concerns, explainability and transparency, responsibility and trust, and the importance of relevant bioethical constructs.

**Results:**

Multiple vulnerable subgroups perceive potential harms related to AI being used in mental healthcare, place importance on upholding bioethical constructs, and would blame or reduce trust in multiple parties, including mental healthcare professionals, if harm or conflicting assessments resulted from AI.

**Discussion:**

Future research examining strategies for ethical AI implementation and supporting clinician AI literacy is critical for optimal patient and clinician interactions with AI in mental healthcare.

## Introduction

Artificial intelligence (AI) technologies are increasingly being developed to predict, diagnose, and treat disease. While still in the formative stages of development, AI implementation is increasing in pace across health systems and will soon be widespread. Within mental healthcare, a range of datasets has been used, from electronic health records and imaging data to social media and novel activity and mood monitoring systems, to predict outcomes (1). AI has also been used in a generative manner to summarize information, provide feedback for patient questions, or automate documentation. For example, predictive AI can evaluate the risk of postpartum depression in settings where screening is limited, and generative AI can deliver cognitive-behavioral therapy via chatbot (2, 3). Because rich information is stored in clinical encounter notes so often in mental health contexts, much of this work also leverages natural language processing, a set of computational methods to extract patterns from large sources of textual data (1). In the context of limited mental health resources and mental health professionals, AI technologies provide the opportunity to expand access for patients and offload the tasks clinicians find most burdensome so they may instead focus on the patient relationship that is so unique and essential for psychiatry (4).

Patient perspectives on the use of AI in mental healthcare are critical but missing. As patient engagement and shared decision-making continue to rise in importance, it is likely that patients will interact with AI-generated risk prediction models during the course of their care (5). Moreover, AI-informed chatbots are a potential modality for delivering therapy (3). In addition to gathering general perspectives, it will be important to examine differences in perspectives based on experiences with health and the healthcare system, such as experiences with mental healthcare, implicit bias resulting from one's identity, or social determinants of health (SDOH) that influence access to care and health outcomes.

Moreover, important ethical questions arise when using AI in mental healthcare relating to issues of bias, privacy, autonomy, and distributive justice (6). For example, the broad range of health-related data that may be used to train AI algorithms may alarm patients and undermine trust. For example, mental health researchers are already attempting to correlate keystrokes and voice data captured from smartphones to mood disorders (7), which patients may be unaware of. Moreover, these datasets may have systematic biases that perpetuate inequities in who is able to access mental healthcare or experience positive mental health outcomes (8). AI could also lead to harm when instructions on appropriate reliance are not communicated to patients; for example, researchers have raised concerns about patients developing a perceived therapeutic alliance with chatbots that could cause them to over-rely on chatbot guidance or undermine the therapeutic alliance they have with their mental health professionals (3). A synthesis of the ethics literature on responsible AI (6), consumer-generated data (9), AI in psychiatry (10), and maternal health (11, 12) reveals six important constructs to be considered in this context: autonomy, beneficence/non-maleficence, justice, trust, privacy, and transparency.

However, to date, AI research has focused almost exclusively on presenting model output and fostering trust in AI among clinicians (13). The information needs and ethical considerations among patients likely differ from those of clinicians, but may not be the same for all patients; they may differ by a patient's mental health history, demographic variables, and SDOH (14). Therefore it is important to provide insight to the psychiatry community regarding patients' nuanced perspectives on AI, so that it may be effectively integrated into clinical care in a way that does not degrade the patient relationship. Several prior studies have reported on patients' perceptions of AI in healthcare broadly, which have included a range of concerns and highlighted the importance of understanding and integrating patient views into AI development and deployment (15–17). Within mental healthcare specifically, patient perspectives on AI are comparatively understudied, but early research suggests patients are optimistic. For example, a recent scoping review of patient attitudes towards chatbots in mental healthcare reported that patients have positive perceptions but require high-quality, guideline-concordant, trustworthy and personalized interactions to feel comfortable using them in their care (18). However, differences in attitudes towards AI in mental healthcare by clinical or sociodemographic characteristics remain understudied.

The primary objective of this study was to determine whether a history of mental illness or current mental health status is associated with differences in attitudes towards AI being used in mental healthcare. Secondarily, we also aimed to investigate differences by demographic characteristics (age, gender, race, and ethnicity) and SDOH (financial resources, education, health literacy, and subjective numeracy).

## Methods

### Sample and study design

This study involved a cross-sectional survey administered to an online sample of U.S. adults in September 2022. Participants were recruited using Prolific (19), a web-based recruitment and survey administration platform. Inclusion criteria were: (1) age 18 or older and (2) able to complete the survey in English. Using Prolific's survey sampling strata, we recruited a sample balanced on age, gender, and race reflecting the U.S. demographic distributions (20). The Weill Cornell Medicine (WCM) Institutional Review Board approved this study.

### Data collection

Survey questions addressed six domains relating to attitudes toward AI. Survey items were developed with input from experts in AI, human-centered design, and psychiatry, and some were based on a prior study of general (non-mental health) AI uses in healthcare (21). The topics included general perspectives (baseline knowledge and general attitudes towards AI in mental health), comfort with the use of AI in place of mental health professionals for various tasks and with data sharing for AI purposes, specific concerns regarding the use of AI for mental healthcare, explainability and transparency of how the model works and what data are used, impacts on trust in clinicians and responsibility for harms from AI, and the importance of the six relevant bioethical constructs in this context. Additionally, we collected sociodemographic characteristics, health literacy measured with the Chew Brief Health Literacy Screening Questions (22), subjective numeracy measured with the 3-Item Version of the Subjective Numeracy Scale (23), and mental health history from all participants. Mental health history was assessed with a single item: “Has a trained health professional ever told you that you have any mental illness?” with an explanation of what was considered a trained health professional.

Data were collected using a secure local instance of Qualtrics survey software. Eligible participants were recruited via Prolific and directed to complete the Qualtrics survey. Participants provided informed consent prior to initiating the survey and were compensated at an hourly rate of $13.60 consistent with Prolific policies. At the beginning and throughout the survey, we provided participants with definitions of AI, mental health, clinical depression, and bipolar disorder using lay terms. Additionally, per Prolific recommendations (20), two “attention check” questions were included in the survey to ensure survey respondents were fully reading each question and thoughtfully responding. All survey questions, including the specific attention check questions, are provided in Supplementary File 1.

### Statistical analysis

We first assessed data missingness and the time taken to complete the survey. We planned to exclude participants who did not complete the survey, failed both attention check questions, or completed the survey in under three minutes (suggesting inattention to the questions). We computed basic descriptive statistics of mean, frequency, and central tendency to characterize the sample. We used Fisher's Exact tests to compare differences in survey responses by mental health history, current mental health rating, demographic characteristics, and SDOH. Statistical significance (alpha) was set at 0.05. After these omnibus tests of significant differences were run, visual inspections of percentages in the contingency tables were used to identify differences between groups. We used R version 4.2.1 (R Foundation for Statistical Computing, Vienna, Austria, 2022) for the analysis.

## Results

### Description of the sample

Five hundred participants ultimately completed the survey. Thirty participants opened the survey but did not complete it after reading the consent form, and were therefore excluded from the analysis. No participants failed the attention check questions or completed the survey in under three minutes.

The descriptive characteristics of the sample are described in Table 1. The median age was 46 (interquartile range 31–59). Approximately one-third of the sample was split between Millennials (31%), Generation X (26%), and Baby Boomers (30%). The sample was evenly split by gender, but the majority were White (78%) and non-Hispanic/Latino (93%). One-third reported not having enough financial resources to “make ends meet” (31%) and nearly half had obtained less than a Bachelor's degree (46%). One-quarter had inadequate health literacy (26%) and more than half had low subjective numeracy (57%). Nearly half had a history of mental illness (43%) and nearly one-third rated their current mental health as poor or fair (29%).

**Table 1:** Demographic characteristics of survey participants (*n* = 500); median (interquartile range) or *n* (%).

Age	46 (31, 59)
Age (Generation)	
Generation Z	51 (10%)
Millennial	155 (31%)
Generation X	132 (26%)
Baby Boomer	152 (30%)
Silent Generation	10 (2.0%)
Gender	
Female	249 (50%)
Male	238 (48%)
Transgender or non-binary	10 (2%)
Prefer not to answer	3 (<1%)
Race	
Asian	25 (5.0%)
Black or African American	66 (13%)
White	388 (78%)
Other/Prefer not to answer	21 (4.2%)
Ethnicity	
Hispanic/Latino	30 (6.0%)
Not Hispanic/Latino	464 (93%)
Other/Prefer not to answer	6 (1.2%)
Finances	
More than enough	65 (13%)
Enough	271 (54%)
Not enough	156 (31%)
Prefer not to answer	8 (1.6%)
Education	
Less than Bachelor's Degree	230 (46%)
Bachelor's Degree	189 (38%)
More than Bachelor's Degree	80 (16%)
Prefer not to answer	1 (0.2%)
Health Literacy	
Adequate	369 (74%)
Inadequate	131 (26%)
Subjective Numeracy (Categorized)	
Low Subjective Numeracy	283 (57%)
High Subjective Numeracy	216 (43%)
(Missing)	1
Ever been told have mental illness	
Yes	215 (43%)
No	271 (54%)
Prefer not to answer	14 (2.8%)
Current mental health rating	
Excellent/very good	204 (41%)
Good	148 (30%)
Fair/poor	146 (29%)
Don't know	2 (0.4%)

### Primary endpoints: mental health history and current status

**Figure 1:**
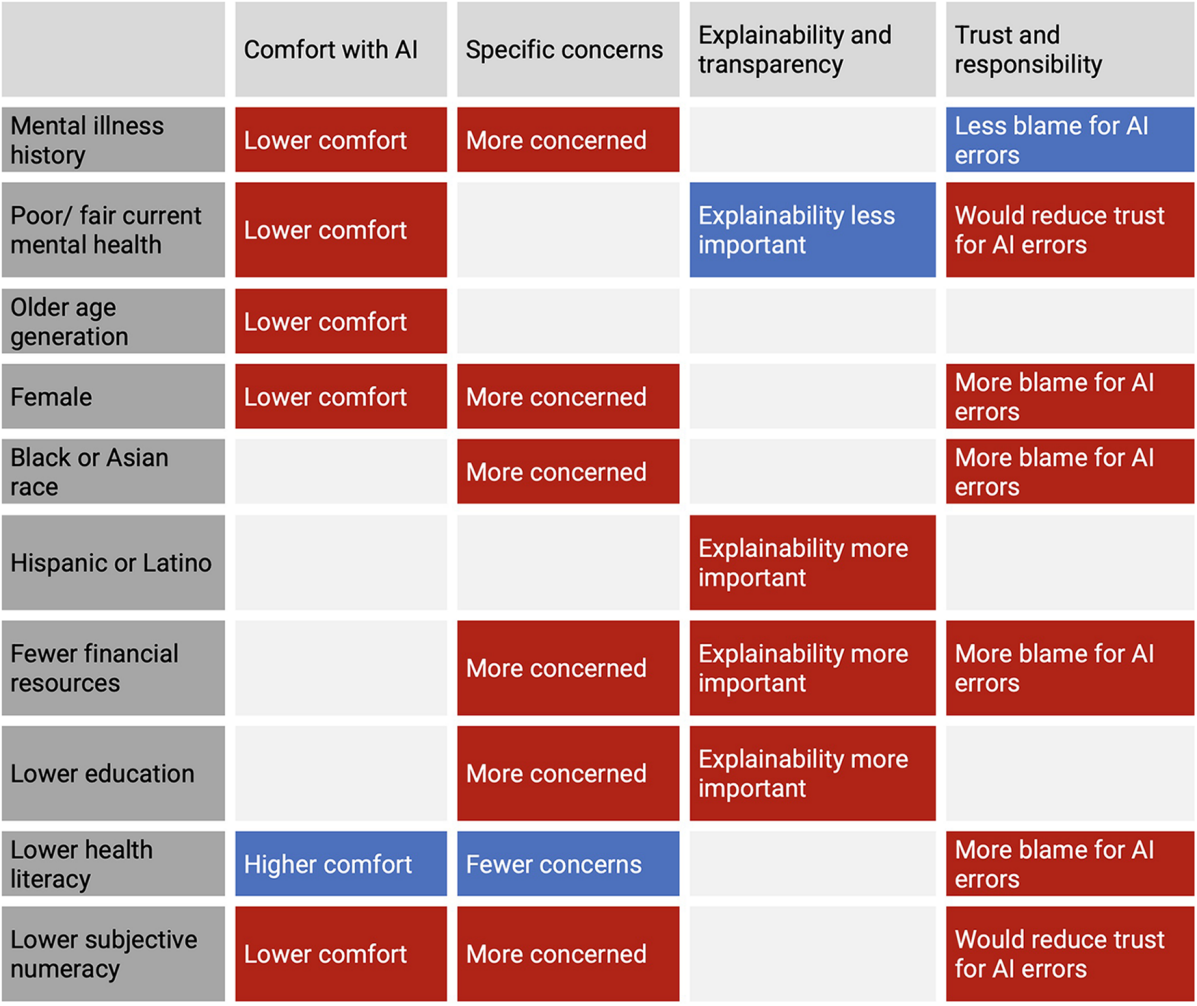
Summary of statistically significant differences in responses across primary (mental health) and secondary (demographic and social determinants of health) endpoints.

A summary of the differences in responses across all primary and secondary endpoints is summarized in Figure 1. All results (including those that were not statistically significant) are reported in Supplementary File 2.

**Figure 2:**
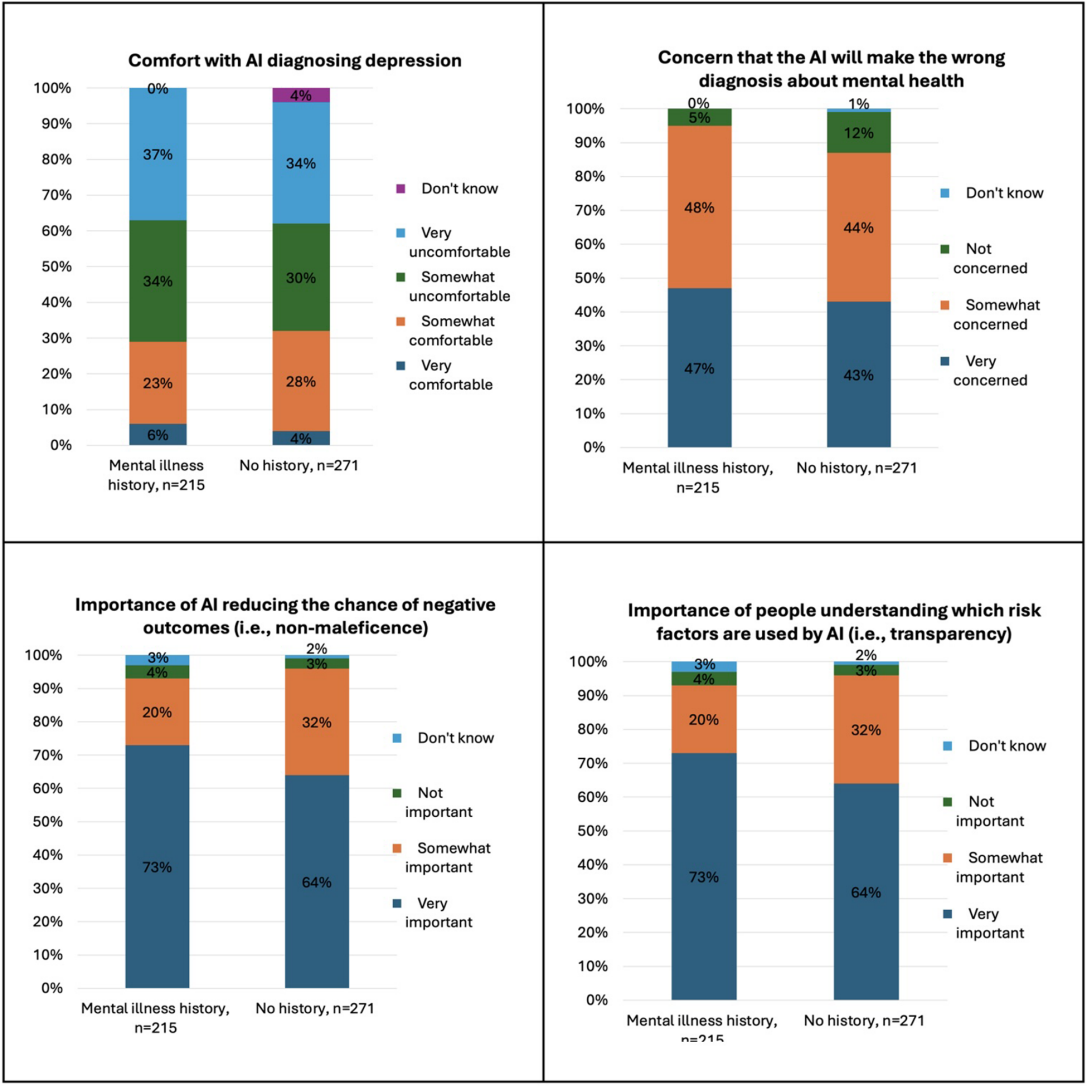
Significant differences in attitudes towards AI in mental healthcare by mental illness history.

**Figure 3:**
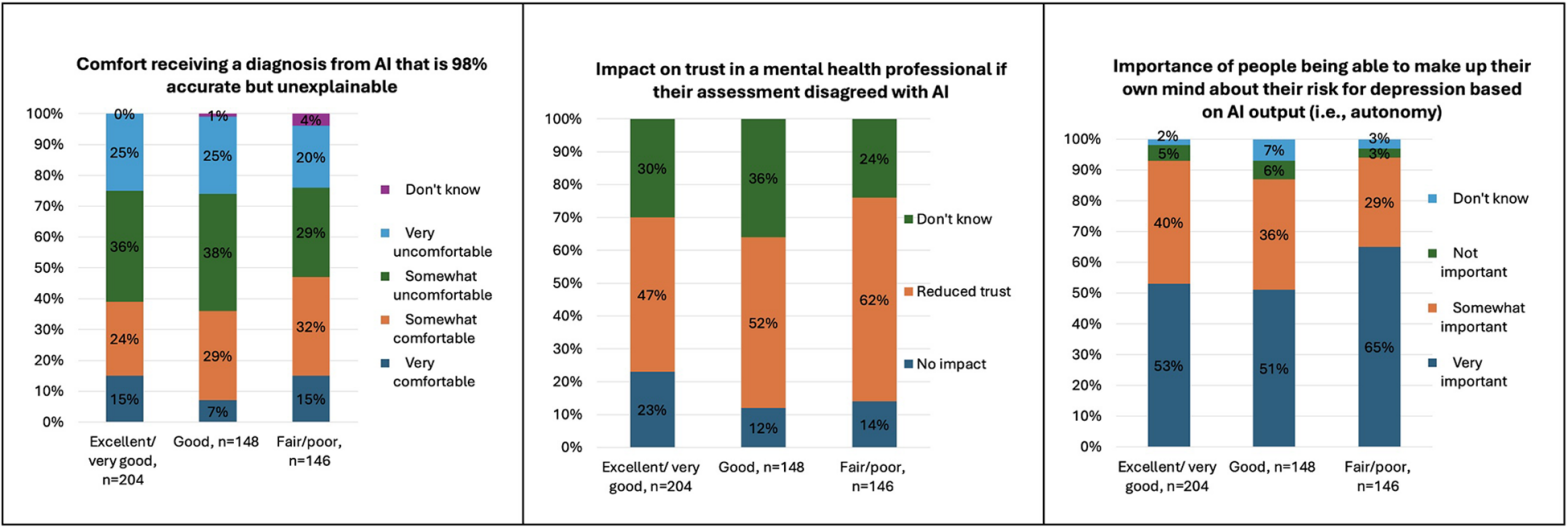
Significant differences in attitudes towards AI in mental healthcare by current self-rated mental health.

Significant differences in perspectives by mental health history and current mental health rating are shown in Figures 2, 3 and Table 2. Compared to those *without* a history of mental illness, more participants *with* a history of mental illness were uncomfortable with AI making a diagnosis of depression (*p* = 0.02) but were more comfortable sharing sensitive information with a mental health professional (*p* = 0.03). More participants with a history of mental illness were also concerned about AI making a wrong diagnosis (*p* = 0.03) and endorsed the importance of non-maleficence (*p* = 0.02) and transparency (*p* = 0.01). More participants with a history of mental illness did not assign responsibility and blame for errors from AI to various groups, including hospitals (*p* < 0.01), companies (*p* = 0.01), and government agencies (*p* = 0.02), and endorsed that “no one” was to blame (*p* = 0.03).

**Table 2:** Significant differences in perspectives on AI in mental health by mental health history and current rating among survey participants (*n* = 500); *n* (%).

	Mental illness history		Current mental health rating		
	Mental illness history, *n* = 215	No history, *n* = 271	Excellent/very good, *n* = 204	Good, *n* = 148	Fair/poor, *n* = 146
General perspectives					
How much do you know about AI and how it could change mental healthcare?					
I know quite a lot	3 (1.4%)	4 (1.5%)	5 (2.5%)	0 (0%)	2 (1.4%)
I know a fair amount	43 (20%)	46 (17%)	41 (20%)	26 (18%)	25 (17%)
I know a little bit	103 (48%)	141 (52%)	88 (43%)	89 (60%)	73 (50%)
I know almost nothing	66 (31%)	80 (30%)	70 (34%)	33 (22%)	46 (32%)
Comfort with AI					
AI, instead of a mental health professional, making a diagnosis of clinical depression					
Very comfortable	13 (6.0%)	13 (4.8%)	12 (5.9%)	6 (4.1%)	8 (5.5%)
Somewhat comfortable	50 (23%)	76 (28%)	50 (25%)	36 (24%)	42 (29%)
Somewhat uncomfortable	73 (34%)	81 (30%)	61 (30%)	47 (32%)	49 (34%)
Very uncomfortable	79 (37%)	91 (34%)	76 (37%)	58 (39%)	43 (29%)
Don't know	0 (0%)	10 (3.7%)	5 (2.5%)	1 (0.7%)	4 (2.7%)
Sharing sensitive information with a human mental health professional					
Very comfortable	90 (42%)	88 (32%)	84 (41%)	49 (33%)	48 (33%)
Somewhat comfortable	80 (37%)	122 (45%)	81 (40%)	70 (47%)	56 (38%)
Somewhat uncomfortable	38 (18%)	40 (15%)	29 (14%)	24 (16%)	28 (19%)
Very uncomfortable	7 (3.3%)	20 (7.4%)	10 (4.9%)	5 (3.4%)	13 (8.9%)
Don't know	0 (0%)	1 (0.4%)	0 (0%)	0 (0%)	1 (0.7%)
Sharing sensitive information to help improve AI programs that treat disease					
Very comfortable	53 (25%)	58 (21%)	54 (26%)	26 (18%)	32 (22%)
Somewhat comfortable	81 (38%)	104 (38%)	67 (33%)	67 (45%)	54 (37%)
Somewhat uncomfortable	48 (22%)	50 (18%)	34 (17%)	35 (24%)	30 (21%)
Very uncomfortable	22 (10%)	47 (17%)	40 (20%)	14 (9.5%)	20 (14%)
Don't know	11 (5.1%)	12 (4.4%)	9 (4.4%)	6 (4.1%)	10 (6.8%)
Specific concerns					
That the AI will make the wrong diagnosis about my mental health					
Very concerned	100 (47%)	116 (43%)	83 (41%)	74 (50%)	62 (42%)
Somewhat concerned	102 (48%)	119 (44%)	93 (46%)	65 (44%)	70 (48%)
Not concerned	12 (5.6%)	32 (12%)	25 (12%)	7 (4.8%)	14 (9.6%)
Don't know	0 (0%)	4 (1.5%)	3 (1.5%)	1 (0.7%)	0 (0%)
(Missing)	1	0	0	1	0
Explainability and transparency					
Comfort receiving a diagnosis from AI that is 98% accurate but unexplainable					
Very comfortable	25 (12%)	37 (14%)	31 (15%)	10 (6.8%)	22 (15%)
Somewhat comfortable	61 (28%)	75 (28%)	49 (24%)	43 (29%)	46 (32%)
Somewhat uncomfortable	74 (34%)	91 (34%)	73 (36%)	56 (38%)	43 (29%)
Very uncomfortable	51 (24%)	63 (23%)	50 (25%)	37 (25%)	29 (20%)
Don't know	4 (1.9%)	5 (1.8%)	1 (0.5%)	2 (1.4%)	6 (4.1%)
Trust and responsibility					
[Given scenario where AI and doctor opinion conflicts] How does the computer program affect your view?					
It would not affect my trust of the mental health professional's assessment	27 (13%)	56 (21%)	47 (23%)	18 (12%)	20 (14%)
It would make me question the mental health professional's assessment	120 (56%)	136 (50%)	96 (47%)	77 (52%)	91 (62%)
I do not know if it would change my view of the mental health professional's assessment	62 (29%)	72 (27%)	57 (28%)	47 (32%)	32 (22%)
Don't know	6 (2.8%)	7 (2.6%)	4 (2.0%)	6 (4.1%)	3 (2.1%)
Responsibility for medical errors resulting from AI (select all that apply)					
Hospital or clinic that bought the computer program	51 (24%)	97 (36%)	62 (30%)	39 (26%)	51 (35%)
Government agency that approved the computer program	39 (18%)	73 (27%)	43 (21%)	31 (21%)	40 (27%)
Blame for medical errors resulting from AI (select all that apply)					
Company that made the computer program	59 (27%)	104 (38%)	69 (34%)	46 (31%)	52 (36%)
Hospital or clinic that bought the computer program	43 (20%)	80 (30%)	53 (26%)	33 (22%)	37 (25%)
No one	13 (6.0%)	5 (1.8%)	6 (2.9%)	9 (6.1%)	4 (2.7%)
Importance of bioethical constructs					
How important in general: That people are able to make up their own mind about their risk for depression based on AI output [autonomy]					
Very important	127 (59%)	147 (54%)	108 (53%)	76 (51%)	95 (65%)
Somewhat important	72 (33%)	100 (37%)	82 (40%)	54 (36%)	42 (29%)
Not important	7 (3.3%)	15 (5.5%)	10 (4.9%)	8 (5.4%)	4 (2.7%)
Don't know	9 (4.2%)	9 (3.3%)	4 (2.0%)	10 (6.8%)	5 (3.4%)
How important in general: That AI will reduce the chance of negative outcomes [non-maleficence]					
Very important	158 (73%)	173 (64%)	124 (61%)	103 (70%)	109 (75%)
Somewhat important	44 (20%)	87 (32%)	67 (33%)	38 (26%)	31 (21%)
Not important	8 (3.7%)	8 (3.0%)	8 (3.9%)	4 (2.7%)	4 (2.7%)
Don't know	5 (2.3%)	3 (1.1%)	5 (2.5%)	3 (2.0%)	2 (1.4%)
How important in general: That people can understand which of their individual risk factors for depression are used by the AI [transparency]					
Very important	184 (86%)	220 (81%)	167 (82%)	125 (84%)	118 (81%)
Somewhat important	22 (10%)	42 (15%)	26 (13%)	21 (14%)	21 (14%)
Not important	2 (0.9%)	8 (3.0%)	6 (2.9%)	1 (0.7%)	3 (2.1%)
Don't Know	7 (3.3%)	1 (0.4%)	5 (2.5%)	1 (0.7%)	4 (2.7%)

Statistical significance represented by grey boxes. Statistical significance determined using Fisher's Exact Test for Count Data with simulated *p*-value (based on 2,000 replicates).

Compared to those who rated their mental health as excellent/very good or good, participants who rated their mental health as poor/fair reported low baseline knowledge about AI and how it could change mental healthcare (*p* = 0.03). More participants who rated their mental health as poor/fair were uncomfortable sharing sensitive information to help improve AI programs that treat disease (*p* = 0.04), would reduce trust in a mental health professional if their assessment disagreed with AI (*p* = 0.02) and more were somewhat comfortable receiving a diagnosis from AI that is 98% accurate but unexplainable (*p* = 0.03).

### Secondary endpoints: demographic differences

Significant differences in perspectives by age generation and gender are shown in Table 3. Compared to all other generations, fewer members of the Baby Boomer generation were comfortable with AI making recommendations for a general wellness or stress-management strategy (*p* < 0.01), talk therapy (*p* = 0.02), or medications (*p* < 0.01). Compared to all other generations, more members of Generation Z and the Millennial generation felt it was “not important” that AI not reduce trust in one's mental health care provider (*p* < 0.01). Compared to men, more women reported low baseline knowledge about AI and how it could change mental healthcare (*p* < 0.01), were uncomfortable with AI diagnosing bipolar disorder (*p* = 0.03), and were “very” concerned that AI will lead to mental health professionals not knowing you as well (*p* = 0.02). Slightly more women were “very” comfortable with AI making recommendations for a general wellness or stress-management strategy (*p* = 0.02) and would assign responsibility and blame for errors from AI to mental health professionals (*p* = 0.03).

**Table 3:** Differences in perspectives on AI in mental health by demographic characteristics (age and gender) among survey participants (*n* = 500); *n* (%).

	Age (generation)					Gender	
	Gen Z, *n* = 51	Millennial, *n* = 155	Gen X, *n* = 132	Boomer, *n* = 152	Silent, *n* = 10	Female, *n* = 249	Male, *n* = 238
General perspectives							
How much do you know about AI and how it could change mental healthcare?							
I know quite a lot	2 (3.9%)	1 (0.6%)	3 (2.3%)	1 (0.7%)	0 (0%)	1 (0.4%)	6 (2.5%)
I know a fair amount	7 (14%)	38 (25%)	26 (20%)	20 (13%)	1 (10%)	28 (11%)	63 (26%)
I know a little bit	31 (61%)	78 (50%)	64 (48%)	74 (49%)	5 (50%)	129 (52%)	114 (48%)
I know almost nothing	11 (22%)	38 (25%)	39 (30%)	57 (38%)	4 (40%)	91 (37%)	55 (23%)
Comfort with AI							
AI, instead of a mental health professional, telling you that you have bi-polar disorder							
Very comfortable	3 (5.9%)	7 (4.5%)	10 (7.6%)	2 (1.3%)	1 (10%)	10 (4.0%)	12 (5.0%)
Somewhat comfortable	8 (16%)	30 (19%)	21 (16%)	18 (12%)	0 (0%)	30 (12%)	42 (18%)
Somewhat uncomfortable	18 (35%)	52 (34%)	37 (28%)	42 (28%)	4 (40%)	75 (30%)	76 (32%)
Very uncomfortable	20 (39%)	64 (41%)	59 (45%)	85 (56%)	5 (50%)	130 (52%)	99 (42%)
Don't know	2 (3.9%)	2 (1.3%)	5 (3.8%)	5 (3.3%)	0 (0%)	4 (1.6%)	9 (3.8%)
AI, instead of a mental health professional, recommending a general wellness or stress-management strategy							
Very comfortable	15 (29%)	39 (25%)	51 (39%)	25 (16%)	4 (40%)	71 (29%)	57 (24%)
Somewhat comfortable	21 (41%)	79 (51%)	48 (36%)	73 (48%)	3 (30%)	109 (44%)	111 (47%)
Somewhat uncomfortable	9 (18%)	18 (12%)	19 (14%)	27 (18%)	2 (20%)	40 (16%)	35 (15%)
Very uncomfortable	2 (3.9%)	17 (11%)	13 (9.8%)	24 (16%)	1 (10%)	28 (11%)	28 (12%)
Don't know	4 (7.8%)	2 (1.3%)	1 (0.8%)	3 (2.0%)	0 (0%)	1 (0.4%)	7 (2.9%)
AI, instead of a mental health professional, recommending a talk therapy							
Very comfortable	10 (20%)	38 (25%)	40 (30%)	24 (16%)	3 (30%)	57 (23%)	52 (22%)
Somewhat comfortable	22 (43%)	73 (47%)	55 (42%)	58 (38%)	4 (40%)	103 (41%)	106 (45%)
Somewhat uncomfortable	12 (24%)	19 (12%)	21 (16%)	35 (23%)	1 (10%)	41 (16%)	45 (19%)
Very uncomfortable	4 (7.8%)	21 (14%)	15 (11%)	33 (22%)	2 (20%)	41 (16%)	32 (13%)
Don't know	3 (5.9%)	4 (2.6%)	1 (0.8%)	2 (1.3%)	0 (0%)	7 (2.8%)	3 (1.3%)
AI, instead of a mental health professional, recommending a medication							
Very comfortable	5 (9.8%)	8 (5.2%)	15 (11%)	4 (2.6%)	1 (10%)	12 (4.8%)	20 (8.4%)
Somewhat comfortable	15 (29%)	37 (24%)	22 (17%)	27 (18%)	2 (20%)	47 (19%)	51 (21%)
Somewhat uncomfortable	15 (29%)	57 (37%)	38 (29%)	37 (24%)	2 (20%)	75 (30%)	71 (30%)
Very uncomfortable	14 (27%)	51 (33%)	55 (42%)	82 (54%)	5 (50%)	113 (45%)	90 (38%)
Don't know	2 (3.9%)	2 (1.3%)	2 (1.5%)	2 (1.3%)	0 (0%)	2 (0.8%)	6 (2.5%)
Specific concerns							
That AI will lead to my mental health provider not knowing me as well							
Very concerned	23 (46%)	59 (38%)	54 (41%)	69 (45%)	4 (40%)	124 (50%)	82 (35%)
Somewhat concerned	18 (36%)	69 (45%)	51 (39%)	60 (39%)	2 (20%)	87 (35%)	107 (45%)
Not concerned	8 (16%)	24 (15%)	23 (17%)	21 (14%)	4 (40%)	34 (14%)	42 (18%)
Don't know	1 (2.0%)	3 (1.9%)	4 (3.0%)	2 (1.3%)	0 (0%)	4 (1.6%)	6 (2.5%)
(Missing)	1	0	0	0	0	0	1
**Trust and responsibility**							
Responsibility for medical errors resulting from AI (select all that apply)							
Mental health professional	42 (82%)	118 (76%)	111 (84%)	132 (87%)	9 (90%)	209 (84%)	196 (82%)
Blame for medical errors resulting from AI (select all that apply)							
Mental health professional	40 (78%)	124 (80%)	111 (84%)	126 (83%)	7 (70%)	209 (84%)	192 (81%)
Someone else	1 (2.0%)	2 (1.3%)	4 (3.0%)	3 (2.0%)	0 (0%)	4 (1.6%)	4 (1.7%)
Importance of bioethical constructs							
How important in general: That people can understand which of their individual risk factors for depression are used by the AI [transparency]							
Very important	42 (82%)	118 (76%)	113 (86%)	130 (86%)	9 (90%)	220 (88%)	182 (76%)
Somewhat important	9 (18%)	28 (18%)	15 (11%)	16 (11%)	0 (0%)	21 (8.4%)	45 (19%)
Not important	0 (0%)	3 (1.9%)	4 (3.0%)	2 (1.3%)	1 (10%)	3 (1.2%)	7 (2.9%)
Don't know	0 (0%)	6 (3.9%)	0 (0%)	4 (2.6%)	0 (0%)	5 (2.0%)	4 (1.7%)
How important to you: That using AI does not reduce your trust in your mental health care provider [trust]							
Very important	12 (24%)	60 (39%)	53 (40%)	53 (35%)	8 (80%)	89 (36%)	91 (38%)
Somewhat important	25 (49%)	60 (39%)	59 (45%)	71 (47%)	0 (0%)	113 (45%)	98 (41%)
Not important	10 (20%)	26 (17%)	14 (11%)	15 (9.9%)	0 (0%)	27 (11%)	36 (15%)
Don't know	4 (7.8%)	9 (5.8%)	6 (4.5%)	13 (8.6%)	2 (20%)	20 (8.0%)	13 (5.5%)

Statistical significance represented by grey boxes. Statistical significance determined using Fisher's Exact Test for Count Data with simulated *p*-value (based on 2,000 replicates).

Significant differences in perspectives by race and ethnicity are shown in Table 4. Compared to white participants, more Asian and Black participants thought AI would make mental healthcare “much” or “somewhat” better (*p* = 0.03), but more were also “very” concerned that AI would make a misdiagnosis (*p* = 0.04) and result in less time with their mental health professional (*p* = 0.02), would blame hospitals or clinics (*p* = 0.01) and regulatory governmental agencies for AI errors (*p* = 0.04). Compared to other racial groups, more Asian participants expect mental health professionals to check for AI safety (*p* = 0.02). Compared to non-Hispanic/Latino participants, more Hispanic or Latino participants were both “very comfortable” and “very uncomfortable” (but not “somewhat”) receiving a mental health diagnosis from AI that was 98% accurate but unexplainable.

**Table 4:** Differences in perspectives on AI in mental health by demographic characteristics (race and ethnicity) among survey participants (*n* = 500); *n* (%).

	Race				Ethnicity	
	Asian, *n* = 25	Black or African American, *n* = 66	White, *n* = 388	Other/prefer not to answer, *n* = 211	Hispanic/Latino, *n* = 30	Not Hispanic/Latino, *n* = 464
General perspectives						
Overall, in the next 5 years, do you think AI will make mental healthcare in the United States?						
Much better	0 (0%)	8 (12%)	25 (6.4%)	1 (4.8%)	2 (6.7%)	32 (6.9%)
Somewhat better	15 (60%)	32 (48%)	159 (41%)	7 (33%)	12 (40%)	200 (43%)
Minimal change	6 (24%)	19 (29%)	137 (35%)	8 (38%)	9 (30%)	158 (34%)
Somewhat worse	1 (4.0%)	2 (3.0%)	27 (7.0%)	4 (19%)	2 (6.7%)	31 (6.7%)
Much worse	0 (0%)	3 (4.5%)	4 (1.0%)	1 (4.8%)	2 (6.7%)	5 (1.1%)
Don't know	3 (12%)	2 (3.0%)	36 (9.3%)	0 (0%)	3 (10%)	38 (8.2%)
Specific concerns						
That the AI will make the wrong diagnosis about my mental health						
Very concerned	16 (64%)	34 (52%)	163 (42%)	8 (38%)	15 (50%)	203 (44%)
Somewhat concerned	7 (28%)	24 (36%)	187 (48%)	10 (48%)	14 (47%)	212 (46%)
Not concerned	1 (4.0%)	7 (11%)	36 (9.3%)	2 (9.5%)	1 (3.3%)	44 (9.5%)
Don't know	1 (4.0%)	1 (1.5%)	1 (0.3%)	1 (4.8%)	0 (0%)	4 (0.9%)
(Missing)	0	0	1	0	0	1
That AI will mean I spend less time with my mental health professional						
Very concerned	9 (36%)	22 (33%)	141 (36%)	4 (19%)	14 (47%)	161 (35%)
Somewhat concerned	10 (40%)	23 (35%)	131 (34%)	6 (29%)	8 (27%)	159 (34%)
Not concerned	5 (20%)	13 (20%)	106 (27%)	8 (38%)	8 (27%)	122 (26%)
Don't know	1 (4.0%)	8 (12%)	10 (2.6%)	3 (14%)	0 (0%)	22 (4.7%)
Explainability and transparency						
Comfort receiving a diagnosis from AI that is 98% accurate but unexplainable						
Very comfortable	5 (20%)	8 (12%)	47 (12%)	3 (14%)	6 (20%)	57 (12%)
Somewhat comfortable	7 (28%)	18 (27%)	109 (28%)	4 (19%)	5 (17%)	132 (28%)
Somewhat uncomfortable	7 (28%)	21 (32%)	139 (36%)	5 (24%)	8 (27%)	162 (35%)
Very uncomfortable	6 (24%)	19 (29%)	84 (22%)	8 (38%)	8 (27%)	106 (23%)
Don't know	0 (0%)	0 (0%)	9 (2.3%)	1 (4.8%)	3 (10%)	7 (1.5%)
Trust and responsibility						
Blame for medical errors resulting from AI (select all that apply)						
Hospital or clinic that bought the computer program	12 (48%)	21 (32%)	85 (22%)	7 (33%)	9 (30%)	114 (25%)
Government agency that approved the computer program	9 (36%)	19 (29%)	71 (18%)	6 (29%)	5 (17%)	99 (21%)
Responsibility for checking that AI is safe (select all that apply)						
Mental health professional	14 (56%)	17 (26%)	99 (26%)	5 (24%)	8 (27%)	126 (27%)
Someone else	3 (12%)	0 (0%)	4 (1.0%)	0 (0%)	0 (0%)	7 (1.5%)
Importance of bioethical constructs						
How important to you: That AI will improve your depression/depressive symptoms [beneficence]						
Very important	15 (60%)	38 (58%)	241 (62%)	8 (38%)	23 (77%)	278 (60%)
Somewhat important	4 (16%)	23 (35%)	110 (28%)	7 (33%)	6 (20%)	136 (29%)
Not important	5 (20%)	4 (6.1%)	21 (5.4%)	3 (14%)	1 (3.3%)	31 (6.7%)
Don't know	1 (4.0%)	1 (1.5%)	16 (4.1%)	3 (14%)	0 (0%)	19 (4.1%)

Statistical significance represented by grey boxes. Statistical significance determined using Fisher's Exact Test for Count Data with simulated *p*-value (based on 2,000 replicates).

### Secondary endpoints: social determinants of health

Significant differences in perspectives by financial resources and education are shown in Table 5. Compared to those with “more than enough” financial resources, more participants with “enough” and “not enough” were “very” concerned about AI misdiagnosis (*p* = 0.04) and mental health costs (*p* < 0.01), and were “somewhat” or “very” uncomfortable receiving a mental health diagnosis from both a 90% accurate but unexplainable AI (*p* = 0.01) and a 98% accurate but unexplainable AI (*p* = 0.04). More participants with “enough” and “not enough” also placed greater importance on AI being explainable (*p* = 0.04) and reducing negative outcomes (*p* < 0.01). Compared to those with a Bachelor's or higher, more participants with less than a Bachelor's degree were “very” concerned that AI will increase mental health costs (*p* < 0.01), “very” uncomfortable receiving a mental health diagnosis from AI that was 98% accurate but unexplainable (*p* = 0.03), and “did not know” who was responsible for harm caused by AI (*p* = 0.02).

**Table 5:** Differences in perspectives on AI in mental health by social determinants of health (SDOH; financial resources and education) among survey participants (*n* = 500); *n* (%).

	Financial resources			Education		
	More than enough, *n* = 65	Enough, *n* = 271	Not enough, *n* = 156	More than bachelor's degree, *n* = 80	Bachelor's degree, *n* = 189	Less than bachelor's degree, *n* = 230
Specific concerns						
That the AI will make the wrong diagnosis about my mental health						
Very concerned	20 (31%)	121 (45%)	73 (47%)	32 (40%)	85 (45%)	103 (45%)
Somewhat concerned	34 (52%)	129 (48%)	64 (41%)	39 (49%)	86 (46%)	103 (45%)
Not concerned	10 (15%)	18 (6.6%)	18 (12%)	8 (10%)	15 (8.0%)	23 (10%)
Don't know	1 (1.5%)	3 (1.1%)	0 (0%)	1 (1.3%)	2 (1.1%)	1 (0.4%)
(Missing)	0	0	1	0	1	0
That AI will increase my mental health care costs						
Very concerned	3 (4.6%)	40 (15%)	41 (26%)	4 (5.0%)	29 (15%)	52 (23%)
Somewhat concerned	12 (18%)	70 (26%)	44 (28%)	17 (21%)	43 (23%)	70 (31%)
Not concerned	44 (68%)	134 (50%)	59 (38%)	51 (64%)	100 (53%)	87 (38%)
Don't know	6 (9.2%)	26 (9.6%)	12 (7.7%)	8 (10%)	17 (9.0%)	20 (8.7%)
(Missing)	0	1	0	0	0	1
Explainability and transparency						
Comfort receiving a diagnosis from AI that is 90% accurate but unexplainable						
Very comfortable	3 (4.6%)	6 (2.2%)	6 (3.8%)	4 (5.0%)	5 (2.6%)	6 (2.6%)
Somewhat comfortable	19 (29%)	45 (17%)	28 (18%)	11 (14%)	45 (24%)	38 (17%)
Somewhat uncomfortable	18 (28%)	112 (41%)	51 (33%)	27 (34%)	63 (33%)	94 (41%)
Very uncomfortable	25 (38%)	107 (39%)	64 (41%)	38 (48%)	75 (40%)	85 (37%)
Don't know	0 (0%)	1 (0.4%)	7 (4.5%)	0 (0%)	1 (0.5%)	7 (3.0%)
Comfort receiving a diagnosis from AI that is 98% accurate but unexplainable						
Very comfortable	14 (22%)	28 (10%)	21 (13%)	5 (6.2%)	23 (12%)	35 (15%)
Somewhat comfortable	25 (38%)	71 (26%)	40 (26%)	25 (31%)	63 (33%)	50 (22%)
Somewhat uncomfortable	17 (26%)	103 (38%)	50 (32%)	32 (40%)	55 (29%)	85 (37%)
Very uncomfortable	9 (14%)	65 (24%)	40 (26%)	18 (22%)	46 (24%)	52 (23%)
Don't know	0 (0%)	4 (1.5%)	5 (3.2%)	0 (0%)	2 (1.1%)	8 (3.5%)
Trust and responsibility						
Responsibility for medical errors resulting from AI (select all that apply)						
Don't know	2 (3.1%)	16 (5.9%)	9 (5.8%)	0 (0%)	10 (5.3%)	17 (7.4%)
Blame for medical errors resulting from AI (select all that apply)						
Someone else	2 (3.1%)	1 (0.4%)	6 (3.8%)	3 (3.8%)	3 (1.6%)	4 (1.7%)
Importance of bioethical constructs						
How important in general: That people are able to make up their own mind about their risk for depression based on AI output [autonomy]						
Very important	30 (46%)	144 (53%)	101 (65%)	40 (50%)	100 (53%)	139 (60%)
Somewhat important	32 (49%)	101 (37%)	43 (28%)	35 (44%)	70 (37%)	73 (32%)
Not important	1 (1.5%)	16 (5.9%)	5 (3.2%)	2 (2.5%)	10 (5.3%)	10 (4.3%)
Don't know	2 (3.1%)	10 (3.7%)	7 (4.5%)	3 (3.8%)	9 (4.8%)	8 (3.5%)
How important in general: That AI will reduce the chance of negative outcomes [non-maleficence]						
Very important	40 (62%)	172 (63%)	120 (77%)	51 (64%)	122 (65%)	163 (71%)
Somewhat important	20 (31%)	88 (32%)	26 (17%)	24 (30%)	57 (30%)	56 (24%)
Not important	4 (6.2%)	7 (2.6%)	5 (3.2%)	4 (5.0%)	5 (2.6%)	7 (3.0%)
Don't know	1 (1.5%)	4 (1.5%)	5 (3.2%)	1 (1.3%)	5 (2.6%)	4 (1.7%)

Statistical significance represented by grey boxes. Statistical significance determined using Fisher's Exact Test for Count Data with simulated *p*-value (based on 2,000 replicates).

Significant differences in perspectives by health literacy and subjective numeracy are shown in Table 6. Compared to those with adequate health literacy, more participants with inadequate health literacy thought that AI would make mental healthcare “much” or “somewhat” better (*p* = 0.01), were not concerned about AI leading to spending less time with a mental health professional (*p* < 0.01), were “somewhat” comfortable sharing sensitive information with an AI chatbot (*p* = 0.01) and to help improve AI that treats mental health conditions (*p* < 0.01), and endorsed that privacy was “not important” both in general (*p* < 0.01) and to them personally (*p* = 0.02). However, more participants with inadequate health literacy also felt hospitals or clinics are to blame for harm caused by AI (*p* = 0.02), and had mixed responses regarding their comfort in receiving a diagnosis from AI that is 90% accurate but unexplainable (more endorsed either “somewhat” comfortable or uncomfortable vs. “very”; *p* = 0.03).

**Table 6:** Differences in perspectives on AI in mental health by social determinants of health (SDOH; health literacy and subjective numeracy) among survey participants (*n* = 500); *n* (%).

	Health Literacy		Subjective numeracy	
	Adequate, *n* = 369	Inadequate, *n* = 131	Higher (>14), *n* = 216	Lower (<=14), *n* = 283
General perspectives				
Overall, in the next 5 years, do you think AI will make mental healthcare in the United States?				
Much better	20 (5.4%)	14 (11%)	16 (7.4%)	18 (6.4%)
Somewhat better	145 (39%)	68 (52%)	94 (44%)	119 (42%)
Minimal change	134 (36%)	36 (27%)	72 (33%)	98 (35%)
Somewhat worse	29 (7.9%)	5 (3.8%)	17 (7.9%)	17 (6.0%)
Much worse	7 (1.9%)	1 (0.8%)	2 (0.9%)	6 (2.1%)
Don't know	34 (9.2%)	7 (5.3%)	15 (6.9%)	25 (8.8%)
Comfort with AI				
AI, instead of a mental health professional, telling you that you are clinically depressed				
Very comfortable	20 (5.4%)	9 (6.9%)	19 (8.8%)	10 (3.5%)
Somewhat comfortable	79 (21%)	40 (31%)	44 (20%)	75 (27%)
Somewhat uncomfortable	106 (29%)	41 (31%)	76 (35%)	70 (25%)
Very uncomfortable	154 (42%)	39 (30%)	73 (34%)	120 (42%)
Don't know	10 (2.7%)	2 (1.5%)	4 (1.9%)	8 (2.8%)
AI, instead of a mental health professional, predicting a patient's risk for suicide				
Very comfortable	29 (7.9%)	10 (7.6%)	29 (13%)	10 (3.5%)
Somewhat comfortable	69 (19%)	31 (24%)	38 (18%)	62 (22%)
Somewhat uncomfortable	101 (27%)	36 (27%)	58 (27%)	78 (28%)
Very uncomfortable	149 (40%)	48 (37%)	84 (39%)	113 (40%)
Don't know	21 (5.7%)	6 (4.6%)	7 (3.2%)	20 (7.1%)
AI, instead of a mental health professional, predicting a patient's risk of engaging in violent behavior				
Very comfortable	31 (8.4%)	7 (5.3%)	25 (12%)	13 (4.6%)
Somewhat comfortable	74 (20%)	38 (29%)	42 (19%)	70 (25%)
Somewhat uncomfortable	91 (25%)	36 (27%)	58 (27%)	68 (24%)
Very uncomfortable	153 (41%)	43 (33%)	81 (38%)	115 (41%)
Don't know	20 (5.4%)	7 (5.3%)	10 (4.6%)	17 (6.0%)
Sharing sensitive information with an AI chatbot				
Very comfortable	54 (15%)	18 (14%)	28 (13%)	44 (16%)
Somewhat comfortable	119 (32%)	46 (35%)	74 (34%)	91 (32%)
Somewhat uncomfortable	89 (24%)	41 (31%)	51 (24%)	78 (28%)
Very uncomfortable	104 (28%)	21 (16%)	60 (28%)	65 (23%)
Don't know	3 (0.8%)	5 (3.8%)	3 (1.4%)	5 (1.8%)
Sharing sensitive information to help improve AI programs that treat disease				
Very comfortable	83 (22%)	29 (22%)	53 (25%)	59 (21%)
Somewhat comfortable	132 (36%)	56 (43%)	76 (35%)	112 (40%)
Somewhat uncomfortable	68 (18%)	32 (24%)	43 (20%)	56 (20%)
Very uncomfortable	66 (18%)	8 (6.1%)	37 (17%)	37 (13%)
Don't know	20 (5.4%)	6 (4.6%)	7 (3.2%)	19 (6.7%)
Specific concerns				
That AI will mean I spend less time with my mental health professional				
Very concerned	147 (40%)	29 (22%)	84 (39%)	92 (33%)
Somewhat concerned	116 (31%)	54 (41%)	73 (34%)	96 (34%)
Not concerned	90 (24%)	42 (32%)	51 (24%)	81 (29%)
Don't know	16 (4.3%)	6 (4.6%)	8 (3.7%)	14 (4.9%)
That AI will increase my mental health care costs				
Very concerned	56 (15%)	29 (22%)	35 (16%)	49 (17%)
Somewhat concerned	93 (25%)	38 (29%)	42 (20%)	89 (31%)
Not concerned	184 (50%)	54 (41%)	112 (52%)	126 (45%)
Don't know	35 (9.5%)	10 (7.6%)	26 (12%)	19 (6.7%)
(Missing)	1	0	1	0
Explainability and transparency				
Comfort receiving a diagnosis from AI that is 90% accurate but unexplainable				
Very comfortable	12 (3.3%)	3 (2.3%)	6 (2.8%)	9 (3.2%)
Somewhat comfortable	64 (17%)	30 (23%)	50 (23%)	44 (16%)
Somewhat uncomfortable	127 (34%)	57 (44%)	77 (36%)	106 (37%)
Very uncomfortable	161 (44%)	38 (29%)	81 (38%)	118 (42%)
Don't know	5 (1.4%)	3 (2.3%)	2 (0.9%)	6 (2.1%)
Trust and responsibility				
[Given scenario where AI and doctor opinion conflicts] How does the computer program affect your view?				
It would not affect my trust of the mental health professional's assessment	66 (18%)	19 (15%)	47 (22%)	38 (13%)
It would make me question the mental health professional's assessment	187 (51%)	78 (60%)	102 (47%)	162 (57%)
I do not know if it would change my view of the mental health professional's assessment	107 (29%)	30 (23%)	60 (28%)	77 (27%)
Don't know	9 (2.4%)	4 (3.1%)	7 (3.2%)	6 (2.1%)
Responsibility for medical errors resulting from AI (select all that apply)				
Company that made the computer program	136 (37%)	47 (36%)	91 (42%)	92 (33%)
Blame for medical errors resulting from AI (select all that apply)				
Hospital or clinic that bought the computer program	82 (22%)	43 (33%)	55 (25%)	69 (24%)
Importance of bioethical constructs				
How important in general: That people are aware of how their personal data is being used for AI [privacy]				
Very important	276 (75%)	86 (66%)	160 (74%)	202 (71%)
Somewhat important	66 (18%)	26 (20%)	36 (17%)	55 (19%)
Not important	14 (3.8%)	16 (12%)	14 (6.5%)	16 (5.7%)
Don't know	13 (3.5%)	3 (2.3%)	6 (2.8%)	10 (3.5%)
How important to you: That you are aware of how your personal data is being used for AI [privacy]				
Very important	265 (72%)	83 (63%)	155 (72%)	192 (68%)
Somewhat important	79 (21%)	28 (21%)	45 (21%)	62 (22%)
Not important	17 (4.6%)	17 (13%)	13 (6.0%)	21 (7.4%)
Don't know	8 (2.2%)	3 (2.3%)	3 (1.4%)	8 (2.8%)

Statistical significance represented by grey boxes. Statistical significance determined using Fisher's Exact Test for Count Data with simulated *p*-value (based on 2,000 replicates).

Compared to those with high subjective numeracy, more participants with low subjective numeracy were “very” uncomfortable with AI diagnosing depression (*p* < 0.01), predicting the risk of suicide (*p* < 0.01), and predicting the risk of engaging in violent behavior (*p* = 0.03). More participants with low subjective numeracy were also “very” or “somewhat” concerned that AI will increase mental health costs (*p* < 0.01), and would reduce trust in a mental health professional if their assessment disagreed with AI (*p* = 0.04), but fewer would blame the company making the AI for harm caused by AI (*p* = 0.03).

## Discussion

In this analysis of 500 U.S. adults, significant differences in perceptions of AI in mental healthcare were found by mental health history, current mental health status, demographic characteristics, and SDOH. In general, participants with a history of mental illness, members of older generations (e.g., Baby Boomers), those identifying as women, Asian, Black, Latinx, those reporting “not enough” financial resources, those with less than a Bachelor's degree in education, and those with lower subjective numeracy expressed greater discomfort with various potential uses of AI in mental healthcare and expressed greater concerns about potential harms from AI in mental healthcare. Similarly, more participants with a history of mental illness, who rated their current mental health as poor or fair, members of older generations (e.g., Baby Boomers), those who identified as women, and those reporting “not enough” financial resources felt at least one of the bioethical constructs were “very important.” Paradoxically, participants with inadequate health literacy were more comfortable with AI, less concerned about its potential harms, and rated some of the bioethical constructs as “less important.”

This study is among the first to describe differences in attitudes towards AI by mental health history; to our knowledge, this has not been previously reported. However, our findings do align with prior research on patient perspectives on AI in healthcare more broadly, in which individuals reporting good to excellent health are less concerned and more comfortable with AI in healthcare compared to those with poorer health status (24). Our findings on differences in attitudes by other characteristics generally align with the small but growing body of literature in the area. Specifically, in prior studies, women, older and racially and ethnically minoritized adults, and those with lower education, socioeconomic status, and/or digital literacy, express greater concern about the use of AI in mental healthcare, particularly surrounding privacy, accuracy, lack of transparency, loss of human empathy and connection, and financial costs (24, 25).

These findings highlight significant concerns among multiple vulnerable populations about the use of AI in mental healthcare. At the same time, they are unsurprising in the context of historical failures to consider and integrate the needs of vulnerable populations when emerging technologies are developed and integrated into healthcare systems (26). For example, there are known and persistent disparities in the adoption and use of patient portals (27), telehealth (28), and wearable technologies (29, 30) by many of the groups of patients who expressed concerns and discomfort with AI in this study. In fact, the finding that lower health literacy participants did not perceive these threats suggests that these concerns could be realized, as the patients least able to find, understand, and use health-related information are the least aware or concerned about AI-related harms. Future research should examine the intersectionality of multiple vulnerabilities and its impact on perceptions of AI in mental healthcare, given that important differences along a matrix of personal characteristics may exist that were not uncovered in this analysis.

In this study, participants’ perceptions of who is responsible or to blame for harm resulting from AI, who is responsible for ensuring the safety of AI, and the impacts on trust with mental health professionals were inconsistent across vulnerable groups. In general, more participants who identified as Asian or Black, reported “not enough” financial resources, or had inadequate health literacy assigned responsibility or blame to hospitals or clinics, regulatory governmental agencies, mental health professionals, and/or other groups for harm resulting from AI. More participants who rated their current mental health as poor or fair, and those with lower subjective numeracy, would also reduce trust in their mental health professional if their assessment disagreed with AI. However, more participants with lower education were unsure about who was responsible, fewer participants with a history of mental illness would assign responsibility or blame to hospitals or clinics, regulatory governmental agencies, and AI companies, and fewer participants with lower subjective numeracy would blame AI companies for harms caused by AI.

Given the speed with which AI is being embraced across many disciplines within healthcare, including mental healthcare, and the perspective among some groups of patients that mental health professionals are responsible, hold blame, or are less trustworthy as a result of harm or conflicting information from AI, *AI literacy* among clinicians is imperative. AI literacy calls for clinicians to understand data governance, basic statistics, data visualization, and the impact of AI on clinical processes (31). Mental health professionals will need to be able to continue practicing in an effective, efficient, safe, and equitable way in the context of AI-enriched healthcare systems. Perhaps more importantly, it will be essential that AI will not be used, inadvertently or otherwise, to extend existing biases that create disparities between patients in timely and accurate mental health diagnoses and care. Their perspectives will also be critical as those developing AI systems for mental healthcare aim to create more accurate systems, for example using deep learning methods, while combating the “black box” problem of limited explainability (1, 3). Similarly, reviews have demonstrated that AI in mental healthcare is in an early, proof-of-concept stage; in future work, the engagement of mental health professionals, along with patients and caregivers, will be critical to successful clinical implementation in ways that do not overburden clinicians (1).

AI-related competencies for clinicians have been proposed; Russell et al. (32) identified six competencies including basic knowledge of AI, social and ethical implications, workflow analysis for AI-enabled systems, evidence-based evaluation of AI-based tools, conducting AI-enhanced clinical encounters, and continuing education and quality improvement initiatives related to AI. While both continuing professional development (32) and medical school education (33) have been proposed venues for implementing these competencies, strategies for training the mental health workforce for impending AI interactions and influences remain to be determined (4).

Additionally, the findings of this study suggest mental health professionals may need to be especially prepared for vulnerable patient populations to express greater reticence about AI being used in their care. These conversations will be critical as failure to engage these populations in technologies that have a demonstrated benefit with respect to mental health outcomes could actually widen disparities (26). For example, we found that older generations expressed greater concern and discomfort with specific uses of AI in mental healthcare. This is salient because of the known underdiagnosis of mental illnesses (particularly racial and ethnic minorities), distinct symptom presentation, and lower utilization of mental health services among older adults (34). While AI may be poised to address these issues through improved disease detection, caregiver support, and novel solutions to reduce loneliness, failure to engage older adults could further exacerbate these disparities (34).

Limitations of this study include the limited generalizability of the sample, especially with respect to race and ethnicity. Oversampling of racially and ethnically minoritized adults may be important in future work to better understand differences in attitudes toward AI. Moreover, we only examined overall differences and did not apply corrections to account for multiple comparisons. Further, we did not report on group post-hoc comparisons due to small sample sizes in some cells.

## Conclusion

Multiple vulnerable subgroups of U.S. adults perceive potential harms related to AI being used in mental healthcare, place importance on upholding bioethical constructs related to AI use in mental healthcare, and would blame or reduce trust in multiple parties, including mental healthcare professionals, if harm or conflicting assessments resulted from AI. Future research examining strategies to uphold bioethical constructs in AI implementation and support clinician AI literacy is critical for optimal patient and clinician interactions with AI in the future of mental healthcare.

## Data Availability

The raw data supporting the conclusions of this article will be made available by the authors, without undue reservation.
