# Supplementary material for: Differing perspectives on artificial intelligence in mental healthcare among patients: a cross-sectional survey study
Source: Front Digit Health. 2024 Nov 29;6:1410758. doi: 10.3389/fdgth.2024.1410758 (PMC11638230; doi:10.3389/fdgth.2024.1410758)
Supplement: Supplementary file 2 [file Datasheet1.docx]

| **Supplementary Table 1:** Differences in perspectives on AI in mental health by mental health history and current rating among survey participants (n=500); n(%) | | | | | |
| --- | --- | --- | --- | --- | --- |
|  | **Mental illness history** | | **Current mental health rating** | | |
|  | **Mental illness history**, n=215 | **No history**, n=271 | **Excellent/very good**, n=204 | **Good**, n=148 | **Fair/poor**, n=146 |
| **General perspectives** |  |  |  |  |  |
| **How much do you know about AI and how it could change mental healthcare?** |  |  |  |  |  |
| I know quite a lot | 3 (1.4%) | 4 (1.5%) | 5 (2.5%) | 0 (0%) | 2 (1.4%) |
| I know a fair amount | 43 (20%) | 46 (17%) | 41 (20%) | 26 (18%) | 25 (17%) |
| I know a little bit | 103 (48%) | 141 (52%) | 88 (43%) | 89 (60%) | 73 (50%) |
| I know almost nothing | 66 (31%) | 80 (30%) | 70 (34%) | 33 (22%) | 46 (32%) |
| **Overall, in the next 5 years, do you think AI will make mental healthcare in the United States?** |  |  |  |  |  |
| Much better | 11 (5.1%) | 22 (8.1%) | 18 (8.8%) | 7 (4.7%) | 9 (6.2%) |
| Somewhat better | 93 (43%) | 116 (43%) | 86 (42%) | 66 (45%) | 61 (42%) |
| Minimal change | 77 (36%) | 85 (31%) | 65 (32%) | 52 (35%) | 51 (35%) |
| Somewhat worse | 13 (6.0%) | 21 (7.7%) | 13 (6.4%) | 12 (8.1%) | 9 (6.2%) |
| Much worse | 1 (0.5%) | 6 (2.2%) | 4 (2.0%) | 2 (1.4%) | 2 (1.4%) |
| Don't know | 20 (9.3%) | 21 (7.7%) | 18 (8.8%) | 9 (6.1%) | 14 (9.6%) |
| **Comfort with AI** |  |  |  |  |  |
| **AI, instead of a mental health professional, performing a mental health assessment** |  |  |  |  |  |
| Very comfortable | 16 (7.4%) | 27 (10.0%) | 21 (10%) | 8 (5.4%) | 14 (9.6%) |
| Somewhat comfortable | 85 (40%) | 101 (37%) | 81 (40%) | 58 (39%) | 52 (36%) |
| Somewhat uncomfortable | 64 (30%) | 84 (31%) | 57 (28%) | 49 (33%) | 47 (32%) |
| Very uncomfortable | 48 (22%) | 54 (20%) | 42 (21%) | 33 (22%) | 30 (21%) |
| Don't know | 2 (0.9%) | 5 (1.8%) | 3 (1.5%) | 0 (0%) | 3 (2.1%) |
| **AI, instead of a mental health professional, making a diagnosis of clinical depression** |  |  |  |  |  |
| Very comfortable | 13 (6.0%) | 13 (4.8%) | 12 (5.9%) | 6 (4.1%) | 8 (5.5%) |
| Somewhat comfortable | 50 (23%) | 76 (28%) | 50 (25%) | 36 (24%) | 42 (29%) |
| Somewhat uncomfortable | 73 (34%) | 81 (30%) | 61 (30%) | 47 (32%) | 49 (34%) |
| Very uncomfortable | 79 (37%) | 91 (34%) | 76 (37%) | 58 (39%) | 43 (29%) |
| Don't know | 0 (0%) | 10 (3.7%) | 5 (2.5%) | 1 (0.7%) | 4 (2.7%) |
| **AI, instead of a mental health professional, telling you that you are clinically depressed** |  |  |  |  |  |
| Very comfortable | 11 (5.1%) | 18 (6.6%) | 12 (5.9%) | 6 (4.1%) | 11 (7.5%) |
| Somewhat comfortable | 56 (26%) | 60 (22%) | 43 (21%) | 34 (23%) | 42 (29%) |
| Somewhat uncomfortable | 59 (27%) | 83 (31%) | 61 (30%) | 37 (25%) | 49 (34%) |
| Very uncomfortable | 86 (40%) | 101 (37%) | 84 (41%) | 66 (45%) | 41 (28%) |
| Don't know | 3 (1.4%) | 9 (3.3%) | 4 (2.0%) | 5 (3.4%) | 3 (2.1%) |
| **AI, instead of a mental health professional, making a diagnosis of bi-polar disorder** |  |  |  |  |  |
| Very comfortable | 11 (5.1%) | 15 (5.5%) | 10 (4.9%) | 7 (4.7%) | 9 (6.2%) |
| Somewhat comfortable | 36 (17%) | 55 (20%) | 37 (18%) | 26 (18%) | 31 (21%) |
| Somewhat uncomfortable | 62 (29%) | 73 (27%) | 60 (29%) | 32 (22%) | 44 (30%) |
| Very uncomfortable | 104 (48%) | 116 (43%) | 92 (45%) | 77 (52%) | 59 (40%) |
| Don't know | 2 (0.9%) | 12 (4.4%) | 5 (2.5%) | 6 (4.1%) | 3 (2.1%) |
| **AI, instead of a mental health professional, telling you that you have bi-polar disorder** |  |  |  |  |  |
| Very comfortable | 9 (4.2%) | 13 (4.8%) | 9 (4.4%) | 5 (3.4%) | 9 (6.2%) |
| Somewhat comfortable | 33 (15%) | 43 (16%) | 28 (14%) | 24 (16%) | 25 (17%) |
| Somewhat uncomfortable | 65 (30%) | 85 (31%) | 68 (33%) | 35 (24%) | 50 (34%) |
| Very uncomfortable | 105 (49%) | 119 (44%) | 94 (46%) | 78 (53%) | 59 (40%) |
| Don't know | 3 (1.4%) | 11 (4.1%) | 5 (2.5%) | 6 (4.1%) | 3 (2.1%) |
| **AI, instead of a mental health professional, recommending a general wellness or stress-management strategy** |  |  |  |  |  |
| Very comfortable | 62 (29%) | 71 (26%) | 58 (28%) | 41 (28%) | 34 (23%) |
| Somewhat comfortable | 93 (43%) | 124 (46%) | 83 (41%) | 66 (45%) | 74 (51%) |
| Somewhat uncomfortable | 32 (15%) | 40 (15%) | 34 (17%) | 23 (16%) | 18 (12%) |
| Very uncomfortable | 24 (11%) | 30 (11%) | 27 (13%) | 16 (11%) | 14 (9.6%) |
| Don't know | 4 (1.9%) | 6 (2.2%) | 2 (1.0%) | 2 (1.4%) | 6 (4.1%) |
| **AI, instead of a mental health professional, recommending a talk therapy** |  |  |  |  |  |
| Very comfortable | 53 (25%) | 58 (21%) | 52 (25%) | 32 (22%) | 31 (21%) |
| Somewhat comfortable | 95 (44%) | 115 (42%) | 83 (41%) | 61 (41%) | 67 (46%) |
| Somewhat uncomfortable | 29 (13%) | 56 (21%) | 35 (17%) | 29 (20%) | 24 (16%) |
| Very uncomfortable | 33 (15%) | 38 (14%) | 32 (16%) | 22 (15%) | 21 (14%) |
| Don't know | 5 (2.3%) | 4 (1.5%) | 2 (1.0%) | 4 (2.7%) | 3 (2.1%) |
| **AI, instead of a mental health professional, recommending a medication** |  |  |  |  |  |
| Very comfortable | 12 (5.6%) | 21 (7.7%) | 16 (7.8%) | 6 (4.1%) | 11 (7.5%) |
| Somewhat comfortable | 40 (19%) | 60 (22%) | 39 (19%) | 35 (24%) | 28 (19%) |
| Somewhat uncomfortable | 65 (30%) | 81 (30%) | 57 (28%) | 40 (27%) | 51 (35%) |
| Very uncomfortable | 94 (44%) | 105 (39%) | 88 (43%) | 65 (44%) | 54 (37%) |
| Don't know | 4 (1.9%) | 4 (1.5%) | 4 (2.0%) | 2 (1.4%) | 2 (1.4%) |
| **AI, instead of a mental health professional, predicting a patient’s risk for suicide** |  |  |  |  |  |
| Very comfortable | 15 (7.0%) | 24 (8.9%) | 18 (8.8%) | 11 (7.4%) | 10 (6.8%) |
| Somewhat comfortable | 36 (17%) | 61 (23%) | 38 (19%) | 35 (24%) | 27 (18%) |
| Somewhat uncomfortable | 62 (29%) | 71 (26%) | 58 (28%) | 34 (23%) | 44 (30%) |
| Very uncomfortable | 88 (41%) | 102 (38%) | 84 (41%) | 58 (39%) | 54 (37%) |
| Don't know | 14 (6.5%) | 13 (4.8%) | 6 (2.9%) | 10 (6.8%) | 11 (7.5%) |
| **AI, instead of a mental health professional, predicting a patient’s risk of engaging in violent behavior** |  |  |  |  |  |
| Very comfortable | 15 (7.0%) | 23 (8.5%) | 20 (9.8%) | 9 (6.1%) | 9 (6.2%) |
| Somewhat comfortable | 41 (19%) | 68 (25%) | 37 (18%) | 38 (26%) | 37 (25%) |
| Somewhat uncomfortable | 63 (29%) | 59 (22%) | 58 (28%) | 30 (20%) | 38 (26%) |
| Very uncomfortable | 86 (40%) | 105 (39%) | 84 (41%) | 61 (41%) | 51 (35%) |
| Don't know | 10 (4.7%) | 16 (5.9%) | 5 (2.5%) | 10 (6.8%) | 11 (7.5%) |
| **Sharing sensitive information with a human mental health professional** |  |  |  |  |  |
| Very comfortable | 90 (42%) | 88 (32%) | 84 (41%) | 49 (33%) | 48 (33%) |
| Somewhat comfortable | 80 (37%) | 122 (45%) | 81 (40%) | 70 (47%) | 56 (38%) |
| Somewhat uncomfortable | 38 (18%) | 40 (15%) | 29 (14%) | 24 (16%) | 28 (19%) |
| Very uncomfortable | 7 (3.3%) | 20 (7.4%) | 10 (4.9%) | 5 (3.4%) | 13 (8.9%) |
| Don't know | 0 (0%) | 1 (0.4%) | 0 (0%) | 0 (0%) | 1 (0.7%) |
| **Sharing sensitive information with an AI chatbot** |  |  |  |  |  |
| Very comfortable | 33 (15%) | 39 (14%) | 39 (19%) | 13 (8.8%) | 20 (14%) |
| Somewhat comfortable | 66 (31%) | 96 (35%) | 65 (32%) | 57 (39%) | 43 (29%) |
| Somewhat uncomfortable | 60 (28%) | 66 (24%) | 43 (21%) | 43 (29%) | 43 (29%) |
| Very uncomfortable | 53 (25%) | 65 (24%) | 55 (27%) | 32 (22%) | 38 (26%) |
| Don't know | 3 (1.4%) | 5 (1.8%) | 2 (1.0%) | 3 (2.0%) | 2 (1.4%) |
| **Sharing sensitive information to help improve AI programs that treat disease** |  |  |  |  |  |
| Very comfortable | 53 (25%) | 58 (21%) | 54 (26%) | 26 (18%) | 32 (22%) |
| Somewhat comfortable | 81 (38%) | 104 (38%) | 67 (33%) | 67 (45%) | 54 (37%) |
| Somewhat uncomfortable | 48 (22%) | 50 (18%) | 34 (17%) | 35 (24%) | 30 (21%) |
| Very uncomfortable | 22 (10%) | 47 (17%) | 40 (20%) | 14 (9.5%) | 20 (14%) |
| Don't know | 11 (5.1%) | 12 (4.4%) | 9 (4.4%) | 6 (4.1%) | 10 (6.8%) |
| **Specific concerns** |  |  |  |  |  |
| **That my mental health information will not be kept confidential** |  |  |  |  |  |
| Very concerned | 47 (22%) | 61 (23%) | 46 (23%) | 30 (20%) | 36 (25%) |
| Somewhat concerned | 78 (36%) | 104 (38%) | 71 (35%) | 64 (43%) | 54 (37%) |
| Not concerned | 88 (41%) | 103 (38%) | 83 (41%) | 54 (36%) | 55 (38%) |
| Don't know | 2 (0.9%) | 3 (1.1%) | 4 (2.0%) | 0 (0%) | 1 (0.7%) |
| **That the AI will make the wrong diagnosis about my mental health*** |  |  |  |  |  |
| Very concerned | 100 (47%) | 116 (43%) | 83 (41%) | 74 (50%) | 62 (42%) |
| Somewhat concerned | 102 (48%) | 119 (44%) | 93 (46%) | 65 (44%) | 70 (48%) |
| Not concerned | 12 (5.6%) | 32 (12%) | 25 (12%) | 7 (4.8%) | 14 (9.6%) |
| Don't know | 0 (0%) | 4 (1.5%) | 3 (1.5%) | 1 (0.7%) | 0 (0%) |
| (Missing) | 1 | 0 | 0 | 1 | 0 |
| **That the AI will lead to me getting inappropriate treatment for my mental health** |  |  |  |  |  |
| Very concerned | 105 (49%) | 112 (41%) | 87 (43%) | 69 (47%) | 65 (45%) |
| Somewhat concerned | 90 (42%) | 117 (43%) | 82 (40%) | 69 (47%) | 61 (42%) |
| Not concerned | 20 (9.3%) | 38 (14%) | 32 (16%) | 8 (5.4%) | 19 (13%) |
| Don't know | 0 (0%) | 4 (1.5%) | 3 (1.5%) | 1 (0.7%) | 1 (0.7%) |
| (Missing) | 0 (0%) | 0 (0%) | 0 | 1 | 0 |
| **That AI will mean I spend less time with my mental health professional** |  |  |  |  |  |
| Very concerned | 88 (41%) | 85 (31%) | 74 (36%) | 53 (36%) | 49 (34%) |
| Somewhat concerned | 71 (33%) | 92 (34%) | 66 (32%) | 60 (41%) | 43 (29%) |
| Not concerned | 49 (23%) | 79 (29%) | 56 (27%) | 31 (21%) | 44 (30%) |
| Don't know | 7 (3.3%) | 15 (5.5%) | 8 (3.9%) | 4 (2.7%) | 10 (6.8%) |
| **That AI will lead to my mental health provider not knowing me as well** |  |  |  |  |  |
| Very concerned | 102 (47%) | 104 (39%) | 91 (45%) | 63 (43%) | 54 (37%) |
| Somewhat concerned | 79 (37%) | 112 (41%) | 72 (35%) | 61 (41%) | 67 (46%) |
| Not concerned | 31 (14%) | 47 (17%) | 36 (18%) | 22 (15%) | 21 (14%) |
| Don't know | 3 (1.4%) | 7 (2.6%) | 5 (2.5%) | 2 (1.4%) | 3 (2.1%) |
| (Missing) | 0 | 1 | 0 | 0 | 1 |
| **That AI will increase my mental health care costs** |  |  |  |  |  |
| Very concerned | 42 (20%) | 42 (16%) | 37 (18%) | 22 (15%) | 25 (17%) |
| Somewhat concerned | 58 (27%) | 67 (25%) | 44 (22%) | 38 (26%) | 48 (33%) |
| Not concerned | 98 (46%) | 134 (50%) | 104 (51%) | 72 (49%) | 62 (42%) |
| Don't know | 17 (7.9%) | 27 (10%) | 19 (9.3%) | 15 (10%) | 11 (7.5%) |
| (Missing) | 0 | 1 | 0 | 1 | 0 |
| **Explainability and transparency** |  |  |  |  |  |
| **Importance of being told when AI played a big role in mental health diagnosis or treatment** |  |  |  |  |  |
| Not important | 8 (3.7%) | 5 (1.8%) | 3 (1.5%) | 5 (3.4%) | 5 (3.4%) |
| Somewhat important | 44 (20%) | 60 (22%) | 44 (22%) | 30 (20%) | 37 (25%) |
| Very important | 158 (73%) | 201 (74%) | 153 (75%) | 110 (74%) | 100 (68%) |
| Don't know | 5 (2.3%) | 5 (1.8%) | 4 (2.0%) | 3 (2.0%) | 4 (2.7%) |
| **Importance of being told when AI played a small role in mental health diagnosis or treatment** |  |  |  |  |  |
| Not important | 18 (8.4%) | 20 (7.4%) | 13 (6.4%) | 15 (10%) | 11 (7.5%) |
| Somewhat important | 87 (40%) | 104 (38%) | 83 (41%) | 56 (38%) | 59 (40%) |
| Very important | 105 (49%) | 142 (52%) | 106 (52%) | 75 (51%) | 71 (49%) |
| Don't know | 5 (2.3%) | 5 (1.8%) | 2 (1.0%) | 2 (1.4%) | 5 (3.4%) |
| **[Given scenario where AI recommends anti-depressant] Importance of being told that AI helped make this decision** |  |  |  |  |  |
| Not important | 12 (5.6%) | 14 (5.2%) | 10 (4.9%) | 8 (5.4%) | 8 (5.5%) |
| Somewhat important | 56 (26%) | 54 (20%) | 44 (22%) | 32 (22%) | 36 (25%) |
| Very important | 143 (67%) | 202 (75%) | 148 (73%) | 107 (72%) | 99 (68%) |
| Don't know | 4 (1.9%) | 1 (0.4%) | 2 (1.0%) | 1 (0.7%) | 3 (2.1%) |
| **Comfort receiving a diagnosis from AI that is 90% accurate but unexplainable** |  |  |  |  |  |
| Very comfortable | 8 (3.7%) | 7 (2.6%) | 6 (2.9%) | 3 (2.0%) | 6 (4.1%) |
| Somewhat comfortable | 32 (15%) | 60 (22%) | 47 (23%) | 20 (14%) | 27 (18%) |
| Somewhat uncomfortable | 78 (36%) | 99 (37%) | 72 (35%) | 59 (40%) | 52 (36%) |
| Very uncomfortable | 93 (43%) | 102 (38%) | 78 (38%) | 65 (44%) | 55 (38%) |
| Don't know | 4 (1.9%) | 3 (1.1%) | 1 (0.5%) | 1 (0.7%) | 6 (4.1%) |
| **Comfort receiving a diagnosis from AI that is 98% accurate but unexplainable** |  |  |  |  |  |
| Very comfortable | 25 (12%) | 37 (14%) | 31 (15%) | 10 (6.8%) | 22 (15%) |
| Somewhat comfortable | 61 (28%) | 75 (28%) | 49 (24%) | 43 (29%) | 46 (32%) |
| Somewhat uncomfortable | 74 (34%) | 91 (34%) | 73 (36%) | 56 (38%) | 43 (29%) |
| Very uncomfortable | 51 (24%) | 63 (23%) | 50 (25%) | 37 (25%) | 29 (20%) |
| Don't know | 4 (1.9%) | 5 (1.8%) | 1 (0.5%) | 2 (1.4%) | 6 (4.1%) |
| **Trust and responsibility** |  |  |  |  |  |
| **[Given scenario where AI and doctor opinion conflicts] How does the computer program affect your view?** |  |  |  |  |  |
| It would not affect my trust of the mental health professional’s  assessment | 27 (13%) | 56 (21%) | 47 (23%) | 18 (12%) | 20 (14%) |
| It would make me question the mental health professional’s  assessment | 120 (56%) | 136 (50%) | 96 (47%) | 77 (52%) | 91 (62%) |
| I do not know if it would change my view of the mental health  professional’s assessment | 62 (29%) | 72 (27%) | 57 (28%) | 47 (32%) | 32 (22%) |
| Don't know | 6 (2.8%) | 7 (2.6%) | 4 (2.0%) | 6 (4.1%) | 3 (2.1%) |
| **Responsibility for medical errors resulting from AI (select all that apply)** |  |  |  |  |  |
| Mental health professional | 184 (86%) | 218 (80%) | 175 (86%) | 120 (81%) | 115 (79%) |
| Company that made the computer program | 67 (31%) | 108 (40%) | 69 (34%) | 52 (35%) | 60 (41%) |
| Hospital or clinic that bought the computer program | 51 (24%) | 97 (36%) | 62 (30%) | 39 (26%) | 51 (35%) |
| Government agency that approved the computer program | 39 (18%) | 73 (27%) | 43 (21%) | 31 (21%) | 40 (27%) |
| Someone else | 11 (5.1%) | 6 (2.2%) | 4 (2.0%) | 9 (6.1%) | 4 (2.7%) |
| No one | 1 (0.5%) | 0 (0%) | 0 (0%) | 0 (0%) | 1 (0.7%) |
| Don't know | 9 (4.2%) | 17 (6.3%) | 9 (4.4%) | 6 (4.1%) | 12 (8.2%) |
| **Blame for medical errors resulting from AI (select all that apply)** |  |  |  |  |  |
| Mental health professional | 174 (81%) | 222 (82%) | 168 (82%) | 119 (80%) | 119 (82%) |
| Company that made the computer program | 59 (27%) | 104 (38%) | 69 (34%) | 46 (31%) | 52 (36%) |
| Hospital or clinic that bought the computer program | 43 (20%) | 80 (30%) | 53 (26%) | 33 (22%) | 37 (25%) |
| Government agency that approved the computer program | 37 (17%) | 66 (24%) | 44 (22%) | 27 (18%) | 32 (22%) |
| Someone else | 5 (2.3%) | 5 (1.8%) | 3 (1.5%) | 5 (3.4%) | 1 (0.7%) |
| No one | 13 (6.0%) | 5 (1.8%) | 6 (2.9%) | 9 (6.1%) | 4 (2.7%) |
| Don't know | 7 (3.3%) | 7 (2.6%) | 2 (1.0%) | 6 (4.1%) | 7 (4.8%) |
| **Responsibility for checking that AI is safe (select all that apply)** |  |  |  |  |  |
| Mental health professional | 51 (24%) | 79 (29%) | 60 (29%) | 40 (27%) | 35 (24%) |
| Company that made the computer program | 143 (67%) | 179 (66%) | 125 (61%) | 102 (69%) | 101 (69%) |
| Hospital or clinic that bought the computer program | 115 (53%) | 158 (58%) | 107 (52%) | 92 (62%) | 83 (57%) |
| Government agency that approved the computer program | 105 (49%) | 129 (48%) | 90 (44%) | 73 (49%) | 75 (51%) |
| Someone else | 4 (1.9%) | 3 (1.1%) | 3 (1.5%) | 3 (2.0%) | 1 (0.7%) |
| No one | 6 (2.8%) | 11 (4.1%) | 10 (4.9%) | 3 (2.0%) | 4 (2.7%) |
| Don't know | 5 (2.3%) | 8 (3.0%) | 6 (2.9%) | 3 (2.0%) | 3 (2.1%) |
| **Importance of bioethical constructs** |  |  |  |  |  |
| **How important in general: That people are able to make up their own mind about their risk for depression based on AI output [autonomy]** |  |  |  |  |  |
| Very important | 127 (59%) | 147 (54%) | 108 (53%) | 76 (51%) | 95 (65%) |
| Somewhat important | 72 (33%) | 100 (37%) | 82 (40%) | 54 (36%) | 42 (29%) |
| Not important | 7 (3.3%) | 15 (5.5%) | 10 (4.9%) | 8 (5.4%) | 4 (2.7%) |
| Don't know | 9 (4.2%) | 9 (3.3%) | 4 (2.0%) | 10 (6.8%) | 5 (3.4%) |
| **How important in general: That AI will improve depressive symptoms [beneficence]** |  |  |  |  |  |
| Very important | 93 (43%) | 130 (48%) | 85 (42%) | 77 (52%) | 65 (45%) |
| Somewhat important | 87 (40%) | 106 (39%) | 86 (42%) | 50 (34%) | 63 (43%) |
| Not important | 15 (7.0%) | 22 (8.1%) | 17 (8.3%) | 10 (6.8%) | 10 (6.8%) |
| Don't know | 20 (9.3%) | 13 (4.8%) | 16 (7.8%) | 11 (7.4%) | 8 (5.5%) |
| **How important in general: That AI will reduce the chance of negative outcomes [non-maleficence]** |  |  |  |  |  |
| Very important | 158 (73%) | 173 (64%) | 124 (61%) | 103 (70%) | 109 (75%) |
| Somewhat important | 44 (20%) | 87 (32%) | 67 (33%) | 38 (26%) | 31 (21%) |
| Not important | 8 (3.7%) | 8 (3.0%) | 8 (3.9%) | 4 (2.7%) | 4 (2.7%) |
| Don't know | 5 (2.3%) | 3 (1.1%) | 5 (2.5%) | 3 (2.0%) | 2 (1.4%) |
| **How important in general: That people can understand how likely it is that they develop depression in the next year according to the AI [justice]** |  |  |  |  |  |
| Very important | 139 (65%) | 183 (68%) | 128 (63%) | 101 (68%) | 99 (68%) |
| Somewhat important | 58 (27%) | 71 (26%) | 59 (29%) | 39 (26%) | 36 (25%) |
| Not important | 6 (2.8%) | 8 (3.0%) | 8 (3.9%) | 4 (2.7%) | 3 (2.1%) |
| Don't know | 12 (5.6%) | 9 (3.3%) | 9 (4.4%) | 4 (2.7%) | 8 (5.5%) |
| **How important in general: That AI does not reduce people’s trust in their mental health care professionals [trust]** |  |  |  |  |  |
| Very important | 62 (29%) | 87 (32%) | 63 (31%) | 41 (28%) | 47 (32%) |
| Somewhat important | 99 (46%) | 136 (50%) | 105 (51%) | 70 (47%) | 68 (47%) |
| Not important | 34 (16%) | 37 (14%) | 25 (12%) | 25 (17%) | 22 (15%) |
| Don't know | 20 (9.3%) | 11 (4.1%) | 11 (5.4%) | 12 (8.1%) | 9 (6.2%) |
| **How important in general: That people are aware of how their personal data is being used for AI [privacy]** |  |  |  |  |  |
| Very important | 165 (77%) | 190 (70%) | 147 (72%) | 108 (73%) | 107 (73%) |
| Somewhat important | 36 (17%) | 55 (20%) | 32 (16%) | 31 (21%) | 27 (18%) |
| Not important | 7 (3.3%) | 21 (7.7%) | 19 (9.3%) | 6 (4.1%) | 5 (3.4%) |
| Don't know | 7 (3.3%) | 5 (1.8%) | 6 (2.9%) | 3 (2.0%) | 7 (4.8%) |
| **How important in general: That people can understand which of their individual risk factors for depression are used by the AI [transparency]** |  |  |  |  |  |
| Very important | 184 (86%) | 220 (81%) | 167 (82%) | 125 (84%) | 118 (81%) |
| Somewhat important | 22 (10%) | 42 (15%) | 26 (13%) | 21 (14%) | 21 (14%) |
| Not important | 2 (0.9%) | 8 (3.0%) | 6 (2.9%) | 1 (0.7%) | 3 (2.1%) |
| Don't know | 7 (3.3%) | 1 (0.4%) | 5 (2.5%) | 1 (0.7%) | 4 (2.7%) |
| **How important to you: That you are able to make up your own mind about your risk for depression based on AI output [autonomy]** |  |  |  |  |  |
| Very important | 149 (69%) | 169 (62%) | 127 (62%) | 91 (61%) | 106 (73%) |
| Somewhat important | 55 (26%) | 87 (32%) | 66 (32%) | 48 (32%) | 33 (23%) |
| Not important | 5 (2.3%) | 11 (4.1%) | 8 (3.9%) | 3 (2.0%) | 5 (3.4%) |
| Don't know | 6 (2.8%) | 4 (1.5%) | 3 (1.5%) | 6 (4.1%) | 2 (1.4%) |
| **How important to you: That AI will improve your depression/depressive symptoms [beneficence]** |  |  |  |  |  |
| Very important | 127 (59%) | 171 (63%) | 118 (58%) | 94 (64%) | 90 (62%) |
| Somewhat important | 60 (28%) | 79 (29%) | 63 (31%) | 39 (26%) | 42 (29%) |
| Not important | 14 (6.5%) | 16 (5.9%) | 14 (6.9%) | 10 (6.8%) | 8 (5.5%) |
| Don't know | 14 (6.5%) | 5 (1.8%) | 9 (4.4%) | 5 (3.4%) | 6 (4.1%) |
| **How important to you: That AI will decrease the chance of negative outcomes [non-maleficence]** |  |  |  |  |  |
| Very important | 164 (76%) | 184 (68%) | 137 (67%) | 106 (72%) | 113 (77%) |
| Somewhat important | 45 (21%) | 74 (27%) | 58 (28%) | 37 (25%) | 28 (19%) |
| Not important | 4 (1.9%) | 9 (3.3%) | 6 (2.9%) | 4 (2.7%) | 3 (2.1%) |
| Don't know | 2 (0.9%) | 4 (1.5%) | 3 (1.5%) | 1 (0.7%) | 2 (1.4%) |
| **How important to you: That you can understand how likely it is that you develop depression within the next year according to the AI [justice]** |  |  |  |  |  |
| Very important | 148 (69%) | 188 (69%) | 131 (64%) | 107 (72%) | 105 (72%) |
| Somewhat important | 49 (23%) | 68 (25%) | 58 (28%) | 33 (22%) | 30 (21%) |
| Not important | 6 (2.8%) | 8 (3.0%) | 7 (3.4%) | 4 (2.7%) | 4 (2.7%) |
| Don't know | 12 (5.6%) | 7 (2.6%) | 8 (3.9%) | 4 (2.7%) | 7 (4.8%) |
| **How important to you: That using AI does not reduce your trust in your mental health care provider [trust]** |  |  |  |  |  |
| Very important | 79 (37%) | 100 (37%) | 78 (38%) | 54 (36%) | 54 (37%) |
| Somewhat important | 89 (41%) | 122 (45%) | 88 (43%) | 59 (40%) | 68 (47%) |
| Not important | 31 (14%) | 34 (13%) | 26 (13%) | 24 (16%) | 15 (10%) |
| Don't know | 16 (7.4%) | 15 (5.5%) | 12 (5.9%) | 11 (7.4%) | 9 (6.2%) |
| **How important to you: That you are aware of how your personal data is being used for AI [privacy]** |  |  |  |  |  |
| Very important | 158 (73%) | 183 (68%) | 141 (69%) | 107 (72%) | 100 (68%) |
| Somewhat important | 41 (19%) | 61 (23%) | 41 (20%) | 33 (22%) | 32 (22%) |
| Not important | 10 (4.7%) | 24 (8.9%) | 16 (7.8%) | 8 (5.4%) | 9 (6.2%) |
| Don't know | 6 (2.8%) | 3 (1.1%) | 6 (2.9%) | 0 (0%) | 5 (3.4%) |
| **How important to you: That you can understand which of your individual risk factors for depression are used by the AI [transparency]** |  |  |  |  |  |
| Very important | 178 (83%) | 215 (79%) | 159 (78%) | 121 (82%) | 120 (82%) |
| Somewhat important | 30 (14%) | 44 (16%) | 33 (16%) | 24 (16%) | 20 (14%) |
| Not important | 5 (2.3%) | 11 (4.1%) | 10 (4.9%) | 3 (2.0%) | 4 (2.7%) |
| Don't know | 2 (0.9%) | 1 (0.4%) | 2 (1.0%) | 0 (0%) | 2 (1.4%) |
| *Statistical significance represented by grey boxes. Statistical significance determined using Fisher's Exact Test for Count Data with simulated p-value (based on 2000 replicates).* | | | | | |

| **Supplementary Table 2:** Differences in perspectives on AI in mental health by demographic characteristics (age and gender) among survey participants (n=500); n(%) | | | | | | | |
| --- | --- | --- | --- | --- | --- | --- | --- |
|  | **Age (generation)** | | | | | **Gender** | |
|  | **Gen Z**, n=51 | **Millennial**, n=155 | **Gen X**, n=132 | **Boomer**, n=152 | **Silent**, n=10 | **Female**, n=249 | **Male**, n=238 |
| **General perspectives** |  |  |  |  |  |  |  |
| **How much do you know about AI and how it could change mental healthcare?** |  |  |  |  |  |  |  |
| I know quite a lot | 2 (3.9%) | 1 (0.6%) | 3 (2.3%) | 1 (0.7%) | 0 (0%) | 1 (0.4%) | 6 (2.5%) |
| I know a fair amount | 7 (14%) | 38 (25%) | 26 (20%) | 20 (13%) | 1 (10%) | 28 (11%) | 63 (26%) |
| I know a little bit | 31 (61%) | 78 (50%) | 64 (48%) | 74 (49%) | 5 (50%) | 129 (52%) | 114 (48%) |
| I know almost nothing | 11 (22%) | 38 (25%) | 39 (30%) | 57 (38%) | 4 (40%) | 91 (37%) | 55 (23%) |
| **Overall, in the next 5 years, do you think AI will make mental healthcare in the United States?** |  |  |  |  |  |  |  |
| Much better | 6 (12%) | 9 (5.8%) | 9 (6.8%) | 7 (4.6%) | 3 (30%) | 11 (4.4%) | 22 (9.2%) |
| Somewhat better | 19 (37%) | 75 (48%) | 57 (43%) | 60 (39%) | 2 (20%) | 102 (41%) | 107 (45%) |
| Minimal change | 21 (41%) | 49 (32%) | 39 (30%) | 57 (38%) | 4 (40%) | 85 (34%) | 80 (34%) |
| Somewhat worse | 4 (7.8%) | 8 (5.2%) | 10 (7.6%) | 12 (7.9%) | 0 (0%) | 19 (7.6%) | 14 (5.9%) |
| Much worse | 0 (0%) | 1 (0.6%) | 4 (3.0%) | 3 (2.0%) | 0 (0%) | 4 (1.6%) | 3 (1.3%) |
| Don't know | 1 (2.0%) | 13 (8.4%) | 13 (9.8%) | 13 (8.6%) | 1 (10%) | 28 (11%) | 12 (5.0%) |
| **Comfort with AI** |  |  |  |  |  |  |  |
| **AI, instead of a mental health professional, performing a mental health assessment** |  |  |  |  |  |  |  |
| Very comfortable | 3 (5.9%) | 9 (5.8%) | 17 (13%) | 13 (8.6%) | 1 (10%) | 20 (8.0%) | 21 (8.8%) |
| Somewhat comfortable | 21 (41%) | 62 (40%) | 52 (39%) | 51 (34%) | 5 (50%) | 99 (40%) | 88 (37%) |
| Somewhat uncomfortable | 14 (27%) | 54 (35%) | 37 (28%) | 45 (30%) | 3 (30%) | 67 (27%) | 83 (35%) |
| Very uncomfortable | 11 (22%) | 29 (19%) | 24 (18%) | 41 (27%) | 1 (10%) | 60 (24%) | 43 (18%) |
| Don't know | 2 (3.9%) | 1 (0.6%) | 2 (1.5%) | 2 (1.3%) | 0 (0%) | 3 (1.2%) | 3 (1.3%) |
| **AI, instead of a mental health professional, making a diagnosis of clinical depression** |  |  |  |  |  |  |  |
| Very comfortable | 3 (5.9%) | 10 (6.5%) | 8 (6.1%) | 4 (2.6%) | 1 (10%) | 12 (4.8%) | 13 (5.5%) |
| Somewhat comfortable | 12 (24%) | 37 (24%) | 39 (30%) | 38 (25%) | 2 (20%) | 56 (22%) | 66 (28%) |
| Somewhat uncomfortable | 23 (45%) | 55 (35%) | 36 (27%) | 40 (26%) | 4 (40%) | 79 (32%) | 76 (32%) |
| Very uncomfortable | 12 (24%) | 50 (32%) | 46 (35%) | 67 (44%) | 3 (30%) | 99 (40%) | 76 (32%) |
| Don't know | 1 (2.0%) | 3 (1.9%) | 3 (2.3%) | 3 (2.0%) | 0 (0%) | 3 (1.2%) | 7 (2.9%) |
| **AI, instead of a mental health professional, telling you that you are clinically depressed** |  |  |  |  |  |  |  |
| Very comfortable | 4 (7.8%) | 10 (6.5%) | 9 (6.8%) | 5 (3.3%) | 1 (10%) | 11 (4.4%) | 17 (7.1%) |
| Somewhat comfortable | 8 (16%) | 41 (26%) | 41 (31%) | 28 (18%) | 1 (10%) | 58 (23%) | 55 (23%) |
| Somewhat uncomfortable | 22 (43%) | 47 (30%) | 31 (23%) | 43 (28%) | 4 (40%) | 71 (29%) | 74 (31%) |
| Very uncomfortable | 16 (31%) | 54 (35%) | 47 (36%) | 72 (47%) | 4 (40%) | 103 (41%) | 87 (37%) |
| Don't know | 1 (2.0%) | 3 (1.9%) | 4 (3.0%) | 4 (2.6%) | 0 (0%) | 6 (2.4%) | 5 (2.1%) |
| **AI, instead of a mental health professional, making a diagnosis of bi-polar disorder** |  |  |  |  |  |  |  |
| Very comfortable | 4 (7.8%) | 7 (4.5%) | 10 (7.6%) | 4 (2.6%) | 1 (10%) | 12 (4.8%) | 13 (5.5%) |
| Somewhat comfortable | 10 (20%) | 31 (20%) | 26 (20%) | 25 (16%) | 2 (20%) | 37 (15%) | 52 (22%) |
| Somewhat uncomfortable | 15 (29%) | 49 (32%) | 31 (23%) | 39 (26%) | 3 (30%) | 73 (29%) | 63 (26%) |
| Very uncomfortable | 21 (41%) | 64 (41%) | 60 (45%) | 80 (53%) | 4 (40%) | 123 (49%) | 101 (42%) |
| Don't know | 1 (2.0%) | 4 (2.6%) | 5 (3.8%) | 4 (2.6%) | 0 (0%) | 4 (1.6%) | 9 (3.8%) |
| **AI, instead of a mental health professional, telling you that you have bi-polar disorder** |  |  |  |  |  |  |  |
| Very comfortable | 3 (5.9%) | 7 (4.5%) | 10 (7.6%) | 2 (1.3%) | 1 (10%) | 10 (4.0%) | 12 (5.0%) |
| Somewhat comfortable | 8 (16%) | 30 (19%) | 21 (16%) | 18 (12%) | 0 (0%) | 30 (12%) | 42 (18%) |
| Somewhat uncomfortable | 18 (35%) | 52 (34%) | 37 (28%) | 42 (28%) | 4 (40%) | 75 (30%) | 76 (32%) |
| Very uncomfortable | 20 (39%) | 64 (41%) | 59 (45%) | 85 (56%) | 5 (50%) | 130 (52%) | 99 (42%) |
| Don't know | 2 (3.9%) | 2 (1.3%) | 5 (3.8%) | 5 (3.3%) | 0 (0%) | 4 (1.6%) | 9 (3.8%) |
| **AI, instead of a mental health professional, recommending a general wellness or stress-management strategy** |  |  |  |  |  |  |  |
| Very comfortable | 15 (29%) | 39 (25%) | 51 (39%) | 25 (16%) | 4 (40%) | 71 (29%) | 57 (24%) |
| Somewhat comfortable | 21 (41%) | 79 (51%) | 48 (36%) | 73 (48%) | 3 (30%) | 109 (44%) | 111 (47%) |
| Somewhat uncomfortable | 9 (18%) | 18 (12%) | 19 (14%) | 27 (18%) | 2 (20%) | 40 (16%) | 35 (15%) |
| Very uncomfortable | 2 (3.9%) | 17 (11%) | 13 (9.8%) | 24 (16%) | 1 (10%) | 28 (11%) | 28 (12%) |
| Don't know | 4 (7.8%) | 2 (1.3%) | 1 (0.8%) | 3 (2.0%) | 0 (0%) | 1 (0.4%) | 7 (2.9%) |
| **AI, instead of a mental health professional, recommending a talk therapy** |  |  |  |  |  |  |  |
| Very comfortable | 10 (20%) | 38 (25%) | 40 (30%) | 24 (16%) | 3 (30%) | 57 (23%) | 52 (22%) |
| Somewhat comfortable | 22 (43%) | 73 (47%) | 55 (42%) | 58 (38%) | 4 (40%) | 103 (41%) | 106 (45%) |
| Somewhat uncomfortable | 12 (24%) | 19 (12%) | 21 (16%) | 35 (23%) | 1 (10%) | 41 (16%) | 45 (19%) |
| Very uncomfortable | 4 (7.8%) | 21 (14%) | 15 (11%) | 33 (22%) | 2 (20%) | 41 (16%) | 32 (13%) |
| Don't know | 3 (5.9%) | 4 (2.6%) | 1 (0.8%) | 2 (1.3%) | 0 (0%) | 7 (2.8%) | 3 (1.3%) |
| **AI, instead of a mental health professional, recommending a medication** |  |  |  |  |  |  |  |
| Very comfortable | 5 (9.8%) | 8 (5.2%) | 15 (11%) | 4 (2.6%) | 1 (10%) | 12 (4.8%) | 20 (8.4%) |
| Somewhat comfortable | 15 (29%) | 37 (24%) | 22 (17%) | 27 (18%) | 2 (20%) | 47 (19%) | 51 (21%) |
| Somewhat uncomfortable | 15 (29%) | 57 (37%) | 38 (29%) | 37 (24%) | 2 (20%) | 75 (30%) | 71 (30%) |
| Very uncomfortable | 14 (27%) | 51 (33%) | 55 (42%) | 82 (54%) | 5 (50%) | 113 (45%) | 90 (38%) |
| Don't know | 2 (3.9%) | 2 (1.3%) | 2 (1.5%) | 2 (1.3%) | 0 (0%) | 2 (0.8%) | 6 (2.5%) |
| **AI, instead of a mental health professional, predicting a patient’s risk for suicide** |  |  |  |  |  |  |  |
| Very comfortable | 6 (12%) | 13 (8.4%) | 11 (8.3%) | 8 (5.3%) | 1 (10%) | 18 (7.2%) | 20 (8.4%) |
| Somewhat comfortable | 9 (18%) | 36 (23%) | 27 (20%) | 28 (18%) | 0 (0%) | 54 (22%) | 43 (18%) |
| Somewhat uncomfortable | 15 (29%) | 35 (23%) | 44 (33%) | 39 (26%) | 4 (40%) | 64 (26%) | 69 (29%) |
| Very uncomfortable | 19 (37%) | 61 (39%) | 43 (33%) | 69 (45%) | 5 (50%) | 102 (41%) | 91 (38%) |
| Don't know | 2 (3.9%) | 10 (6.5%) | 7 (5.3%) | 8 (5.3%) | 0 (0%) | 11 (4.4%) | 15 (6.3%) |
| **AI, instead of a mental health professional, predicting a patient’s risk of engaging in violent behavior** |  |  |  |  |  |  |  |
| Very comfortable | 4 (7.8%) | 11 (7.1%) | 13 (9.8%) | 8 (5.3%) | 2 (20%) | 19 (7.6%) | 18 (7.6%) |
| Somewhat comfortable | 11 (22%) | 37 (24%) | 32 (24%) | 32 (21%) | 0 (0%) | 56 (22%) | 53 (22%) |
| Somewhat uncomfortable | 18 (35%) | 41 (26%) | 35 (27%) | 30 (20%) | 3 (30%) | 61 (24%) | 63 (26%) |
| Very uncomfortable | 14 (27%) | 58 (37%) | 47 (36%) | 72 (47%) | 5 (50%) | 101 (41%) | 90 (38%) |
| Don't know | 4 (7.8%) | 8 (5.2%) | 5 (3.8%) | 10 (6.6%) | 0 (0%) | 12 (4.8%) | 14 (5.9%) |
| **Sharing sensitive information with a human mental health professional** |  |  |  |  |  |  |  |
| Very comfortable | 15 (29%) | 57 (37%) | 48 (36%) | 55 (36%) | 6 (60%) | 98 (39%) | 79 (33%) |
| Somewhat comfortable | 23 (45%) | 64 (41%) | 49 (37%) | 68 (45%) | 4 (40%) | 101 (41%) | 102 (43%) |
| Somewhat uncomfortable | 9 (18%) | 24 (15%) | 24 (18%) | 25 (16%) | 0 (0%) | 42 (17%) | 38 (16%) |
| Very uncomfortable | 3 (5.9%) | 10 (6.5%) | 11 (8.3%) | 4 (2.6%) | 0 (0%) | 8 (3.2%) | 18 (7.6%) |
| Don't know | 1 (2.0%) | 0 (0%) | 0 (0%) | 0 (0%) | 0 (0%) | 0 (0%) | 1 (0.4%) |
| **Sharing sensitive information with an AI chatbot** |  |  |  |  |  |  |  |
| Very comfortable | 7 (14%) | 18 (12%) | 23 (17%) | 21 (14%) | 3 (30%) | 39 (16%) | 29 (12%) |
| Somewhat comfortable | 15 (29%) | 62 (40%) | 42 (32%) | 43 (28%) | 3 (30%) | 80 (32%) | 81 (34%) |
| Somewhat uncomfortable | 18 (35%) | 40 (26%) | 31 (23%) | 40 (26%) | 1 (10%) | 64 (26%) | 64 (27%) |
| Very uncomfortable | 11 (22%) | 34 (22%) | 34 (26%) | 43 (28%) | 3 (30%) | 62 (25%) | 60 (25%) |
| Don't know | 0 (0%) | 1 (0.6%) | 2 (1.5%) | 5 (3.3%) | 0 (0%) | 4 (1.6%) | 4 (1.7%) |
| **Sharing sensitive information to help improve AI programs that treat disease** |  |  |  |  |  |  |  |
| Very comfortable | 10 (20%) | 37 (24%) | 32 (24%) | 29 (19%) | 4 (40%) | 53 (21%) | 53 (22%) |
| Somewhat comfortable | 18 (35%) | 59 (38%) | 49 (37%) | 60 (39%) | 2 (20%) | 100 (40%) | 84 (35%) |
| Somewhat uncomfortable | 14 (27%) | 27 (17%) | 23 (17%) | 34 (22%) | 2 (20%) | 50 (20%) | 49 (21%) |
| Very uncomfortable | 6 (12%) | 26 (17%) | 22 (17%) | 18 (12%) | 2 (20%) | 31 (12%) | 42 (18%) |
| Don't know | 3 (5.9%) | 6 (3.9%) | 6 (4.5%) | 11 (7.2%) | 0 (0%) | 15 (6.0%) | 10 (4.2%) |
| **Specific concerns** |  |  |  |  |  |  |  |
| **That my mental health information will not be kept confidential** |  |  |  |  |  |  |  |
| Very concerned | 8 (16%) | 40 (26%) | 32 (24%) | 31 (20%) | 1 (10%) | 52 (21%) | 55 (23%) |
| Somewhat concerned | 27 (53%) | 56 (36%) | 47 (36%) | 57 (38%) | 3 (30%) | 97 (39%) | 90 (38%) |
| Not concerned | 15 (29%) | 58 (37%) | 53 (40%) | 61 (40%) | 6 (60%) | 97 (39%) | 92 (39%) |
| Don't know | 1 (2.0%) | 1 (0.6%) | 0 (0%) | 3 (2.0%) | 0 (0%) | 3 (1.2%) | 1 (0.4%) |
| **That the AI will make the wrong diagnosis about my mental health** |  |  |  |  |  |  |  |
| Very concerned | 19 (37%) | 73 (47%) | 52 (39%) | 73 (48%) | 4 (40%) | 121 (49%) | 93 (39%) |
| Somewhat concerned | 26 (51%) | 73 (47%) | 67 (51%) | 57 (38%) | 5 (50%) | 105 (42%) | 118 (50%) |
| Not concerned | 6 (12%) | 8 (5.2%) | 12 (9.1%) | 19 (13%) | 1 (10%) | 22 (8.9%) | 23 (9.7%) |
| Don't know | 0 (0%) | 1 (0.6%) | 1 (0.8%) | 2 (1.3%) | 0 (0%) | 0 (0%) | 4 (1.7%) |
| (Missing) | 0 | 0 | 0 | 1 | 0 | 1 | 0 |
| **That the AI will lead to me getting inappropriate treatment for my mental health** |  |  |  |  |  |  |  |
| Very concerned | 23 (45%) | 67 (43%) | 55 (42%) | 73 (48%) | 5 (50%) | 126 (51%) | 91 (38%) |
| Somewhat concerned | 22 (43%) | 73 (47%) | 57 (43%) | 56 (37%) | 4 (40%) | 90 (36%) | 116 (49%) |
| Not concerned | 6 (12%) | 15 (9.7%) | 18 (14%) | 19 (13%) | 1 (10%) | 31 (12%) | 27 (11%) |
| Don't know | 0 (0%) | 0 (0%) | 2 (1.5%) | 3 (2.0%) | 0 (0%) | 2 (0.8%) | 3 (1.3%) |
| (Missing) | 0 | 0 | 0 | 1 | 0 | 0 | 1 |
| **That AI will mean I spend less time with my mental health professional** |  |  |  |  |  |  |  |
| Very concerned | 22 (43%) | 50 (32%) | 44 (33%) | 58 (38%) | 2 (20%) | 103 (41%) | 68 (29%) |
| Somewhat concerned | 19 (37%) | 54 (35%) | 39 (30%) | 53 (35%) | 5 (50%) | 75 (30%) | 92 (39%) |
| Not concerned | 8 (16%) | 41 (26%) | 45 (34%) | 35 (23%) | 3 (30%) | 61 (24%) | 67 (28%) |
| Don't know | 2 (3.9%) | 10 (6.5%) | 4 (3.0%) | 6 (3.9%) | 0 (0%) | 10 (4.0%) | 11 (4.6%) |
| **That AI will lead to my mental health provider not knowing me as well^†^** |  |  |  |  |  |  |  |
| Very concerned | 23 (46%) | 59 (38%) | 54 (41%) | 69 (45%) | 4 (40%) | 124 (50%) | 82 (35%) |
| Somewhat concerned | 18 (36%) | 69 (45%) | 51 (39%) | 60 (39%) | 2 (20%) | 87 (35%) | 107 (45%) |
| Not concerned | 8 (16%) | 24 (15%) | 23 (17%) | 21 (14%) | 4 (40%) | 34 (14%) | 42 (18%) |
| Don't know | 1 (2.0%) | 3 (1.9%) | 4 (3.0%) | 2 (1.3%) | 0 (0%) | 4 (1.6%) | 6 (2.5%) |
| (Missing) | 1 | 0 | 0 | 0 | 0 | 0 | 1 |
| **That AI will increase my mental health care costs** |  |  |  |  |  |  |  |
| Very concerned | 6 (12%) | 27 (17%) | 27 (20%) | 24 (16%) | 1 (10%) | 46 (19%) | 37 (16%) |
| Somewhat concerned | 20 (39%) | 44 (28%) | 32 (24%) | 34 (23%) | 1 (10%) | 62 (25%) | 60 (25%) |
| Not concerned | 23 (45%) | 70 (45%) | 63 (48%) | 75 (50%) | 7 (70%) | 119 (48%) | 117 (49%) |
| Don't know | 2 (3.9%) | 14 (9.0%) | 10 (7.6%) | 18 (12%) | 1 (10%) | 21 (8.5%) | 24 (10%) |
| (Missing) | 0 | 0 | 0 | 1 | 0 | 1 | 0 |
| **Explainability and transparency** |  |  |  |  |  |  |  |
| **Importance of being told when AI played a big role in mental health diagnosis or treatment** |  |  |  |  |  |  |  |
| Not important | 3 (5.9%) | 5 (3.2%) | 3 (2.3%) | 2 (1.3%) | 0 (0%) | 6 (2.4%) | 6 (2.5%) |
| Somewhat important | 14 (27%) | 41 (26%) | 28 (21%) | 28 (18%) | 0 (0%) | 44 (18%) | 64 (27%) |
| Very important | 33 (65%) | 107 (69%) | 97 (73%) | 117 (77%) | 10 (100%) | 192 (77%) | 164 (69%) |
| Don't know | 1 (2.0%) | 2 (1.3%) | 4 (3.0%) | 5 (3.3%) | 0 (0%) | 7 (2.8%) | 4 (1.7%) |
| **Importance of being told when AI played a small role in mental health diagnosis or treatment** |  |  |  |  |  |  |  |
| Not important | 5 (9.8%) | 16 (10%) | 8 (6.1%) | 10 (6.6%) | 0 (0%) | 13 (5.2%) | 23 (9.7%) |
| Somewhat important | 23 (45%) | 62 (40%) | 49 (37%) | 62 (41%) | 2 (20%) | 99 (40%) | 94 (39%) |
| Very important | 22 (43%) | 74 (48%) | 72 (55%) | 77 (51%) | 8 (80%) | 130 (52%) | 118 (50%) |
| Don't know | 1 (2.0%) | 3 (1.9%) | 3 (2.3%) | 3 (2.0%) | 0 (0%) | 7 (2.8%) | 3 (1.3%) |
| **[Given scenario where AI recommends anti-depressant] Importance of being told that AI helped make this decision** |  |  |  |  |  |  |  |
| Not important | 4 (7.8%) | 8 (5.2%) | 6 (4.5%) | 8 (5.3%) | 0 (0%) | 13 (5.2%) | 12 (5.0%) |
| Somewhat important | 13 (25%) | 31 (20%) | 29 (22%) | 38 (25%) | 2 (20%) | 57 (23%) | 53 (22%) |
| Very important | 34 (67%) | 114 (74%) | 95 (72%) | 104 (68%) | 8 (80%) | 176 (71%) | 170 (71%) |
| Don't know | 0 (0%) | 2 (1.3%) | 2 (1.5%) | 2 (1.3%) | 0 (0%) | 3 (1.2%) | 3 (1.3%) |
| **Comfort receiving a diagnosis from AI that is 90% accurate but unexplainable** |  |  |  |  |  |  |  |
| Very comfortable | 3 (5.9%) | 5 (3.2%) | 6 (4.5%) | 0 (0%) | 1 (10%) | 6 (2.4%) | 7 (2.9%) |
| Somewhat comfortable | 11 (22%) | 29 (19%) | 28 (21%) | 24 (16%) | 2 (20%) | 43 (17%) | 48 (20%) |
| Somewhat uncomfortable | 14 (27%) | 60 (39%) | 43 (33%) | 63 (41%) | 4 (40%) | 92 (37%) | 90 (38%) |
| Very uncomfortable | 22 (43%) | 59 (38%) | 51 (39%) | 64 (42%) | 3 (30%) | 103 (41%) | 90 (38%) |
| Don't know | 1 (2.0%) | 2 (1.3%) | 4 (3.0%) | 1 (0.7%) | 0 (0%) | 5 (2.0%) | 3 (1.3%) |
| **Comfort receiving a diagnosis from AI that is 98% accurate but unexplainable** |  |  |  |  |  |  |  |
| Very comfortable | 7 (14%) | 18 (12%) | 17 (13%) | 19 (12%) | 2 (20%) | 27 (11%) | 32 (13%) |
| Somewhat comfortable | 19 (37%) | 46 (30%) | 42 (32%) | 28 (18%) | 3 (30%) | 66 (27%) | 68 (29%) |
| Somewhat uncomfortable | 15 (29%) | 53 (34%) | 39 (30%) | 63 (41%) | 2 (20%) | 81 (33%) | 90 (38%) |
| Very uncomfortable | 8 (16%) | 34 (22%) | 32 (24%) | 40 (26%) | 3 (30%) | 68 (27%) | 45 (19%) |
| Don't know | 2 (3.9%) | 4 (2.6%) | 2 (1.5%) | 2 (1.3%) | 0 (0%) | 7 (2.8%) | 3 (1.3%) |
| **Trust and responsibility** |  |  |  |  |  |  |  |
| **[Given scenario where AI and doctor opinion conflicts] How does the computer program affect your view?** |  |  |  |  |  |  |  |
| It would not affect my trust of the mental health  professional’s assessment | 9 (18%) | 22 (14%) | 21 (16%) | 30 (20%) | 3 (30%) | 36 (14%) | 47 (20%) |
| It would make me question the mental health  professional’s assessment | 33 (65%) | 82 (53%) | 72 (55%) | 74 (49%) | 4 (40%) | 133 (53%) | 123 (52%) |
| I do not know if it would change my view of the  mental health professional’s assessment | 9 (18%) | 49 (32%) | 35 (27%) | 41 (27%) | 3 (30%) | 74 (30%) | 61 (26%) |
| Don't know | 0 (0%) | 2 (1.3%) | 4 (3.0%) | 7 (4.6%) | 0 (0%) | 6 (2.4%) | 7 (2.9%) |
| **Responsibility for medical errors resulting from AI (select all that apply)** |  |  |  |  |  |  |  |
| Mental health professional | 42 (82%) | 118 (76%) | 111 (84%) | 132 (87%) | 9 (90%) | 209 (84%) | 196 (82%) |
| Company that made the computer program | 21 (41%) | 56 (36%) | 47 (36%) | 55 (36%) | 4 (40%) | 84 (34%) | 92 (39%) |
| Hospital or clinic that bought the computer program* | 18 (35%) | 50 (32%) | 39 (30%) | 44 (29%) | 2 (20%) | 71 (29%) | 79 (33%) |
| Government agency that approved the computer  program* | 17 (33%) | 37 (24%) | 23 (17%) | 36 (24%) | 3 (30%) | 54 (22%) | 60 (25%) |
| Someone else | 1 (2.0%) | 6 (3.9%) | 4 (3.0%) | 6 (3.9%) | 0 (0%) | 9 (3.6%) | 6 (2.5%) |
| No one | 0 (0%) | 1 (0.6%) | 0 (0%) | 0 (0%) | 0 (0%) | 1 (0.4%) | 0 (0%) |
| Don't know | 2 (3.9%) | 12 (7.7%) | 8 (6.1%) | 5 (3.3%) | 0 (0%) | 16 (6.4%) | 10 (4.2%) |
| **Blame for medical errors resulting from AI (select all that apply)** |  |  |  |  |  |  |  |
| Mental health professional | 40 (78%) | 124 (80%) | 111 (84%) | 126 (83%) | 7 (70%) | 209 (84%) | 192 (81%) |
| Company that made the computer program | 17 (33%) | 58 (37%) | 39 (30%) | 51 (34%) | 4 (40%) | 75 (30%) | 88 (37%) |
| Hospital or clinic that bought the computer program* | 12 (24%) | 43 (28%) | 32 (24%) | 36 (24%) | 2 (20%) | 56 (22%) | 67 (28%) |
| Government agency that approved the computer  program | 15 (29%) | 36 (23%) | 19 (14%) | 33 (22%) | 2 (20%) | 48 (19%) | 55 (23%) |
| Someone else | 1 (2.0%) | 2 (1.3%) | 4 (3.0%) | 3 (2.0%) | 0 (0%) | 4 (1.6%) | 4 (1.7%) |
| No one | 3 (5.9%) | 4 (2.6%) | 6 (4.5%) | 5 (3.3%) | 1 (10%) | 8 (3.2%) | 10 (4.2%) |
| Don't know | 1 (2.0%) | 7 (4.5%) | 2 (1.5%) | 5 (3.3%) | 0 (0%) | 8 (3.2%) | 5 (2.1%) |
| **Responsibility for checking that AI is safe (select all that apply)** |  |  |  |  |  |  |  |
| Mental health professional | 13 (25%) | 40 (26%) | 39 (30%) | 40 (26%) | 3 (30%) | 59 (24%) | 72 (30%) |
| Company that made the computer program | 37 (73%) | 105 (68%) | 83 (63%) | 96 (63%) | 7 (70%) | 161 (65%) | 155 (65%) |
| Hospital or clinic that bought the computer program* | 31 (61%) | 90 (58%) | 73 (55%) | 80 (53%) | 8 (80%) | 142 (57%) | 132 (55%) |
| Government agency that approved the computer  program* | 28 (55%) | 85 (55%) | 56 (42%) | 65 (43%) | 4 (40%) | 116 (47%) | 117 (49%) |
| Someone else | 1 (2.0%) | 2 (1.3%) | 1 (0.8%) | 3 (2.0%) | 0 (0%) | 3 (1.2%) | 3 (1.3%) |
| No one* | 0 (0%) | 3 (1.9%) | 8 (6.1%) | 7 (4.6%) | 0 (0%) | 9 (3.6%) | 9 (3.8%) |
| Don't know | 1 (2.0%) | 2 (1.3%) | 4 (3.0%) | 6 (3.9%) | 0 (0%) | 6 (2.4%) | 7 (2.9%) |
| **Importance of bioethical constructs** |  |  |  |  |  |  |  |
| **How important in general: That people are able to make up their own mind about their risk for depression based on AI output [autonomy]** |  |  |  |  |  |  |  |
| Very important | 27 (53%) | 89 (57%) | 73 (55%) | 83 (55%) | 8 (80%) | 143 (57%) | 128 (54%) |
| Somewhat important | 20 (39%) | 54 (35%) | 48 (36%) | 55 (36%) | 1 (10%) | 84 (34%) | 91 (38%) |
| Not important | 1 (2.0%) | 7 (4.5%) | 4 (3.0%) | 9 (5.9%) | 1 (10%) | 10 (4.0%) | 12 (5.0%) |
| Don't know | 3 (5.9%) | 5 (3.2%) | 7 (5.3%) | 5 (3.3%) | 0 (0%) | 12 (4.8%) | 7 (2.9%) |
| **How important in general: That AI will improve depressive symptoms [beneficence]** |  |  |  |  |  |  |  |
| Very important | 20 (39%) | 76 (49%) | 61 (46%) | 67 (44%) | 3 (30%) | 108 (43%) | 113 (47%) |
| Somewhat important | 24 (47%) | 60 (39%) | 49 (37%) | 61 (40%) | 5 (50%) | 101 (41%) | 94 (39%) |
| Not important | 5 (9.8%) | 9 (5.8%) | 12 (9.1%) | 11 (7.2%) | 1 (10%) | 18 (7.2%) | 20 (8.4%) |
| Don't know | 2 (3.9%) | 10 (6.5%) | 10 (7.6%) | 13 (8.6%) | 1 (10%) | 22 (8.8%) | 11 (4.6%) |
| **How important in general: That AI will reduce the chance of negative outcomes [non-maleficence]** |  |  |  |  |  |  |  |
| Very important | 37 (73%) | 96 (62%) | 94 (71%) | 103 (68%) | 7 (70%) | 182 (73%) | 145 (61%) |
| Somewhat important | 14 (27%) | 50 (32%) | 31 (23%) | 40 (26%) | 2 (20%) | 56 (22%) | 79 (33%) |
| Not important | 0 (0%) | 6 (3.9%) | 4 (3.0%) | 6 (3.9%) | 0 (0%) | 7 (2.8%) | 9 (3.8%) |
| Don't know | 0 (0%) | 3 (1.9%) | 3 (2.3%) | 3 (2.0%) | 1 (10%) | 4 (1.6%) | 5 (2.1%) |
| **How important in general: That people can understand how likely it is that they develop depression in the next year according to the AI [justice]** |  |  |  |  |  |  |  |
| Very important | 28 (55%) | 103 (66%) | 99 (75%) | 92 (61%) | 6 (60%) | 166 (67%) | 155 (65%) |
| Somewhat important | 18 (35%) | 41 (26%) | 27 (20%) | 45 (30%) | 3 (30%) | 65 (26%) | 66 (28%) |
| Not important | 2 (3.9%) | 4 (2.6%) | 5 (3.8%) | 4 (2.6%) | 0 (0%) | 5 (2.0%) | 10 (4.2%) |
| Don't know | 3 (5.9%) | 7 (4.5%) | 1 (0.8%) | 11 (7.2%) | 1 (10%) | 13 (5.2%) | 7 (2.9%) |
| **How important in general: That AI does not reduce people’s trust in their mental health care professionals [trust]** |  |  |  |  |  |  |  |
| Very important | 15 (29%) | 48 (31%) | 34 (26%) | 51 (34%) | 3 (30%) | 74 (30%) | 71 (30%) |
| Somewhat important | 24 (47%) | 70 (45%) | 73 (55%) | 72 (47%) | 5 (50%) | 124 (50%) | 118 (50%) |
| Not important | 10 (20%) | 28 (18%) | 16 (12%) | 16 (11%) | 2 (20%) | 33 (13%) | 37 (16%) |
| Don't know | 2 (3.9%) | 9 (5.8%) | 9 (6.8%) | 13 (8.6%) | 0 (0%) | 18 (7.2%) | 12 (5.0%) |
| **How important in general: That people are aware of how their personal data is being used for AI [privacy]** |  |  |  |  |  |  |  |
| Very important | 39 (76%) | 109 (70%) | 103 (78%) | 101 (66%) | 10 (100%) | 191 (77%) | 163 (68%) |
| Somewhat important | 10 (20%) | 29 (19%) | 22 (17%) | 31 (20%) | 0 (0%) | 43 (17%) | 45 (19%) |
| Not important | 1 (2.0%) | 13 (8.4%) | 6 (4.5%) | 10 (6.6%) | 0 (0%) | 8 (3.2%) | 22 (9.2%) |
| Don't know | 1 (2.0%) | 4 (2.6%) | 1 (0.8%) | 10 (6.6%) | 0 (0%) | 7 (2.8%) | 8 (3.4%) |
| **How important in general: That people can understand which of their individual risk factors for depression are used by the AI [transparency]** |  |  |  |  |  |  |  |
| Very important | 42 (82%) | 118 (76%) | 113 (86%) | 130 (86%) | 9 (90%) | 220 (88%) | 182 (76%) |
| Somewhat important | 9 (18%) | 28 (18%) | 15 (11%) | 16 (11%) | 0 (0%) | 21 (8.4%) | 45 (19%) |
| Not important | 0 (0%) | 3 (1.9%) | 4 (3.0%) | 2 (1.3%) | 1 (10%) | 3 (1.2%) | 7 (2.9%) |
| Don't know | 0 (0%) | 6 (3.9%) | 0 (0%) | 4 (2.6%) | 0 (0%) | 5 (2.0%) | 4 (1.7%) |
| **How important to you: That you are able to make up your own mind about your risk for depression based on AI output [autonomy]** |  |  |  |  |  |  |  |
| Very important | 31 (61%) | 103 (66%) | 90 (68%) | 93 (61%) | 8 (80%) | 174 (70%) | 141 (59%) |
| Somewhat important | 17 (33%) | 45 (29%) | 34 (26%) | 50 (33%) | 1 (10%) | 60 (24%) | 84 (35%) |
| Not important | 0 (0%) | 5 (3.2%) | 4 (3.0%) | 6 (3.9%) | 1 (10%) | 7 (2.8%) | 9 (3.8%) |
| Don't know | 3 (5.9%) | 2 (1.3%) | 4 (3.0%) | 3 (2.0%) | 0 (0%) | 8 (3.2%) | 4 (1.7%) |
| **How important to you: That AI will improve your depression/ depressive symptoms [beneficence]^‡^** |  |  |  |  |  |  |  |
| Very important | 26 (51%) | 102 (66%) | 81 (61%) | 86 (57%) | 7 (70%) | 154 (62%) | 141 (59%) |
| Somewhat important | 19 (37%) | 43 (28%) | 35 (27%) | 44 (29%) | 3 (30%) | 65 (26%) | 75 (32%) |
| Not important | 4 (7.8%) | 5 (3.2%) | 11 (8.3%) | 13 (8.6%) | 0 (0%) | 17 (6.8%) | 16 (6.7%) |
| Don't know | 2 (3.9%) | 5 (3.2%) | 5 (3.8%) | 9 (5.9%) | 0 (0%) | 13 (5.2%) | 6 (2.5%) |
| **How important to you: That AI will decrease the chance of negative outcomes [non-maleficence]** |  |  |  |  |  |  |  |
| Very important | 39 (76%) | 105 (68%) | 96 (73%) | 108 (71%) | 9 (90%) | 188 (76%) | 159 (67%) |
| Somewhat important | 10 (20%) | 46 (30%) | 30 (23%) | 36 (24%) | 1 (10%) | 53 (21%) | 67 (28%) |
| Not important | 1 (2.0%) | 2 (1.3%) | 4 (3.0%) | 6 (3.9%) | 0 (0%) | 5 (2.0%) | 8 (3.4%) |
| Don't know | 1 (2.0%) | 2 (1.3%) | 2 (1.5%) | 2 (1.3%) | 0 (0%) | 3 (1.2%) | 4 (1.7%) |
| **How important to you: That you can understand how likely it is that you develop depression within the next year according to the AI [justice]** |  |  |  |  |  |  |  |
| Very important | 28 (55%) | 105 (68%) | 101 (77%) | 101 (66%) | 8 (80%) | 177 (71%) | 158 (66%) |
| Somewhat important | 18 (35%) | 40 (26%) | 25 (19%) | 36 (24%) | 2 (20%) | 56 (22%) | 63 (26%) |
| Not important | 2 (3.9%) | 3 (1.9%) | 4 (3.0%) | 6 (3.9%) | 0 (0%) | 5 (2.0%) | 10 (4.2%) |
| Don't know | 3 (5.9%) | 7 (4.5%) | 2 (1.5%) | 9 (5.9%) | 0 (0%) | 11 (4.4%) | 7 (2.9%) |
| **How important to you: That using AI does not reduce your trust in your mental health care provider [trust]** |  |  |  |  |  |  |  |
| Very important | 12 (24%) | 60 (39%) | 53 (40%) | 53 (35%) | 8 (80%) | 89 (36%) | 91 (38%) |
| Somewhat important | 25 (49%) | 60 (39%) | 59 (45%) | 71 (47%) | 0 (0%) | 113 (45%) | 98 (41%) |
| Not important | 10 (20%) | 26 (17%) | 14 (11%) | 15 (9.9%) | 0 (0%) | 27 (11%) | 36 (15%) |
| Don't know | 4 (7.8%) | 9 (5.8%) | 6 (4.5%) | 13 (8.6%) | 2 (20%) | 20 (8.0%) | 13 (5.5%) |
| **How important to you: That you are aware of how your personal data is being used for AI [privacy]** |  |  |  |  |  |  |  |
| Very important | 37 (73%) | 108 (70%) | 94 (71%) | 100 (66%) | 9 (90%) | 185 (74%) | 155 (65%) |
| Somewhat important | 14 (27%) | 33 (21%) | 27 (20%) | 32 (21%) | 1 (10%) | 47 (19%) | 57 (24%) |
| Not important | 0 (0%) | 10 (6.5%) | 10 (7.6%) | 14 (9.2%) | 0 (0%) | 13 (5.2%) | 19 (8.0%) |
| Don't know | 0 (0%) | 4 (2.6%) | 1 (0.8%) | 6 (3.9%) | 0 (0%) | 4 (1.6%) | 7 (2.9%) |
| **How important to you: That you can understand which of your individual risk factors for depression are used by the AI** |  |  |  |  |  |  |  |
| Very important | 37 (73%) | 126 (81%) | 105 (80%) | 126 (83%) | 8 (80%) | 211 (85%) | 182 (76%) |
| Somewhat important | 12 (24%) | 22 (14%) | 21 (16%) | 21 (14%) | 1 (10%) | 32 (13%) | 42 (18%) |
| Not important | 2 (3.9%) | 5 (3.2%) | 5 (3.8%) | 4 (2.6%) | 1 (10%) | 4 (1.6%) | 12 (5.0%) |
| Don't know | 0 (0%) | 2 (1.3%) | 1 (0.8%) | 1 (0.7%) | 0 (0%) | 2 (0.8%) | 2 (0.8%) |
| *Statistical significance represented by grey boxes. Statistical significance determined using Fisher's Exact Test for Count Data with simulated p-value (based on 2000 replicates).* | | | | | | | |

| **Supplementary Table 3:** Differences in perspectives on AI in mental health by demographic characteristics (race and ethnicity) among survey participants (n=500); n(%) | | | | | | |
| --- | --- | --- | --- | --- | --- | --- |
|  | **Race** | | | | **Ethnicity** | |
|  | **Asian**, n=25 | **Black or African American**, n=66 | **White**, n=388 | **Other/ Prefer not to answer**, n=211 | **Hispanic/ Latino**, n=30 | **Not Hispanic/ Latino**, n=464 |
| **General perspectives** |  |  |  |  |  |  |
| **How much do you know about AI and how it could change mental healthcare?** |  |  |  |  |  |  |
| I know quite a lot | 1 (4.0%) | 2 (3.0%) | 4 (1.0%) | 0 (0%) | 0 (0%) | 7 (1.5%) |
| I know a fair amount | 7 (28%) | 18 (27%) | 62 (16%) | 5 (24%) | 9 (30%) | 81 (17%) |
| I know a little bit | 9 (36%) | 31 (47%) | 203 (52%) | 9 (43%) | 15 (50%) | 234 (50%) |
| I know almost nothing | 8 (32%) | 15 (23%) | 119 (31%) | 7 (33%) | 6 (20%) | 142 (31%) |
| **Overall, in the next 5 years, do you think AI will make mental healthcare in the United States?** |  |  |  |  |  |  |
| Much better | 0 (0%) | 8 (12%) | 25 (6.4%) | 1 (4.8%) | 2 (6.7%) | 32 (6.9%) |
| Somewhat better | 15 (60%) | 32 (48%) | 159 (41%) | 7 (33%) | 12 (40%) | 200 (43%) |
| Minimal change | 6 (24%) | 19 (29%) | 137 (35%) | 8 (38%) | 9 (30%) | 158 (34%) |
| Somewhat worse | 1 (4.0%) | 2 (3.0%) | 27 (7.0%) | 4 (19%) | 2 (6.7%) | 31 (6.7%) |
| Much worse | 0 (0%) | 3 (4.5%) | 4 (1.0%) | 1 (4.8%) | 2 (6.7%) | 5 (1.1%) |
| Don't know | 3 (12%) | 2 (3.0%) | 36 (9.3%) | 0 (0%) | 3 (10%) | 38 (8.2%) |
| **Comfort with AI** |  |  |  |  |  |  |
| **AI, instead of a mental health professional, performing a mental health assessment** |  |  |  |  |  |  |
| Very comfortable | 1 (4.0%) | 6 (9.1%) | 36 (9.3%) | 0 (0%) | 2 (6.7%) | 41 (8.8%) |
| Somewhat comfortable | 6 (24%) | 32 (48%) | 144 (37%) | 9 (43%) | 8 (27%) | 183 (39%) |
| Somewhat uncomfortable | 11 (44%) | 16 (24%) | 120 (31%) | 6 (29%) | 12 (40%) | 138 (30%) |
| Very uncomfortable | 6 (24%) | 12 (18%) | 82 (21%) | 6 (29%) | 6 (20%) | 97 (21%) |
| Don't know | 1 (4.0%) | 0 (0%) | 6 (1.5%) | 0 (0%) | 2 (6.7%) | 5 (1.1%) |
| **AI, instead of a mental health professional, making a diagnosis of clinical depression** |  |  |  |  |  |  |
| Very comfortable | 1 (4.0%) | 3 (4.5%) | 21 (5.4%) | 1 (4.8%) | 0 (0%) | 26 (5.6%) |
| Somewhat comfortable | 2 (8.0%) | 21 (32%) | 99 (26%) | 6 (29%) | 7 (23%) | 120 (26%) |
| Somewhat uncomfortable | 15 (60%) | 19 (29%) | 121 (31%) | 3 (14%) | 11 (37%) | 145 (31%) |
| Very uncomfortable | 6 (24%) | 22 (33%) | 140 (36%) | 10 (48%) | 11 (37%) | 164 (35%) |
| Don't know | 1 (4.0%) | 1 (1.5%) | 7 (1.8%) | 1 (4.8%) | 1 (3.3%) | 9 (1.9%) |
| **AI, instead of a mental health professional, telling you that you are clinically depressed** |  |  |  |  |  |  |
| Very comfortable | 1 (4.0%) | 5 (7.6%) | 22 (5.7%) | 1 (4.8%) | 1 (3.3%) | 27 (5.8%) |
| Somewhat comfortable | 5 (20%) | 21 (32%) | 87 (22%) | 6 (29%) | 6 (20%) | 113 (24%) |
| Somewhat uncomfortable | 9 (36%) | 14 (21%) | 118 (30%) | 6 (29%) | 11 (37%) | 135 (29%) |
| Very uncomfortable | 9 (36%) | 25 (38%) | 152 (39%) | 7 (33%) | 12 (40%) | 177 (38%) |
| Don't know | 1 (4.0%) | 1 (1.5%) | 9 (2.3%) | 1 (4.8%) | 0 (0%) | 12 (2.6%) |
| **AI, instead of a mental health professional, making a diagnosis of bi-polar disorder** |  |  |  |  |  |  |
| Very comfortable | 1 (4.0%) | 4 (6.1%) | 20 (5.2%) | 1 (4.8%) | 0 (0%) | 26 (5.6%) |
| Somewhat comfortable | 1 (4.0%) | 16 (24%) | 73 (19%) | 4 (19%) | 7 (23%) | 87 (19%) |
| Somewhat uncomfortable | 14 (56%) | 17 (26%) | 102 (26%) | 4 (19%) | 11 (37%) | 125 (27%) |
| Very uncomfortable | 8 (32%) | 26 (39%) | 184 (47%) | 11 (52%) | 11 (37%) | 213 (46%) |
| Don't know | 1 (4.0%) | 3 (4.5%) | 9 (2.3%) | 1 (4.8%) | 1 (3.3%) | 13 (2.8%) |
| **AI, instead of a mental health professional, telling you that you have bi-polar disorder** |  |  |  |  |  |  |
| Very comfortable | 1 (4.0%) | 3 (4.5%) | 18 (4.6%) | 1 (4.8%) | 0 (0%) | 22 (4.7%) |
| Somewhat comfortable | 2 (8.0%) | 14 (21%) | 58 (15%) | 3 (14%) | 4 (13%) | 73 (16%) |
| Somewhat uncomfortable | 12 (48%) | 18 (27%) | 118 (30%) | 5 (24%) | 13 (43%) | 140 (30%) |
| Very uncomfortable | 9 (36%) | 29 (44%) | 184 (47%) | 11 (52%) | 12 (40%) | 216 (47%) |
| Don't know | 1 (4.0%) | 2 (3.0%) | 10 (2.6%) | 1 (4.8%) | 1 (3.3%) | 13 (2.8%) |
| **AI, instead of a mental health professional, recommending a general wellness or stress-management strategy** |  |  |  |  |  |  |
| Very comfortable | 4 (16%) | 22 (33%) | 105 (27%) | 3 (14%) | 8 (27%) | 126 (27%) |
| Somewhat comfortable | 12 (48%) | 31 (47%) | 171 (44%) | 10 (48%) | 13 (43%) | 210 (45%) |
| Somewhat uncomfortable | 2 (8.0%) | 10 (15%) | 60 (15%) | 3 (14%) | 5 (17%) | 68 (15%) |
| Very uncomfortable | 5 (20%) | 3 (4.5%) | 45 (12%) | 4 (19%) | 3 (10%) | 51 (11%) |
| Don't know | 2 (8.0%) | 0 (0%) | 7 (1.8%) | 1 (4.8%) | 1 (3.3%) | 9 (1.9%) |
| **AI, instead of a mental health professional, recommending a talk therapy** |  |  |  |  |  |  |
| Very comfortable | 3 (12%) | 17 (26%) | 92 (24%) | 3 (14%) | 6 (20%) | 108 (23%) |
| Somewhat comfortable | 12 (48%) | 32 (48%) | 161 (41%) | 7 (33%) | 14 (47%) | 198 (43%) |
| Somewhat uncomfortable | 4 (16%) | 10 (15%) | 70 (18%) | 4 (19%) | 5 (17%) | 82 (18%) |
| Very uncomfortable | 5 (20%) | 7 (11%) | 58 (15%) | 5 (24%) | 5 (17%) | 66 (14%) |
| Don't know | 1 (4.0%) | 0 (0%) | 7 (1.8%) | 2 (9.5%) | 0 (0%) | 10 (2.2%) |
| **AI, instead of a mental health professional, recommending a medication** |  |  |  |  |  |  |
| Very comfortable | 1 (4.0%) | 5 (7.6%) | 26 (6.7%) | 1 (4.8%) | 1 (3.3%) | 32 (6.9%) |
| Somewhat comfortable | 5 (20%) | 15 (23%) | 78 (20%) | 5 (24%) | 4 (13%) | 99 (21%) |
| Somewhat uncomfortable | 5 (20%) | 18 (27%) | 120 (31%) | 6 (29%) | 10 (33%) | 137 (30%) |
| Very uncomfortable | 12 (48%) | 28 (42%) | 158 (41%) | 9 (43%) | 14 (47%) | 189 (41%) |
| Don't know | 2 (8.0%) | 0 (0%) | 6 (1.5%) | 0 (0%) | 1 (3.3%) | 7 (1.5%) |
| **AI, instead of a mental health professional, predicting a patient’s risk for suicide** |  |  |  |  |  |  |
| Very comfortable | 1 (4.0%) | 7 (11%) | 30 (7.7%) | 1 (4.8%) | 1 (3.3%) | 37 (8.0%) |
| Somewhat comfortable | 3 (12%) | 17 (26%) | 77 (20%) | 3 (14%) | 4 (13%) | 95 (20%) |
| Somewhat uncomfortable | 9 (36%) | 18 (27%) | 104 (27%) | 6 (29%) | 6 (20%) | 130 (28%) |
| Very uncomfortable | 10 (40%) | 22 (33%) | 156 (40%) | 9 (43%) | 18 (60%) | 176 (38%) |
| Don't know | 2 (8.0%) | 2 (3.0%) | 21 (5.4%) | 2 (9.5%) | 1 (3.3%) | 26 (5.6%) |
| **AI, instead of a mental health professional, predicting a patient’s risk of engaging in violent behavior** |  |  |  |  |  |  |
| Very comfortable | 1 (4.0%) | 4 (6.1%) | 32 (8.2%) | 1 (4.8%) | 2 (6.7%) | 36 (7.8%) |
| Somewhat comfortable | 3 (12%) | 20 (30%) | 85 (22%) | 4 (19%) | 6 (20%) | 104 (22%) |
| Somewhat uncomfortable | 8 (32%) | 18 (27%) | 96 (25%) | 5 (24%) | 7 (23%) | 119 (26%) |
| Very uncomfortable | 11 (44%) | 22 (33%) | 154 (40%) | 9 (43%) | 14 (47%) | 179 (39%) |
| Don't know | 2 (8.0%) | 2 (3.0%) | 21 (5.4%) | 2 (9.5%) | 1 (3.3%) | 26 (5.6%) |
| **Sharing sensitive information with a human mental health professional** |  |  |  |  |  |  |
| Very comfortable | 12 (48%) | 27 (41%) | 137 (35%) | 5 (24%) | 11 (37%) | 168 (36%) |
| Somewhat comfortable | 6 (24%) | 24 (36%) | 169 (44%) | 9 (43%) | 10 (33%) | 197 (42%) |
| Somewhat uncomfortable | 4 (16%) | 11 (17%) | 62 (16%) | 5 (24%) | 6 (20%) | 74 (16%) |
| Very uncomfortable | 3 (12%) | 4 (6.1%) | 19 (4.9%) | 2 (9.5%) | 2 (6.7%) | 25 (5.4%) |
| Don't know | 0 (0%) | 0 (0%) | 1 (0.3%) | 0 (0%) | 1 (3.3%) | 0 (0%) |
| **Sharing sensitive information with an AI chatbot** |  |  |  |  |  |  |
| Very comfortable | 2 (8.0%) | 11 (17%) | 56 (14%) | 3 (14%) | 4 (13%) | 68 (15%) |
| Somewhat comfortable | 5 (20%) | 30 (45%) | 120 (31%) | 10 (48%) | 8 (27%) | 156 (34%) |
| Somewhat uncomfortable | 8 (32%) | 10 (15%) | 109 (28%) | 3 (14%) | 11 (37%) | 118 (25%) |
| Very uncomfortable | 10 (40%) | 15 (23%) | 95 (24%) | 5 (24%) | 6 (20%) | 116 (25%) |
| Don't know | 0 (0%) | 0 (0%) | 8 (2.1%) | 0 (0%) | 1 (3.3%) | 6 (1.3%) |
| **Sharing sensitive information to help improve AI programs that treat disease** |  |  |  |  |  |  |
| Very comfortable | 5 (20%) | 10 (15%) | 90 (23%) | 7 (33%) | 8 (27%) | 104 (22%) |
| Somewhat comfortable | 10 (40%) | 36 (55%) | 135 (35%) | 7 (33%) | 9 (30%) | 177 (38%) |
| Somewhat uncomfortable | 4 (16%) | 9 (14%) | 85 (22%) | 2 (9.5%) | 4 (13%) | 94 (20%) |
| Very uncomfortable | 4 (16%) | 8 (12%) | 58 (15%) | 4 (19%) | 6 (20%) | 67 (14%) |
| Don't know | 2 (8.0%) | 3 (4.5%) | 20 (5.2%) | 1 (4.8%) | 3 (10%) | 22 (4.7%) |
| **Specific concerns** |  |  |  |  |  |  |
| **That my mental health information will not be kept confidential** |  |  |  |  |  |  |
| Very concerned | 9 (36%) | 14 (21%) | 81 (21%) | 8 (38%) | 4 (13%) | 104 (22%) |
| Somewhat concerned | 9 (36%) | 28 (42%) | 146 (38%) | 7 (33%) | 13 (43%) | 176 (38%) |
| Not concerned | 7 (28%) | 23 (35%) | 157 (40%) | 6 (29%) | 12 (40%) | 180 (39%) |
| Don't know | 0 (0%) | 1 (1.5%) | 4 (1.0%) | 0 (0%) | 1 (3.3%) | 4 (0.9%) |
| **That the AI will make the wrong diagnosis about my mental health** |  |  |  |  |  |  |
| Very concerned | 16 (64%) | 34 (52%) | 163 (42%) | 8 (38%) | 15 (50%) | 203 (44%) |
| Somewhat concerned | 7 (28%) | 24 (36%) | 187 (48%) | 10 (48%) | 14 (47%) | 212 (46%) |
| Not concerned | 1 (4.0%) | 7 (11%) | 36 (9.3%) | 2 (9.5%) | 1 (3.3%) | 44 (9.5%) |
| Don't know | 1 (4.0%) | 1 (1.5%) | 1 (0.3%) | 1 (4.8%) | 0 (0%) | 4 (0.9%) |
| (Missing) | 0 | 0 | 1 | 0 | 0 | 1 |
| **That the AI will lead to me getting inappropriate treatment for my mental health** |  |  |  |  |  |  |
| Very concerned | 16 (64%) | 30 (45%) | 170 (44%) | 7 (33%) | 16 (53%) | 204 (44%) |
| Somewhat concerned | 7 (28%) | 29 (44%) | 166 (43%) | 10 (48%) | 12 (40%) | 197 (43%) |
| Not concerned | 1 (4.0%) | 6 (9.1%) | 48 (12%) | 4 (19%) | 2 (6.7%) | 57 (12%) |
| Don't know | 1 (4.0%) | 1 (1.5%) | 3 (0.8%) | 0 (0%) | 0 (0%) | 5 (1.1%) |
| (Missing) | 0 | 0 | 1 | 0 | 0 | 1 |
| **That AI will mean I spend less time with my mental health professional** |  |  |  |  |  |  |
| Very concerned | 9 (36%) | 22 (33%) | 141 (36%) | 4 (19%) | 14 (47%) | 161 (35%) |
| Somewhat concerned | 10 (40%) | 23 (35%) | 131 (34%) | 6 (29%) | 8 (27%) | 159 (34%) |
| Not concerned | 5 (20%) | 13 (20%) | 106 (27%) | 8 (38%) | 8 (27%) | 122 (26%) |
| Don't know | 1 (4.0%) | 8 (12%) | 10 (2.6%) | 3 (14%) | 0 (0%) | 22 (4.7%) |
| **That AI will lead to my mental health provider not knowing me as well** |  |  |  |  |  |  |
| Very concerned | 8 (32%) | 26 (39%) | 169 (44%) | 6 (29%) | 18 (60%) | 189 (41%) |
| Somewhat concerned | 13 (52%) | 28 (42%) | 149 (39%) | 10 (48%) | 7 (23%) | 190 (41%) |
| Not concerned | 4 (16%) | 9 (14%) | 64 (17%) | 3 (14%) | 4 (13%) | 75 (16%) |
| Don't know | 0 (0%) | 3 (4.5%) | 5 (1.3%) | 2 (9.5%) | 1 (3.3%) | 9 (1.9%) |
| (Missing) | 0 | 0 | 1 | 0 | 0 | 1 |
| **That AI will increase my mental health care costs** |  |  |  |  |  |  |
| Very concerned | 5 (20%) | 18 (27%) | 57 (15%) | 5 (24%) | 8 (27%) | 75 (16%) |
| Somewhat concerned | 6 (24%) | 17 (26%) | 101 (26%) | 7 (33%) | 7 (23%) | 122 (26%) |
| Not concerned | 10 (40%) | 26 (39%) | 195 (50%) | 7 (33%) | 13 (43%) | 225 (49%) |
| Don't know | 4 (16%) | 5 (7.6%) | 34 (8.8%) | 2 (9.5%) | 2 (6.7%) | 41 (8.9%) |
| (Missing) | 0 | 0 | 1 | 0 | 0 | 1 |
| **Explainability and transparency** |  |  |  |  |  |  |
| **Importance of being told when AI played a big role in mental health diagnosis or treatment** |  |  |  |  |  |  |
| Not important | 2 (8.0%) | 0 (0%) | 11 (2.8%) | 0 (0%) | 1 (3.3%) | 12 (2.6%) |
| Somewhat important | 5 (20%) | 14 (21%) | 87 (22%) | 5 (24%) | 7 (23%) | 104 (22%) |
| Very important | 17 (68%) | 50 (76%) | 283 (73%) | 14 (67%) | 21 (70%) | 338 (73%) |
| Don't know | 1 (4.0%) | 2 (3.0%) | 7 (1.8%) | 2 (9.5%) | 1 (3.3%) | 10 (2.2%) |
| **Importance of being told when AI played a small role in mental health diagnosis or treatment** |  |  |  |  |  |  |
| Not important | 3 (12%) | 6 (9.1%) | 29 (7.5%) | 1 (4.8%) | 1 (3.3%) | 37 (8.0%) |
| Somewhat important | 12 (48%) | 26 (39%) | 153 (39%) | 7 (33%) | 11 (37%) | 186 (40%) |
| Very important | 10 (40%) | 33 (50%) | 198 (51%) | 12 (57%) | 17 (57%) | 232 (50%) |
| Don't know | 0 (0%) | 1 (1.5%) | 8 (2.1%) | 1 (4.8%) | 1 (3.3%) | 9 (1.9%) |
| **[Given scenario where AI recommends anti-depressant] Importance of being told that AI helped make this decision** |  |  |  |  |  |  |
| Not important | 1 (4.0%) | 5 (7.6%) | 20 (5.2%) | 0 (0%) | 0 (0%) | 26 (5.6%) |
| Somewhat important | 7 (28%) | 10 (15%) | 92 (24%) | 4 (19%) | 8 (27%) | 105 (23%) |
| Very important | 17 (68%) | 49 (74%) | 273 (70%) | 16 (76%) | 21 (70%) | 328 (71%) |
| Don't know | 0 (0%) | 2 (3.0%) | 3 (0.8%) | 1 (4.8%) | 1 (3.3%) | 5 (1.1%) |
| **Comfort receiving a diagnosis from AI that is 90% accurate but unexplainable** |  |  |  |  |  |  |
| Very comfortable | 2 (8.0%) | 1 (1.5%) | 12 (3.1%) | 0 (0%) | 0 (0%) | 14 (3.0%) |
| Somewhat comfortable | 6 (24%) | 12 (18%) | 72 (19%) | 4 (19%) | 5 (17%) | 89 (19%) |
| Somewhat uncomfortable | 6 (24%) | 26 (39%) | 147 (38%) | 5 (24%) | 11 (37%) | 173 (37%) |
| Very uncomfortable | 11 (44%) | 27 (41%) | 151 (39%) | 10 (48%) | 12 (40%) | 182 (39%) |
| Don't know | 0 (0%) | 0 (0%) | 6 (1.5%) | 2 (9.5%) | 2 (6.7%) | 6 (1.3%) |
| **Comfort receiving a diagnosis from AI that is 98% accurate but unexplainable** |  |  |  |  |  |  |
| Very comfortable | 5 (20%) | 8 (12%) | 47 (12%) | 3 (14%) | 6 (20%) | 57 (12%) |
| Somewhat comfortable | 7 (28%) | 18 (27%) | 109 (28%) | 4 (19%) | 5 (17%) | 132 (28%) |
| Somewhat uncomfortable | 7 (28%) | 21 (32%) | 139 (36%) | 5 (24%) | 8 (27%) | 162 (35%) |
| Very uncomfortable | 6 (24%) | 19 (29%) | 84 (22%) | 8 (38%) | 8 (27%) | 106 (23%) |
| Don't know | 0 (0%) | 0 (0%) | 9 (2.3%) | 1 (4.8%) | 3 (10%) | 7 (1.5%) |
| **Trust and responsibility** |  |  |  |  |  |  |
| **[Given scenario where AI and doctor opinion conflicts] How does the computer program affect your view?** |  |  |  |  |  |  |
| It would not affect my trust of the mental health professional’s assessment | 7 (28%) | 10 (15%) | 67 (17%) | 1 (4.8%) | 5 (17%) | 79 (17%) |
| It would make me question the mental health professional’s assessment | 15 (60%) | 38 (58%) | 200 (52%) | 12 (57%) | 16 (53%) | 246 (53%) |
| I do not know if it would change my view of the mental health professional’s  assessment | 2 (8.0%) | 18 (27%) | 111 (29%) | 6 (29%) | 9 (30%) | 126 (27%) |
| Don't know | 1 (4.0%) | 0 (0%) | 10 (2.6%) | 2 (9.5%) | 0 (0%) | 13 (2.8%) |
| **Responsibility for medical errors resulting from AI (select all that apply)** |  |  |  |  |  |  |
| Mental health professional | 22 (88%) | 56 (85%) | 320 (82%) | 14 (67%) | 21 (70%) | 388 (84%) |
| Company that made the computer program | 12 (48%) | 26 (39%) | 134 (35%) | 11 (52%) | 10 (33%) | 170 (37%) |
| Hospital or clinic that bought the computer program | 11 (44%) | 27 (41%) | 108 (28%) | 7 (33%) | 9 (30%) | 142 (31%) |
| Government agency that approved the computer program | 7 (28%) | 22 (33%) | 80 (21%) | 7 (33%) | 6 (20%) | 109 (23%) |
| Someone else | 0 (0%) | 4 (6.1%) | 11 (2.8%) | 2 (9.5%) | 1 (3.3%) | 14 (3.0%) |
| No one | 0 (0%) | 0 (0%) | 1 (0.3%) | 0 (0%) | 1 (3.3%) | 0 (0%) |
| Don't know | 2 (8.0%) | 1 (1.5%) | 22 (5.7%) | 2 (9.5%) | 1 (3.3%) | 26 (5.6%) |
| **Blame for medical errors resulting from AI (select all that apply)** |  |  |  |  |  |  |
| Mental health professional | 22 (88%) | 58 (88%) | 312 (80%) | 16 (76%) | 23 (77%) | 380 (82%) |
| Company that made the computer program | 13 (52%) | 24 (36%) | 124 (32%) | 8 (38%) | 6 (20%) | 161 (35%) |
| Hospital or clinic that bought the computer program | 12 (48%) | 21 (32%) | 85 (22%) | 7 (33%) | 9 (30%) | 114 (25%) |
| Government agency that approved the computer program | 9 (36%) | 19 (29%) | 71 (18%) | 6 (29%) | 5 (17%) | 99 (21%) |
| Someone else | 0 (0%) | 3 (4.5%) | 6 (1.5%) | 1 (4.8%) | 1 (3.3%) | 7 (1.5%) |
| No one | 0 (0%) | 0 (0%) | 18 (4.6%) | 1 (4.8%) | 1 (3.3%) | 18 (3.9%) |
| Don't know | 0 (0%) | 0 (0%) | 14 (3.6%) | 1 (4.8%) | 1 (3.3%) | 13 (2.8%) |
| **Responsibility for checking that AI is safe (select all that apply)** |  |  |  |  |  |  |
| Mental health professional | 14 (56%) | 17 (26%) | 99 (26%) | 5 (24%) | 8 (27%) | 126 (27%) |
| Company that made the computer program | 18 (72%) | 45 (68%) | 251 (65%) | 14 (67%) | 20 (67%) | 306 (66%) |
| Hospital or clinic that bought the computer program | 18 (72%) | 40 (61%) | 212 (55%) | 12 (57%) | 15 (50%) | 266 (57%) |
| Government agency that approved the computer program | 12 (48%) | 36 (55%) | 180 (46%) | 10 (48%) | 13 (43%) | 222 (48%) |
| Someone else | 3 (12%) | 0 (0%) | 4 (1.0%) | 0 (0%) | 0 (0%) | 7 (1.5%) |
| No one | 1 (4.0%) | 1 (1.5%) | 15 (3.9%) | 1 (4.8%) | 1 (3.3%) | 16 (3.4%) |
| Don't know | 1 (4.0%) | 0 (0%) | 11 (2.8%) | 1 (4.8%) | 1 (3.3%) | 11 (2.4%) |
| **Importance of bioethical constructs** |  |  |  |  |  |  |
| **How important in general: That people are able to make up their own mind about their risk for depression based on AI output [autonomy]** |  |  |  |  |  |  |
| Very important | 13 (52%) | 38 (58%) | 219 (56%) | 10 (48%) | 19 (63%) | 257 (55%) |
| Somewhat important | 9 (36%) | 26 (39%) | 135 (35%) | 8 (38%) | 8 (27%) | 170 (37%) |
| Not important | 3 (12%) | 0 (0%) | 18 (4.6%) | 1 (4.8%) | 0 (0%) | 22 (4.7%) |
| Don't know | 0 (0%) | 2 (3.0%) | 16 (4.1%) | 2 (9.5%) | 3 (10%) | 15 (3.2%) |
| **How important in general: That AI will improve depressive symptoms [beneficence]** |  |  |  |  |  |  |
| Very important | 14 (56%) | 26 (39%) | 181 (47%) | 6 (29%) | 15 (50%) | 212 (46%) |
| Somewhat important | 6 (24%) | 29 (44%) | 155 (40%) | 9 (43%) | 12 (40%) | 184 (40%) |
| Not important | 3 (12%) | 6 (9.1%) | 27 (7.0%) | 2 (9.5%) | 3 (10%) | 35 (7.5%) |
| Don't know | 2 (8.0%) | 5 (7.6%) | 25 (6.4%) | 4 (19%) | 0 (0%) | 33 (7.1%) |
| **How important in general: That AI will reduce the chance of negative outcomes [non-maleficence]** |  |  |  |  |  |  |
| Very important | 19 (76%) | 42 (64%) | 262 (68%) | 14 (67%) | 22 (73%) | 312 (67%) |
| Somewhat important | 6 (24%) | 22 (33%) | 106 (27%) | 3 (14%) | 8 (27%) | 129 (28%) |
| Not important | 0 (0%) | 2 (3.0%) | 13 (3.4%) | 1 (4.8%) | 0 (0%) | 15 (3.2%) |
| Don't know | 0 (0%) | 0 (0%) | 7 (1.8%) | 3 (14%) | 0 (0%) | 8 (1.7%) |
| **How important in general: That people can understand how likely it is that they develop depression in the next year according to the AI [justice]** |  |  |  |  |  |  |
| Very important | 16 (64%) | 47 (71%) | 254 (65%) | 11 (52%) | 20 (67%) | 308 (66%) |
| Somewhat important | 8 (32%) | 14 (21%) | 106 (27%) | 6 (29%) | 9 (30%) | 121 (26%) |
| Not important | 0 (0%) | 4 (6.1%) | 9 (2.3%) | 2 (9.5%) | 1 (3.3%) | 14 (3.0%) |
| Don't know | 1 (4.0%) | 1 (1.5%) | 19 (4.9%) | 2 (9.5%) | 0 (0%) | 21 (4.5%) |
| **How important in general: That AI does not reduce people’s trust in their mental health care professionals [trust]** |  |  |  |  |  |  |
| Very important | 5 (20%) | 29 (44%) | 111 (29%) | 6 (29%) | 8 (27%) | 143 (31%) |
| Somewhat important | 15 (60%) | 25 (38%) | 194 (50%) | 10 (48%) | 15 (50%) | 225 (48%) |
| Not important | 3 (12%) | 8 (12%) | 59 (15%) | 2 (9.5%) | 3 (10%) | 69 (15%) |
| Don't know | 2 (8.0%) | 4 (6.1%) | 24 (6.2%) | 3 (14%) | 4 (13%) | 27 (5.8%) |
| **How important: That people are aware of how their personal data is being used for AI [privacy]** |  |  |  |  |  |  |
| Very important | 17 (68%) | 47 (71%) | 284 (73%) | 14 (67%) | 24 (80%) | 336 (72%) |
| Somewhat important | 5 (20%) | 13 (20%) | 71 (18%) | 3 (14%) | 5 (17%) | 87 (19%) |
| Not important | 2 (8.0%) | 5 (7.6%) | 21 (5.4%) | 2 (9.5%) | 1 (3.3%) | 27 (5.8%) |
| Don't know | 1 (4.0%) | 1 (1.5%) | 12 (3.1%) | 2 (9.5%) | 0 (0%) | 14 (3.0%) |
| **How important in general: That people can understand which of their individual risk factors for depression are used by the AI [transparency]** |  |  |  |  |  |  |
| Very important | 20 (80%) | 52 (79%) | 324 (84%) | 16 (76%) | 27 (90%) | 382 (82%) |
| Somewhat important | 5 (20%) | 12 (18%) | 50 (13%) | 1 (4.8%) | 3 (10%) | 65 (14%) |
| Not important | 0 (0%) | 1 (1.5%) | 7 (1.8%) | 2 (9.5%) | 0 (0%) | 9 (1.9%) |
| Don't know | 0 (0%) | 1 (1.5%) | 7 (1.8%) | 2 (9.5%) | 0 (0%) | 8 (1.7%) |
| **How important to you: That you are able to make up your own mind about your risk for depression based on AI output [autonomy]** |  |  |  |  |  |  |
| Very important | 19 (76%) | 44 (67%) | 248 (64%) | 14 (67%) | 19 (63%) | 301 (65%) |
| Somewhat important | 5 (20%) | 21 (32%) | 116 (30%) | 5 (24%) | 9 (30%) | 138 (30%) |
| Not important | 1 (4.0%) | 1 (1.5%) | 13 (3.4%) | 1 (4.8%) | 1 (3.3%) | 15 (3.2%) |
| Don't know | 0 (0%) | 0 (0%) | 11 (2.8%) | 1 (4.8%) | 1 (3.3%) | 10 (2.2%) |
| **How important to you: That AI will improve your depression/ depressive symptoms [beneficence]** |  |  |  |  |  |  |
| Very important | 15 (60%) | 38 (58%) | 241 (62%) | 8 (38%) | 23 (77%) | 278 (60%) |
| Somewhat important | 4 (16%) | 23 (35%) | 110 (28%) | 7 (33%) | 6 (20%) | 136 (29%) |
| Not important | 5 (20%) | 4 (6.1%) | 21 (5.4%) | 3 (14%) | 1 (3.3%) | 31 (6.7%) |
| Don't know | 1 (4.0%) | 1 (1.5%) | 16 (4.1%) | 3 (14%) | 0 (0%) | 19 (4.1%) |
| **How important to you: That AI will decrease the chance of negative outcomes [non-maleficence]** |  |  |  |  |  |  |
| Very important | 19 (76%) | 49 (74%) | 277 (71%) | 12 (57%) | 24 (80%) | 329 (71%) |
| Somewhat important | 6 (24%) | 17 (26%) | 95 (24%) | 5 (24%) | 6 (20%) | 117 (25%) |
| Not important | 0 (0%) | 0 (0%) | 11 (2.8%) | 2 (9.5%) | 0 (0%) | 12 (2.6%) |
| Don't know | 0 (0%) | 0 (0%) | 5 (1.3%) | 2 (9.5%) | 0 (0%) | 6 (1.3%) |
| **How important to you: That you can understand how likely it is that you develop depression within the next year according to the AI [justice]** |  |  |  |  |  |  |
| Very important | 14 (56%) | 50 (76%) | 268 (69%) | 11 (52%) | 23 (77%) | 318 (69%) |
| Somewhat important | 10 (40%) | 12 (18%) | 93 (24%) | 6 (29%) | 6 (20%) | 114 (25%) |
| Not important | 0 (0%) | 3 (4.5%) | 10 (2.6%) | 2 (9.5%) | 1 (3.3%) | 13 (2.8%) |
| Don't know | 1 (4.0%) | 1 (1.5%) | 17 (4.4%) | 2 (9.5%) | 0 (0%) | 19 (4.1%) |
| **How important to you: That using AI does not reduce your trust in your mental health care provider [trust]** |  |  |  |  |  |  |
| Very important | 6 (24%) | 30 (45%) | 143 (37%) | 7 (33%) | 12 (40%) | 172 (37%) |
| Somewhat important | 14 (56%) | 27 (41%) | 164 (42%) | 10 (48%) | 15 (50%) | 198 (43%) |
| Not important | 4 (16%) | 4 (6.1%) | 55 (14%) | 2 (9.5%) | 1 (3.3%) | 64 (14%) |
| Don't know | 1 (4.0%) | 5 (7.6%) | 26 (6.7%) | 2 (9.5%) | 2 (6.7%) | 30 (6.5%) |
| **How important to you: That you are aware of how your personal data is being used for AI [privacy]** |  |  |  |  |  |  |
| Very important | 16 (64%) | 51 (77%) | 269 (69%) | 12 (57%) | 22 (73%) | 323 (70%) |
| Somewhat important | 7 (28%) | 10 (15%) | 87 (22%) | 3 (14%) | 5 (17%) | 102 (22%) |
| Not important | 2 (8.0%) | 5 (7.6%) | 23 (5.9%) | 4 (19%) | 3 (10%) | 29 (6.2%) |
| Don't know | 0 (0%) | 0 (0%) | 9 (2.3%) | 2 (9.5%) | 0 (0%) | 10 (2.2%) |
| **How important to you: That you can understand which of your individual risk factors for depression are used by the AI [transparency]** |  |  |  |  |  |  |
| Very important | 21 (84%) | 54 (82%) | 312 (80%) | 15 (71%) | 26 (87%) | 372 (80%) |
| Somewhat important | 4 (16%) | 8 (12%) | 62 (16%) | 3 (14%) | 4 (13%) | 73 (16%) |
| Not important | 0 (0%) | 4 (6.1%) | 12 (3.1%) | 1 (4.8%) | 0 (0%) | 16 (3.4%) |
| Don't know | 0 (0%) | 0 (0%) | 2 (0.5%) | 2 (9.5%) | 0 (0%) | 3 (0.6%) |
| *Statistical significance represented by grey boxes. Statistical significance determined using Fisher's Exact Test for Count Data with simulated p-value (based on 2000 replicates).* | | | | | | |

| **Supplementary Table 4:** Differences in perspectives on AI in mental health by social determinants of health (SDOH; financial resources and education) among survey participants (n=500); n(%) | | | | | | |
| --- | --- | --- | --- | --- | --- | --- |
|  | **Financial resources** | | | **Education** | | |
|  | **More than enough**, n=65 | **Enough**, n=271 | **Not enough**, n=156 | **More than Bachelor's Degree**, n=80 | **Bachelor's Degree**, n=189 | **Less than Bachelor's Degree**, n=230 |
| **General perspectives** |  |  |  |  |  |  |
| **How much do you know about AI and how it could change mental healthcare?** |  |  |  |  |  |  |
| I know quite a lot | 0 (0%) | 4 (1.5%) | 3 (1.9%) | 2 (2.5%) | 2 (1.1%) | 3 (1.3%) |
| I know a fair amount | 10 (15%) | 49 (18%) | 31 (20%) | 11 (14%) | 37 (20%) | 43 (19%) |
| I know a little bit | 34 (52%) | 132 (49%) | 81 (52%) | 48 (60%) | 98 (52%) | 106 (46%) |
| I know almost nothing | 21 (32%) | 86 (32%) | 41 (26%) | 19 (24%) | 52 (28%) | 78 (34%) |
| **Overall, in the next 5 years, do you think AI will make mental healthcare in the United States?** |  |  |  |  |  |  |
| Much better | 5 (7.7%) | 19 (7.0%) | 10 (6.4%) | 2 (2.5%) | 16 (8.5%) | 16 (7.0%) |
| Somewhat better | 24 (37%) | 116 (43%) | 70 (45%) | 38 (48%) | 71 (38%) | 104 (45%) |
| Minimal change | 28 (43%) | 92 (34%) | 46 (29%) | 25 (31%) | 70 (37%) | 74 (32%) |
| Somewhat worse | 2 (3.1%) | 16 (5.9%) | 16 (10%) | 6 (7.5%) | 11 (5.8%) | 17 (7.4%) |
| Much worse | 0 (0%) | 4 (1.5%) | 4 (2.6%) | 0 (0%) | 5 (2.6%) | 3 (1.3%) |
| Don't know | 6 (9.2%) | 24 (8.9%) | 10 (6.4%) | 9 (11%) | 16 (8.5%) | 16 (7.0%) |
| **Comfort with AI** |  |  |  |  |  |  |
| **AI, instead of a mental health professional, performing a mental health assessment** |  |  |  |  |  |  |
| Very comfortable | 8 (12%) | 20 (7.4%) | 15 (9.6%) | 5 (6.2%) | 17 (9.0%) | 21 (9.1%) |
| Somewhat comfortable | 28 (43%) | 100 (37%) | 60 (38%) | 29 (36%) | 77 (41%) | 85 (37%) |
| Somewhat uncomfortable | 17 (26%) | 93 (34%) | 40 (26%) | 28 (35%) | 55 (29%) | 69 (30%) |
| Very uncomfortable | 11 (17%) | 55 (20%) | 39 (25%) | 17 (21%) | 38 (20%) | 51 (22%) |
| Don't know | 1 (1.5%) | 3 (1.1%) | 2 (1.3%) | 1 (1.3%) | 2 (1.1%) | 4 (1.7%) |
| **AI, instead of a mental health professional, making a diagnosis of clinical depression** |  |  |  |  |  |  |
| Very comfortable | 6 (9.2%) | 11 (4.1%) | 9 (5.8%) | 2 (2.5%) | 12 (6.3%) | 12 (5.2%) |
| Somewhat comfortable | 15 (23%) | 73 (27%) | 39 (25%) | 19 (24%) | 46 (24%) | 63 (27%) |
| Somewhat uncomfortable | 22 (34%) | 91 (34%) | 40 (26%) | 26 (32%) | 57 (30%) | 74 (32%) |
| Very uncomfortable | 22 (34%) | 91 (34%) | 64 (41%) | 33 (41%) | 68 (36%) | 77 (33%) |
| Don't know | 0 (0%) | 5 (1.8%) | 4 (2.6%) | 0 (0%) | 6 (3.2%) | 4 (1.7%) |
| **AI, instead of a mental health professional, telling you that you are clinically depressed** |  |  |  |  |  |  |
| Very comfortable | 6 (9.2%) | 12 (4.4%) | 11 (7.1%) | 4 (5.0%) | 13 (6.9%) | 12 (5.2%) |
| Somewhat comfortable | 11 (17%) | 66 (24%) | 41 (26%) | 13 (16%) | 36 (19%) | 70 (30%) |
| Somewhat uncomfortable | 26 (40%) | 80 (30%) | 39 (25%) | 24 (30%) | 58 (31%) | 64 (28%) |
| Very uncomfortable | 21 (32%) | 108 (40%) | 61 (39%) | 37 (46%) | 76 (40%) | 80 (35%) |
| Don't know | 1 (1.5%) | 5 (1.8%) | 4 (2.6%) | 2 (2.5%) | 6 (3.2%) | 4 (1.7%) |
| **AI, instead of a mental health professional, making a diagnosis of bi-polar disorder** |  |  |  |  |  |  |
| Very comfortable | 5 (7.7%) | 10 (3.7%) | 11 (7.1%) | 2 (2.5%) | 11 (5.8%) | 13 (5.7%) |
| Somewhat comfortable | 8 (12%) | 58 (21%) | 28 (18%) | 11 (14%) | 32 (17%) | 51 (22%) |
| Somewhat uncomfortable | 21 (32%) | 82 (30%) | 32 (21%) | 23 (29%) | 48 (25%) | 66 (29%) |
| Very uncomfortable | 31 (48%) | 113 (42%) | 80 (51%) | 40 (50%) | 93 (49%) | 95 (41%) |
| Don't know | 0 (0%) | 8 (3.0%) | 5 (3.2%) | 4 (5.0%) | 5 (2.6%) | 5 (2.2%) |
| **AI, instead of a mental health professional, telling you that you have bi-polar disorder** |  |  |  |  |  |  |
| Very comfortable | 4 (6.2%) | 9 (3.3%) | 10 (6.4%) | 3 (3.8%) | 8 (4.2%) | 12 (5.2%) |
| Somewhat comfortable | 8 (12%) | 44 (16%) | 25 (16%) | 8 (10%) | 26 (14%) | 43 (19%) |
| Somewhat uncomfortable | 20 (31%) | 93 (34%) | 38 (24%) | 26 (32%) | 55 (29%) | 72 (31%) |
| Very uncomfortable | 32 (49%) | 119 (44%) | 77 (49%) | 41 (51%) | 94 (50%) | 97 (42%) |
| Don't know | 1 (1.5%) | 6 (2.2%) | 6 (3.8%) | 2 (2.5%) | 6 (3.2%) | 6 (2.6%) |
| **AI, instead of a mental health professional, recommending a general wellness or stress-management strategy** |  |  |  |  |  |  |
| Very comfortable | 18 (28%) | 66 (24%) | 47 (30%) | 20 (25%) | 46 (24%) | 68 (30%) |
| Somewhat comfortable | 32 (49%) | 124 (46%) | 64 (41%) | 38 (48%) | 87 (46%) | 98 (43%) |
| Somewhat uncomfortable | 7 (11%) | 46 (17%) | 21 (13%) | 12 (15%) | 34 (18%) | 29 (13%) |
| Very uncomfortable | 6 (9.2%) | 32 (12%) | 19 (12%) | 10 (12%) | 19 (10%) | 28 (12%) |
| Don't know | 2 (3.1%) | 3 (1.1%) | 5 (3.2%) | 0 (0%) | 3 (1.6%) | 7 (3.0%) |
| **AI, instead of a mental health professional, recommending a talk therapy** |  |  |  |  |  |  |
| Very comfortable | 12 (18%) | 62 (23%) | 39 (25%) | 20 (25%) | 33 (17%) | 62 (27%) |
| Somewhat comfortable | 31 (48%) | 111 (41%) | 68 (44%) | 32 (40%) | 87 (46%) | 93 (40%) |
| Somewhat uncomfortable | 12 (18%) | 52 (19%) | 22 (14%) | 18 (22%) | 33 (17%) | 36 (16%) |
| Very uncomfortable | 9 (14%) | 40 (15%) | 25 (16%) | 10 (12%) | 32 (17%) | 33 (14%) |
| Don't know | 1 (1.5%) | 6 (2.2%) | 2 (1.3%) | 0 (0%) | 4 (2.1%) | 6 (2.6%) |
| **AI, instead of a mental health professional, recommending a medication** |  |  |  |  |  |  |
| Very comfortable | 7 (11%) | 16 (5.9%) | 10 (6.4%) | 6 (7.5%) | 14 (7.4%) | 13 (5.7%) |
| Somewhat comfortable | 13 (20%) | 56 (21%) | 30 (19%) | 18 (22%) | 35 (19%) | 50 (22%) |
| Somewhat uncomfortable | 19 (29%) | 88 (32%) | 39 (25%) | 24 (30%) | 53 (28%) | 71 (31%) |
| Very uncomfortable | 26 (40%) | 105 (39%) | 75 (48%) | 32 (40%) | 82 (43%) | 93 (40%) |
| Don't know | 0 (0%) | 6 (2.2%) | 2 (1.3%) | 0 (0%) | 5 (2.6%) | 3 (1.3%) |
| **AI, instead of a mental health professional, predicting a patient’s risk for suicide** |  |  |  |  |  |  |
| Very comfortable | 9 (14%) | 22 (8.1%) | 8 (5.1%) | 6 (7.5%) | 15 (7.9%) | 18 (7.8%) |
| Somewhat comfortable | 10 (15%) | 58 (21%) | 30 (19%) | 19 (24%) | 35 (19%) | 45 (20%) |
| Somewhat uncomfortable | 18 (28%) | 78 (29%) | 40 (26%) | 22 (28%) | 50 (26%) | 65 (28%) |
| Very uncomfortable | 27 (42%) | 98 (36%) | 67 (43%) | 27 (34%) | 80 (42%) | 90 (39%) |
| Don't know | 1 (1.5%) | 15 (5.5%) | 11 (7.1%) | 6 (7.5%) | 9 (4.8%) | 12 (5.2%) |
| **AI, instead of a mental health professional, predicting a patient’s risk of engaging in violent behavior** |  |  |  |  |  |  |
| Very comfortable | 7 (11%) | 19 (7.0%) | 12 (7.7%) | 4 (5.0%) | 16 (8.5%) | 18 (7.8%) |
| Somewhat comfortable | 12 (18%) | 62 (23%) | 36 (23%) | 22 (28%) | 37 (20%) | 52 (23%) |
| Somewhat uncomfortable | 18 (28%) | 76 (28%) | 32 (21%) | 17 (21%) | 52 (28%) | 58 (25%) |
| Very uncomfortable | 28 (43%) | 100 (37%) | 64 (41%) | 33 (41%) | 74 (39%) | 89 (39%) |
| Don't know | 0 (0%) | 14 (5.2%) | 12 (7.7%) | 4 (5.0%) | 10 (5.3%) | 13 (5.7%) |
| **Sharing sensitive information with a human mental health professional** |  |  |  |  |  |  |
| Very comfortable | 26 (40%) | 100 (37%) | 55 (35%) | 32 (40%) | 72 (38%) | 77 (33%) |
| Somewhat comfortable | 27 (42%) | 115 (42%) | 62 (40%) | 38 (48%) | 77 (41%) | 93 (40%) |
| Somewhat uncomfortable | 7 (11%) | 46 (17%) | 26 (17%) | 9 (11%) | 28 (15%) | 44 (19%) |
| Very uncomfortable | 5 (7.7%) | 9 (3.3%) | 13 (8.3%) | 1 (1.3%) | 12 (6.3%) | 15 (6.5%) |
| Don't know | 0 (0%) | 1 (0.4%) | 0 (0%) | 0 (0%) | 0 (0%) | 1 (0.4%) |
| **Sharing sensitive information with an AI chatbot** |  |  |  |  |  |  |
| Very comfortable | 11 (17%) | 36 (13%) | 24 (15%) | 12 (15%) | 23 (12%) | 37 (16%) |
| Somewhat comfortable | 18 (28%) | 90 (33%) | 55 (35%) | 25 (31%) | 63 (33%) | 77 (33%) |
| Somewhat uncomfortable | 17 (26%) | 79 (29%) | 33 (21%) | 24 (30%) | 41 (22%) | 65 (28%) |
| Very uncomfortable | 19 (29%) | 63 (23%) | 40 (26%) | 18 (22%) | 60 (32%) | 46 (20%) |
| Don't know | 0 (0%) | 3 (1.1%) | 4 (2.6%) | 1 (1.3%) | 2 (1.1%) | 5 (2.2%) |
| **Sharing sensitive information to help improve AI programs that treat disease** |  |  |  |  |  |  |
| Very comfortable | 21 (32%) | 49 (18%) | 41 (26%) | 17 (21%) | 37 (20%) | 58 (25%) |
| Somewhat comfortable | 18 (28%) | 114 (42%) | 54 (35%) | 35 (44%) | 74 (39%) | 79 (34%) |
| Somewhat uncomfortable | 10 (15%) | 58 (21%) | 31 (20%) | 17 (21%) | 36 (19%) | 47 (20%) |
| Very uncomfortable | 13 (20%) | 36 (13%) | 22 (14%) | 10 (12%) | 35 (19%) | 28 (12%) |
| Don't know | 3 (4.6%) | 14 (5.2%) | 8 (5.1%) | 1 (1.3%) | 7 (3.7%) | 18 (7.8%) |
| **Specific concerns** |  |  |  |  |  |  |
| **That my mental health information will not be kept confidential** |  |  |  |  |  |  |
| Very concerned | 12 (18%) | 55 (20%) | 41 (26%) | 15 (19%) | 49 (26%) | 47 (20%) |
| Somewhat concerned | 23 (35%) | 110 (41%) | 54 (35%) | 29 (36%) | 67 (35%) | 94 (41%) |
| Not concerned | 30 (46%) | 101 (37%) | 61 (39%) | 36 (45%) | 71 (38%) | 86 (37%) |
| Don't know | 0 (0%) | 5 (1.8%) | 0 (0%) | 0 (0%) | 2 (1.1%) | 3 (1.3%) |
| **That the AI will make the wrong diagnosis about my mental health** |  |  |  |  |  |  |
| Very concerned | 20 (31%) | 121 (45%) | 73 (47%) | 32 (40%) | 85 (45%) | 103 (45%) |
| Somewhat concerned | 34 (52%) | 129 (48%) | 64 (41%) | 39 (49%) | 86 (46%) | 103 (45%) |
| Not concerned | 10 (15%) | 18 (6.6%) | 18 (12%) | 8 (10%) | 15 (8.0%) | 23 (10%) |
| Don't know | 1 (1.5%) | 3 (1.1%) | 0 (0%) | 1 (1.3%) | 2 (1.1%) | 1 (0.4%) |
| (Missing) | 0 | 0 | 1 | 0 | 1 | 0 |
| **That the AI will lead to me getting inappropriate treatment for my mental health** |  |  |  |  |  |  |
| Very concerned | 24 (37%) | 117 (43%) | 75 (48%) | 30 (38%) | 93 (49%) | 99 (43%) |
| Somewhat concerned | 30 (46%) | 122 (45%) | 59 (38%) | 40 (50%) | 72 (38%) | 100 (44%) |
| Not concerned | 11 (17%) | 27 (10%) | 21 (13%) | 10 (12%) | 21 (11%) | 28 (12%) |
| Don't know | 0 (0%) | 4 (1.5%) | 1 (0.6%) | 0 (0%) | 3 (1.6%) | 2 (0.9%) |
| (Missing) | 0 | 1 | 0 | 0 | 0 | 1 |
| **That AI will mean I spend less time with my mental health professional** |  |  |  |  |  |  |
| Very concerned | 31 (48%) | 90 (33%) | 53 (34%) | 36 (45%) | 74 (39%) | 66 (29%) |
| Somewhat concerned | 21 (32%) | 100 (37%) | 46 (29%) | 26 (32%) | 62 (33%) | 81 (35%) |
| Not concerned | 12 (18%) | 71 (26%) | 46 (29%) | 16 (20%) | 47 (25%) | 69 (30%) |
| Don't know | 1 (1.5%) | 10 (3.7%) | 11 (7.1%) | 2 (2.5%) | 6 (3.2%) | 14 (6.1%) |
| **That AI will lead to my mental health provider not knowing me as well** |  |  |  |  |  |  |
| Very concerned | 33 (51%) | 104 (38%) | 70 (45%) | 37 (46%) | 83 (44%) | 89 (39%) |
| Somewhat concerned | 23 (35%) | 119 (44%) | 54 (35%) | 31 (39%) | 77 (41%) | 91 (40%) |
| Not concerned | 8 (12%) | 44 (16%) | 26 (17%) | 11 (14%) | 25 (13%) | 44 (19%) |
| Don't know | 1 (1.5%) | 4 (1.5%) | 5 (3.2%) | 1 (1.3%) | 4 (2.1%) | 5 (2.2%) |
| (Missing) | 0 | 0 | 1 | 0 | 0 | 1 |
| **That AI will increase my mental health care costs** |  |  |  |  |  |  |
| Very concerned | 3 (4.6%) | 40 (15%) | 41 (26%) | 4 (5.0%) | 29 (15%) | 52 (23%) |
| Somewhat concerned | 12 (18%) | 70 (26%) | 44 (28%) | 17 (21%) | 43 (23%) | 70 (31%) |
| Not concerned | 44 (68%) | 134 (50%) | 59 (38%) | 51 (64%) | 100 (53%) | 87 (38%) |
| Don't know | 6 (9.2%) | 26 (9.6%) | 12 (7.7%) | 8 (10%) | 17 (9.0%) | 20 (8.7%) |
| (Missing) | 0 | 1 | 0 | 0 | 0 | 1 |
| **Explainability and transparency** |  |  |  |  |  |  |
| **Importance of being told when AI played a big role in mental health diagnosis or treatment** |  |  |  |  |  |  |
| Not important | 4 (6.2%) | 7 (2.6%) | 2 (1.3%) | 4 (5.0%) | 5 (2.6%) | 4 (1.7%) |
| Somewhat important | 13 (20%) | 68 (25%) | 29 (19%) | 15 (19%) | 43 (23%) | 53 (23%) |
| Very important | 47 (72%) | 190 (70%) | 121 (78%) | 58 (72%) | 138 (73%) | 167 (73%) |
| Don't know | 1 (1.5%) | 6 (2.2%) | 4 (2.6%) | 3 (3.8%) | 3 (1.6%) | 6 (2.6%) |
| **Importance of being told when AI played a small role in mental health diagnosis or treatment** |  |  |  |  |  |  |
| Not important | 7 (11%) | 23 (8.5%) | 9 (5.8%) | 8 (10%) | 18 (9.5%) | 13 (5.7%) |
| Somewhat important | 30 (46%) | 111 (41%) | 54 (35%) | 36 (45%) | 75 (40%) | 86 (37%) |
| Very important | 27 (42%) | 133 (49%) | 89 (57%) | 34 (42%) | 95 (50%) | 124 (54%) |
| Don't know | 1 (1.5%) | 4 (1.5%) | 4 (2.6%) | 2 (2.5%) | 1 (0.5%) | 7 (3.0%) |
| **[Given scenario where AI recommends anti-depressant] Importance of being told that AI helped make this decision** |  |  |  |  |  |  |
| Not important | 7 (11%) | 12 (4.4%) | 7 (4.5%) | 8 (10%) | 10 (5.3%) | 8 (3.5%) |
| Somewhat important | 17 (26%) | 62 (23%) | 31 (20%) | 20 (25%) | 44 (23%) | 49 (21%) |
| Very important | 41 (63%) | 194 (72%) | 115 (74%) | 52 (65%) | 133 (70%) | 169 (73%) |
| Don't know | 0 (0%) | 3 (1.1%) | 3 (1.9%) | 0 (0%) | 2 (1.1%) | 4 (1.7%) |
| **Comfort receiving a diagnosis from AI that is 90% accurate but unexplainable** |  |  |  |  |  |  |
| Very comfortable | 3 (4.6%) | 6 (2.2%) | 6 (3.8%) | 4 (5.0%) | 5 (2.6%) | 6 (2.6%) |
| Somewhat comfortable | 19 (29%) | 45 (17%) | 28 (18%) | 11 (14%) | 45 (24%) | 38 (17%) |
| Somewhat uncomfortable | 18 (28%) | 112 (41%) | 51 (33%) | 27 (34%) | 63 (33%) | 94 (41%) |
| Very uncomfortable | 25 (38%) | 107 (39%) | 64 (41%) | 38 (48%) | 75 (40%) | 85 (37%) |
| Don't know | 0 (0%) | 1 (0.4%) | 7 (4.5%) | 0 (0%) | 1 (0.5%) | 7 (3.0%) |
| **Comfort receiving a diagnosis from AI that is 98% accurate but unexplainable** |  |  |  |  |  |  |
| Very comfortable | 14 (22%) | 28 (10%) | 21 (13%) | 5 (6.2%) | 23 (12%) | 35 (15%) |
| Somewhat comfortable | 25 (38%) | 71 (26%) | 40 (26%) | 25 (31%) | 63 (33%) | 50 (22%) |
| Somewhat uncomfortable | 17 (26%) | 103 (38%) | 50 (32%) | 32 (40%) | 55 (29%) | 85 (37%) |
| Very uncomfortable | 9 (14%) | 65 (24%) | 40 (26%) | 18 (22%) | 46 (24%) | 52 (23%) |
| Don't know | 0 (0%) | 4 (1.5%) | 5 (3.2%) | 0 (0%) | 2 (1.1%) | 8 (3.5%) |
| **Trust and responsibility** |  |  |  |  |  |  |
| **[Given scenario where AI and doctor opinion conflicts] How does the computer program affect your view?** |  |  |  |  |  |  |
| It would not affect my trust of the mental health professional’s  assessment | 14 (22%) | 51 (19%) | 20 (13%) | 16 (20%) | 32 (17%) | 37 (16%) |
| It would make me question the mental health professional’s  assessment | 31 (48%) | 140 (52%) | 90 (58%) | 35 (44%) | 93 (49%) | 136 (59%) |
| I do not know if it would change my view of the mental health  professional’s assessment | 19 (29%) | 73 (27%) | 41 (26%) | 26 (32%) | 57 (30%) | 54 (23%) |
| Don't know | 1 (1.5%) | 7 (2.6%) | 5 (3.2%) | 3 (3.8%) | 7 (3.7%) | 3 (1.3%) |
| **Responsibility for medical errors resulting from AI (select all that apply)** |  |  |  |  |  |  |
| Mental health professional | 53 (82%) | 231 (85%) | 121 (78%) | 68 (85%) | 159 (84%) | 185 (80%) |
| Company that made the computer program | 20 (31%) | 94 (35%) | 62 (40%) | 28 (35%) | 74 (39%) | 80 (35%) |
| Hospital or clinic that bought the computer program | 14 (22%) | 80 (30%) | 56 (36%) | 23 (29%) | 59 (31%) | 71 (31%) |
| Government agency that approved the computer program | 11 (17%) | 57 (21%) | 44 (28%) | 14 (18%) | 40 (21%) | 62 (27%) |
| Someone else | 1 (1.5%) | 7 (2.6%) | 9 (5.8%) | 4 (5.0%) | 6 (3.2%) | 7 (3.0%) |
| No one | 0 (0%) | 0 (0%) | 1 (0.6%) | 0 (0%) | 0 (0%) | 1 (0.4%) |
| Don't know^†^ | 2 (3.1%) | 16 (5.9%) | 9 (5.8%) | 0 (0%) | 10 (5.3%) | 17 (7.4%) |
| **Blame for medical errors resulting from AI (select all that apply)** |  |  |  |  |  |  |
| Mental health professional | 50 (77%) | 227 (84%) | 124 (79%) | 66 (82%) | 156 (83%) | 185 (80%) |
| Company that made the computer program | 21 (32%) | 86 (32%) | 56 (36%) | 26 (32%) | 67 (35%) | 76 (33%) |
| Hospital or clinic that bought the computer program* | 11 (17%) | 64 (24%) | 46 (29%) | 16 (20%) | 48 (25%) | 61 (27%) |
| Government agency that approved the computer program | 10 (15%) | 49 (18%) | 42 (27%) | 10 (12%) | 37 (20%) | 58 (25%) |
| Someone else* | 2 (3.1%) | 1 (0.4%) | 6 (3.8%) | 3 (3.8%) | 3 (1.6%) | 4 (1.7%) |
| No one | 3 (4.6%) | 11 (4.1%) | 5 (3.2%) | 3 (3.8%) | 7 (3.7%) | 9 (3.9%) |
| Don't know | 0 (0%) | 9 (3.3%) | 6 (3.8%) | 2 (2.5%) | 6 (3.2%) | 7 (3.0%) |
| **Responsibility for checking that AI is safe (select all that apply)** |  |  |  |  |  |  |
| Mental health professional | 23 (35%) | 71 (26%) | 39 (25%) | 30 (38%) | 47 (25%) | 57 (25%) |
| Company that made the computer program | 41 (63%) | 174 (64%) | 109 (70%) | 57 (71%) | 127 (67%) | 144 (63%) |
| Hospital or clinic that bought the computer program | 41 (63%) | 149 (55%) | 88 (56%) | 52 (65%) | 106 (56%) | 124 (54%) |
| Government agency that approved the computer program | 33 (51%) | 132 (49%) | 69 (44%) | 33 (41%) | 88 (47%) | 117 (51%) |
| Someone else | 3 (4.6%) | 3 (1.1%) | 1 (0.6%) | 2 (2.5%) | 4 (2.1%) | 1 (0.4%) |
| No one | 1 (1.5%) | 8 (3.0%) | 8 (5.1%) | 1 (1.3%) | 8 (4.2%) | 9 (3.9%) |
| Don't know | 0 (0%) | 6 (2.2%) | 6 (3.8%) | 0 (0%) | 5 (2.6%) | 8 (3.5%) |
| **Importance of bioethical constructs** |  |  |  |  |  |  |
| **How important in general: That people are able to make up their own mind about their risk for depression based on AI output [autonomy]** |  |  |  |  |  |  |
| Very important | 30 (46%) | 144 (53%) | 101 (65%) | 40 (50%) | 100 (53%) | 139 (60%) |
| Somewhat important | 32 (49%) | 101 (37%) | 43 (28%) | 35 (44%) | 70 (37%) | 73 (32%) |
| Not important | 1 (1.5%) | 16 (5.9%) | 5 (3.2%) | 2 (2.5%) | 10 (5.3%) | 10 (4.3%) |
| Don't know | 2 (3.1%) | 10 (3.7%) | 7 (4.5%) | 3 (3.8%) | 9 (4.8%) | 8 (3.5%) |
| **How important in general: That AI will improve depressive symptoms [beneficence]** |  |  |  |  |  |  |
| Very important | 24 (37%) | 122 (45%) | 77 (49%) | 33 (41%) | 86 (46%) | 108 (47%) |
| Somewhat important | 33 (51%) | 108 (40%) | 57 (37%) | 39 (49%) | 77 (41%) | 83 (36%) |
| Not important | 3 (4.6%) | 24 (8.9%) | 10 (6.4%) | 2 (2.5%) | 18 (9.5%) | 18 (7.8%) |
| Don't know | 5 (7.7%) | 17 (6.3%) | 12 (7.7%) | 6 (7.5%) | 8 (4.2%) | 21 (9.1%) |
| **How important in general: That AI will reduce the chance of negative outcomes [non-maleficence]** |  |  |  |  |  |  |
| Very important | 40 (62%) | 172 (63%) | 120 (77%) | 51 (64%) | 122 (65%) | 163 (71%) |
| Somewhat important | 20 (31%) | 88 (32%) | 26 (17%) | 24 (30%) | 57 (30%) | 56 (24%) |
| Not important | 4 (6.2%) | 7 (2.6%) | 5 (3.2%) | 4 (5.0%) | 5 (2.6%) | 7 (3.0%) |
| Don't know | 1 (1.5%) | 4 (1.5%) | 5 (3.2%) | 1 (1.3%) | 5 (2.6%) | 4 (1.7%) |
| **How important in general: That people can understand how likely it is that they develop depression in the next year according to the AI [justice]** |  |  |  |  |  |  |
| Very important | 43 (66%) | 172 (63%) | 110 (71%) | 54 (68%) | 122 (65%) | 152 (66%) |
| Somewhat important | 19 (29%) | 76 (28%) | 36 (23%) | 25 (31%) | 53 (28%) | 55 (24%) |
| Not important | 1 (1.5%) | 11 (4.1%) | 3 (1.9%) | 0 (0%) | 8 (4.2%) | 7 (3.0%) |
| Don't know | 2 (3.1%) | 12 (4.4%) | 7 (4.5%) | 1 (1.3%) | 6 (3.2%) | 16 (7.0%) |
| **How important in general: That AI does not reduce people’s trust in their mental health care professionals [trust]** |  |  |  |  |  |  |
| Very important | 20 (31%) | 82 (30%) | 48 (31%) | 19 (24%) | 61 (32%) | 71 (31%) |
| Somewhat important | 35 (54%) | 134 (49%) | 70 (45%) | 45 (56%) | 86 (46%) | 112 (49%) |
| Not important | 7 (11%) | 41 (15%) | 23 (15%) | 11 (14%) | 28 (15%) | 33 (14%) |
| Don't know | 3 (4.6%) | 14 (5.2%) | 15 (9.6%) | 5 (6.2%) | 14 (7.4%) | 14 (6.1%) |
| **How important in general: That people are aware of how their personal data is being used for AI [privacy]** |  |  |  |  |  |  |
| Very important | 46 (71%) | 185 (68%) | 125 (80%) | 61 (76%) | 133 (70%) | 167 (73%) |
| Somewhat important | 15 (23%) | 56 (21%) | 19 (12%) | 14 (18%) | 37 (20%) | 41 (18%) |
| Not important | 3 (4.6%) | 21 (7.7%) | 6 (3.8%) | 5 (6.2%) | 11 (5.8%) | 14 (6.1%) |
| Don't know | 1 (1.5%) | 9 (3.3%) | 6 (3.8%) | 0 (0%) | 8 (4.2%) | 8 (3.5%) |
| **How important in general: That people can understand which of their individual risk factors for depression are used by the AI [transparency]** |  |  |  |  |  |  |
| Very important | 53 (82%) | 220 (81%) | 132 (85%) | 69 (86%) | 148 (78%) | 194 (84%) |
| Somewhat important | 11 (17%) | 41 (15%) | 15 (9.6%) | 9 (11%) | 33 (17%) | 26 (11%) |
| Not important | 0 (0%) | 6 (2.2%) | 4 (2.6%) | 0 (0%) | 5 (2.6%) | 5 (2.2%) |
| Don't know | 1 (1.5%) | 4 (1.5%) | 5 (3.2%) | 2 (2.5%) | 3 (1.6%) | 5 (2.2%) |
| **How important to you: That you are able to make up your own mind about your risk for depression based on AI output [autonomy]** |  |  |  |  |  |  |
| Very important | 36 (55%) | 171 (63%) | 115 (74%) | 45 (56%) | 117 (62%) | 162 (70%) |
| Somewhat important | 26 (40%) | 82 (30%) | 35 (22%) | 29 (36%) | 58 (31%) | 60 (26%) |
| Not important | 1 (1.5%) | 12 (4.4%) | 3 (1.9%) | 4 (5.0%) | 9 (4.8%) | 3 (1.3%) |
| Don't know | 2 (3.1%) | 6 (2.2%) | 3 (1.9%) | 2 (2.5%) | 5 (2.6%) | 5 (2.2%) |
| **How important to you: That AI will improve your depression/ depressive symptoms [beneficence]** |  |  |  |  |  |  |
| Very important | 40 (62%) | 165 (61%) | 94 (60%) | 45 (56%) | 110 (58%) | 147 (64%) |
| Somewhat important | 20 (31%) | 76 (28%) | 45 (29%) | 28 (35%) | 61 (32%) | 54 (23%) |
| Not important | 3 (4.6%) | 20 (7.4%) | 9 (5.8%) | 4 (5.0%) | 11 (5.8%) | 18 (7.8%) |
| Don't know | 2 (3.1%) | 10 (3.7%) | 8 (5.1%) | 3 (3.8%) | 7 (3.7%) | 11 (4.8%) |
| **How important to you: That AI will decrease the chance of negative outcomes [non-maleficence]** |  |  |  |  |  |  |
| Very important | 46 (71%) | 181 (67%) | 126 (81%) | 59 (74%) | 126 (67%) | 171 (74%) |
| Somewhat important | 16 (25%) | 79 (29%) | 25 (16%) | 20 (25%) | 53 (28%) | 50 (22%) |
| Not important | 2 (3.1%) | 8 (3.0%) | 3 (1.9%) | 1 (1.3%) | 7 (3.7%) | 5 (2.2%) |
| Don't know | 1 (1.5%) | 3 (1.1%) | 2 (1.3%) | 0 (0%) | 3 (1.6%) | 4 (1.7%) |
| **How important to you: That you can understand how likely it is that you develop depression within the next year according to the AI [justice]** |  |  |  |  |  |  |
| Very important | 49 (75%) | 177 (65%) | 114 (73%) | 57 (71%) | 124 (66%) | 161 (70%) |
| Somewhat important | 14 (22%) | 72 (27%) | 32 (21%) | 22 (28%) | 53 (28%) | 46 (20%) |
| Not important | 0 (0%) | 11 (4.1%) | 4 (2.6%) | 1 (1.3%) | 6 (3.2%) | 8 (3.5%) |
| Don't know | 2 (3.1%) | 11 (4.1%) | 6 (3.8%) | 0 (0%) | 6 (3.2%) | 15 (6.5%) |
| **How important to you: That using AI does not reduce your trust in your mental health care provider [trust]** |  |  |  |  |  |  |
| Very important | 26 (40%) | 99 (37%) | 60 (38%) | 34 (42%) | 70 (37%) | 82 (36%) |
| Somewhat important | 30 (46%) | 121 (45%) | 61 (39%) | 31 (39%) | 81 (43%) | 103 (45%) |
| Not important | 6 (9.2%) | 36 (13%) | 22 (14%) | 10 (12%) | 28 (15%) | 27 (12%) |
| Don't know | 3 (4.6%) | 15 (5.5%) | 13 (8.3%) | 5 (6.2%) | 10 (5.3%) | 18 (7.8%) |
| **How important to you: That you are aware of how your personal data is being used for AI [privacy]** |  |  |  |  |  |  |
| Very important | 46 (71%) | 181 (67%) | 116 (74%) | 58 (72%) | 128 (68%) | 161 (70%) |
| Somewhat important | 14 (22%) | 66 (24%) | 26 (17%) | 16 (20%) | 45 (24%) | 46 (20%) |
| Not important | 4 (6.2%) | 20 (7.4%) | 8 (5.1%) | 6 (7.5%) | 13 (6.9%) | 15 (6.5%) |
| Don't know | 1 (1.5%) | 4 (1.5%) | 6 (3.8%) | 0 (0%) | 3 (1.6%) | 8 (3.5%) |
| **How important to you: That you can understand which of your individual risk factors for depression are used by the AI [transparency]** |  |  |  |  |  |  |
| Very important | 49 (75%) | 214 (79%) | 133 (85%) | 66 (82%) | 144 (76%) | 191 (83%) |
| Somewhat important | 13 (20%) | 46 (17%) | 17 (11%) | 12 (15%) | 36 (19%) | 29 (13%) |
| Not important | 2 (3.1%) | 10 (3.7%) | 4 (2.6%) | 2 (2.5%) | 8 (4.2%) | 7 (3.0%) |
| Don't know | 1 (1.5%) | 1 (0.4%) | 2 (1.3%) | 0 (0%) | 1 (0.5%) | 3 (1.3%) |
| *Statistical significance represented by grey boxes. Statistical significance determined using Fisher's Exact Test for Count Data with simulated p-value (based on 2000 replicates).* | | | | | | |

| **Supplementary Table 5:** Differences in perspectives on AI in mental health by social determinants of health (SDOH; health literacy and subjective numeracy) among survey participants (n=500); n(%) | | | | |
| --- | --- | --- | --- | --- |
|  | **Health Literacy** | | **Subjective numeracy** | |
|  | **Adequate**, n=369 | **Inadequate**, n=131 | **High (>14),** n=216 | **Low (<=14),** n=283 |
| **General perspectives** |  |  |  |  |
| **How much do you know about AI and how it could change mental healthcare?** |  |  |  |  |
| I know quite a lot | 5 (1.4%) | 2 (1.5%) | 3 (1.4%) | 4 (1.4%) |
| I know a fair amount | 66 (18%) | 26 (20%) | 49 (23%) | 43 (15%) |
| I know a little bit | 183 (50%) | 69 (53%) | 109 (50%) | 143 (51%) |
| I know almost nothing | 115 (31%) | 34 (26%) | 55 (25%) | 93 (33%) |
| **Overall, in the next 5 years, do you think AI will make mental healthcare in the United States?** |  |  |  |  |
| Much better | 20 (5.4%) | 14 (11%) | 16 (7.4%) | 18 (6.4%) |
| Somewhat better | 145 (39%) | 68 (52%) | 94 (44%) | 119 (42%) |
| Minimal change | 134 (36%) | 36 (27%) | 72 (33%) | 98 (35%) |
| Somewhat worse | 29 (7.9%) | 5 (3.8%) | 17 (7.9%) | 17 (6.0%) |
| Much worse | 7 (1.9%) | 1 (0.8%) | 2 (0.9%) | 6 (2.1%) |
| Don't know | 34 (9.2%) | 7 (5.3%) | 15 (6.9%) | 25 (8.8%) |
| **Comfort with AI** |  |  |  |  |
| **AI, instead of a mental health professional, performing a mental health assessment** |  |  |  |  |
| Very comfortable | 30 (8.1%) | 13 (9.9%) | 26 (12%) | 17 (6.0%) |
| Somewhat comfortable | 143 (39%) | 48 (37%) | 83 (38%) | 108 (38%) |
| Somewhat uncomfortable | 109 (30%) | 44 (34%) | 66 (31%) | 86 (30%) |
| Very uncomfortable | 83 (22%) | 23 (18%) | 40 (19%) | 66 (23%) |
| Don't know | 4 (1.1%) | 3 (2.3%) | 1 (0.5%) | 6 (2.1%) |
| **AI, instead of a mental health professional, making a diagnosis of clinical depression** |  |  |  |  |
| Very comfortable | 21 (5.7%) | 5 (3.8%) | 16 (7.4%) | 10 (3.5%) |
| Somewhat comfortable | 92 (25%) | 36 (27%) | 55 (25%) | 73 (26%) |
| Somewhat uncomfortable | 110 (30%) | 48 (37%) | 73 (34%) | 84 (30%) |
| Very uncomfortable | 140 (38%) | 38 (29%) | 69 (32%) | 109 (39%) |
| Don't know | 6 (1.6%) | 4 (3.1%) | 3 (1.4%) | 7 (2.5%) |
| **AI, instead of a mental health professional, telling you that you are clinically depressed** |  |  |  |  |
| Very comfortable | 20 (5.4%) | 9 (6.9%) | 19 (8.8%) | 10 (3.5%) |
| Somewhat comfortable | 79 (21%) | 40 (31%) | 44 (20%) | 75 (27%) |
| Somewhat uncomfortable | 106 (29%) | 41 (31%) | 76 (35%) | 70 (25%) |
| Very uncomfortable | 154 (42%) | 39 (30%) | 73 (34%) | 120 (42%) |
| Don't know | 10 (2.7%) | 2 (1.5%) | 4 (1.9%) | 8 (2.8%) |
| **AI, instead of a mental health professional, making a diagnosis of bi-polar disorder** |  |  |  |  |
| Very comfortable | 19 (5.1%) | 7 (5.3%) | 17 (7.9%) | 9 (3.2%) |
| Somewhat comfortable | 67 (18%) | 27 (21%) | 36 (17%) | 58 (20%) |
| Somewhat uncomfortable | 95 (26%) | 42 (32%) | 61 (28%) | 75 (27%) |
| Very uncomfortable | 179 (49%) | 50 (38%) | 97 (45%) | 132 (47%) |
| Don't know | 9 (2.4%) | 5 (3.8%) | 5 (2.3%) | 9 (3.2%) |
| **AI, instead of a mental health professional, telling you that you have bi-polar disorder** |  |  |  |  |
| Very comfortable | 16 (4.3%) | 7 (5.3%) | 12 (5.6%) | 11 (3.9%) |
| Somewhat comfortable | 57 (15%) | 20 (15%) | 31 (14%) | 46 (16%) |
| Somewhat uncomfortable | 105 (28%) | 48 (37%) | 69 (32%) | 83 (29%) |
| Very uncomfortable | 182 (49%) | 51 (39%) | 98 (45%) | 135 (48%) |
| Don't know | 9 (2.4%) | 5 (3.8%) | 6 (2.8%) | 8 (2.8%) |
| **AI, instead of a mental health professional, recommending a general wellness or stress-management strategy** |  |  |  |  |
| Very comfortable | 97 (26%) | 37 (28%) | 62 (29%) | 72 (25%) |
| Somewhat comfortable | 169 (46%) | 55 (42%) | 96 (44%) | 128 (45%) |
| Somewhat uncomfortable | 50 (14%) | 25 (19%) | 35 (16%) | 39 (14%) |
| Very uncomfortable | 47 (13%) | 10 (7.6%) | 20 (9.3%) | 37 (13%) |
| Don't know | 6 (1.6%) | 4 (3.1%) | 3 (1.4%) | 7 (2.5%) |
| **AI, instead of a mental health professional, recommending a talk therapy** |  |  |  |  |
| Very comfortable | 84 (23%) | 31 (24%) | 54 (25%) | 61 (22%) |
| Somewhat comfortable | 155 (42%) | 57 (44%) | 93 (43%) | 119 (42%) |
| Somewhat uncomfortable | 64 (17%) | 24 (18%) | 38 (18%) | 49 (17%) |
| Very uncomfortable | 59 (16%) | 16 (12%) | 29 (13%) | 46 (16%) |
| Don't know | 7 (1.9%) | 3 (2.3%) | 2 (0.9%) | 8 (2.8%) |
| **AI, instead of a mental health professional, recommending a medication** |  |  |  |  |
| Very comfortable | 25 (6.8%) | 8 (6.1%) | 15 (6.9%) | 18 (6.4%) |
| Somewhat comfortable | 66 (18%) | 37 (28%) | 50 (23%) | 53 (19%) |
| Somewhat uncomfortable | 111 (30%) | 38 (29%) | 65 (30%) | 83 (29%) |
| Very uncomfortable | 162 (44%) | 45 (34%) | 83 (38%) | 124 (44%) |
| Don't know | 5 (1.4%) | 3 (2.3%) | 3 (1.4%) | 5 (1.8%) |
| **AI, instead of a mental health professional, predicting a patient’s risk for suicide^§^** |  |  |  |  |
| Very comfortable | 29 (7.9%) | 10 (7.6%) | 29 (13%) | 10 (3.5%) |
| Somewhat comfortable | 69 (19%) | 31 (24%) | 38 (18%) | 62 (22%) |
| Somewhat uncomfortable | 101 (27%) | 36 (27%) | 58 (27%) | 78 (28%) |
| Very uncomfortable | 149 (40%) | 48 (37%) | 84 (39%) | 113 (40%) |
| Don't know | 21 (5.7%) | 6 (4.6%) | 7 (3.2%) | 20 (7.1%) |
| **AI, instead of a mental health professional, predicting a patient’s risk of engaging in violent behavior** |  |  |  |  |
| Very comfortable | 31 (8.4%) | 7 (5.3%) | 25 (12%) | 13 (4.6%) |
| Somewhat comfortable | 74 (20%) | 38 (29%) | 42 (19%) | 70 (25%) |
| Somewhat uncomfortable | 91 (25%) | 36 (27%) | 58 (27%) | 68 (24%) |
| Very uncomfortable | 153 (41%) | 43 (33%) | 81 (38%) | 115 (41%) |
| Don't know | 20 (5.4%) | 7 (5.3%) | 10 (4.6%) | 17 (6.0%) |
| **Sharing sensitive information a human mental health professional** |  |  |  |  |
| Very comfortable | 140 (38%) | 41 (31%) | 86 (40%) | 95 (34%) |
| Somewhat comfortable | 151 (41%) | 57 (44%) | 88 (41%) | 119 (42%) |
| Somewhat uncomfortable | 59 (16%) | 23 (18%) | 31 (14%) | 51 (18%) |
| Very uncomfortable | 19 (5.1%) | 9 (6.9%) | 11 (5.1%) | 17 (6.0%) |
| Don't know | 0 (0%) | 1 (0.8%) | 0 (0%) | 1 (0.4%) |
| **Sharing sensitive information with an AI chatbot** |  |  |  |  |
| Very comfortable | 54 (15%) | 18 (14%) | 28 (13%) | 44 (16%) |
| Somewhat comfortable | 119 (32%) | 46 (35%) | 74 (34%) | 91 (32%) |
| Somewhat uncomfortable | 89 (24%) | 41 (31%) | 51 (24%) | 78 (28%) |
| Very uncomfortable | 104 (28%) | 21 (16%) | 60 (28%) | 65 (23%) |
| Don't know | 3 (0.8%) | 5 (3.8%) | 3 (1.4%) | 5 (1.8%) |
| **Sharing sensitive information to help improve AI programs that treat disease** |  |  |  |  |
| Very comfortable | 83 (22%) | 29 (22%) | 53 (25%) | 59 (21%) |
| Somewhat comfortable | 132 (36%) | 56 (43%) | 76 (35%) | 112 (40%) |
| Somewhat uncomfortable | 68 (18%) | 32 (24%) | 43 (20%) | 56 (20%) |
| Very uncomfortable | 66 (18%) | 8 (6.1%) | 37 (17%) | 37 (13%) |
| Don't know | 20 (5.4%) | 6 (4.6%) | 7 (3.2%) | 19 (6.7%) |
| **Specific concerns** |  |  |  |  |
| **That my mental health information will not be kept confidential** |  |  |  |  |
| Very concerned | 87 (24%) | 25 (19%) | 56 (26%) | 56 (20%) |
| Somewhat concerned | 129 (35%) | 61 (47%) | 74 (34%) | 115 (41%) |
| Not concerned | 150 (41%) | 43 (33%) | 83 (38%) | 110 (39%) |
| Don't know | 3 (0.8%) | 2 (1.5%) | 3 (1.4%) | 2 (0.7%) |
| **That the AI will make the wrong diagnosis about my mental health** |  |  |  |  |
| Very concerned | 158 (43%) | 63 (48%) | 94 (44%) | 126 (45%) |
| Somewhat concerned | 175 (48%) | 53 (40%) | 98 (46%) | 130 (46%) |
| Not concerned | 31 (8.4%) | 15 (11%) | 21 (9.8%) | 25 (8.8%) |
| Don't know | 4 (1.1%) | 0 (0%) | 2 (0.9%) | 2 (0.7%) |
| (Missing) | 1 | 0 | 1 | 0 |
| **That the AI will lead to me getting inappropriate treatment for my mental health** |  |  |  |  |
| Very concerned | 164 (44%) | 59 (45%) | 102 (47%) | 121 (43%) |
| Somewhat concerned | 158 (43%) | 54 (42%) | 81 (38%) | 130 (46%) |
| Not concerned | 43 (12%) | 16 (12%) | 30 (14%) | 29 (10%) |
| Don't know | 4 (1.1%) | 1 (0.8%) | 3 (1.4%) | 2 (0.7%) |
| (Missing) | 0 | 1 | 0 | 1 |
| **That AI will mean I spend less time with my mental health professional** |  |  |  |  |
| Very concerned | 147 (40%) | 29 (22%) | 84 (39%) | 92 (33%) |
| Somewhat concerned | 116 (31%) | 54 (41%) | 73 (34%) | 96 (34%) |
| Not concerned | 90 (24%) | 42 (32%) | 51 (24%) | 81 (29%) |
| Don't know | 16 (4.3%) | 6 (4.6%) | 8 (3.7%) | 14 (4.9%) |
| **That AI will lead to my mental health provider not knowing me as well** |  |  |  |  |
| Very concerned | 164 (44%) | 45 (35%) | 97 (45%) | 112 (40%) |
| Somewhat concerned | 139 (38%) | 61 (47%) | 84 (39%) | 115 (41%) |
| Not concerned | 57 (15%) | 23 (18%) | 29 (13%) | 51 (18%) |
| Don't know | 9 (2.4%) | 1 (0.8%) | 6 (2.8%) | 4 (1.4%) |
| (Missing) | 0 | 1 | 0 | 1 |
| **That AI will increase my mental health care costs** |  |  |  |  |
| Very concerned | 56 (15%) | 29 (22%) | 35 (16%) | 49 (17%) |
| Somewhat concerned | 93 (25%) | 38 (29%) | 42 (20%) | 89 (31%) |
| Not concerned | 184 (50%) | 54 (41%) | 112 (52%) | 126 (45%) |
| Don't know | 35 (9.5%) | 10 (7.6%) | 26 (12%) | 19 (6.7%) |
| (Missing) | 1 | 0 | 1 | 0 |
| **Explainability and transparency** |  |  |  |  |
| **Importance of being told when AI played a big role in mental health diagnosis or treatment** |  |  |  |  |
| Not important | 10 (2.7%) | 3 (2.3%) | 7 (3.2%) | 6 (2.1%) |
| Somewhat important | 82 (22%) | 29 (22%) | 46 (21%) | 65 (23%) |
| Very important | 269 (73%) | 95 (73%) | 160 (74%) | 203 (72%) |
| Don't know | 8 (2.2%) | 4 (3.1%) | 3 (1.4%) | 9 (3.2%) |
| **Importance of being told when AI played a small role in mental health diagnosis or treatment** |  |  |  |  |
| Not important | 31 (8.4%) | 8 (6.1%) | 16 (7.4%) | 23 (8.1%) |
| Somewhat important | 151 (41%) | 47 (36%) | 81 (38%) | 117 (41%) |
| Very important | 181 (49%) | 72 (55%) | 116 (54%) | 136 (48%) |
| Don't know | 6 (1.6%) | 4 (3.1%) | 3 (1.4%) | 7 (2.5%) |
| **[Given scenario where AI recommends anti-depressant] Importance of being told that AI helped make this decision** |  |  |  |  |
| Not important | 20 (5.4%) | 6 (4.6%) | 14 (6.5%) | 12 (4.2%) |
| Somewhat important | 83 (22%) | 30 (23%) | 44 (20%) | 69 (24%) |
| Very important | 263 (71%) | 92 (70%) | 158 (73%) | 196 (69%) |
| Don't know | 3 (0.8%) | 3 (2.3%) | 0 (0%) | 6 (2.1%) |
| **Comfort receiving a diagnosis from AI that is 90% accurate but unexplainable** |  |  |  |  |
| Very comfortable | 12 (3.3%) | 3 (2.3%) | 6 (2.8%) | 9 (3.2%) |
| Somewhat comfortable | 64 (17%) | 30 (23%) | 50 (23%) | 44 (16%) |
| Somewhat uncomfortable | 127 (34%) | 57 (44%) | 77 (36%) | 106 (37%) |
| Very uncomfortable | 161 (44%) | 38 (29%) | 81 (38%) | 118 (42%) |
| Don't know | 5 (1.4%) | 3 (2.3%) | 2 (0.9%) | 6 (2.1%) |
| **Comfort receiving a diagnosis from AI that is 98% accurate but unexplainable** |  |  |  |  |
| Very comfortable | 45 (12%) | 18 (14%) | 32 (15%) | 31 (11%) |
| Somewhat comfortable | 97 (26%) | 41 (31%) | 65 (30%) | 73 (26%) |
| Somewhat uncomfortable | 125 (34%) | 47 (36%) | 75 (35%) | 96 (34%) |
| Very uncomfortable | 95 (26%) | 22 (17%) | 41 (19%) | 76 (27%) |
| Don't know | 7 (1.9%) | 3 (2.3%) | 3 (1.4%) | 7 (2.5%) |
| **Trust and responsibility** |  |  |  |  |
| **[Given scenario where AI and doctor opinion conflicts] How does the computer program affect your view?** |  |  |  |  |
| It would not affect my trust of the mental health professional’s assessment | 66 (18%) | 19 (15%) | 47 (22%) | 38 (13%) |
| It would make me question the mental health professional’s assessment | 187 (51%) | 78 (60%) | 102 (47%) | 162 (57%) |
| I do not know if it would change my view of the mental health professional’s assessment | 107 (29%) | 30 (23%) | 60 (28%) | 77 (27%) |
| Don't know | 9 (2.4%) | 4 (3.1%) | 7 (3.2%) | 6 (2.1%) |
| **Responsibility for medical errors resulting from AI (select all that apply)** |  |  |  |  |
| Mental health professional | 308 (83%) | 104 (79%) | 182 (84%) | 229 (81%) |
| Company that made the computer program | 136 (37%) | 47 (36%) | 91 (42%) | 92 (33%) |
| Hospital or clinic that bought the computer program | 105 (28%) | 48 (37%) | 70 (32%) | 82 (29%) |
| Government agency that approved the computer program | 83 (22%) | 33 (25%) | 50 (23%) | 66 (23%) |
| Someone else | 15 (4.1%) | 2 (1.5%) | 6 (2.8%) | 11 (3.9%) |
| No one | 1 (0.3%) | 0 (0%) | 0 (0%) | 1 (0.4%) |
| Don't know | 20 (5.4%) | 7 (5.3%) | 9 (4.2%) | 18 (6.4%) |
| **Blame for medical errors resulting from AI (select all that apply)** |  |  |  |  |
| Mental health professional | 304 (82%) | 104 (79%) | 179 (83%) | 228 (81%) |
| Company that made the computer program | 121 (33%) | 48 (37%) | 80 (37%) | 89 (31%) |
| Hospital or clinic that bought the computer program | 82 (22%) | 43 (33%) | 55 (25%) | 69 (24%) |
| Government agency that approved the computer program | 71 (19%) | 34 (26%) | 42 (19%) | 63 (22%) |
| Someone else | 7 (1.9%) | 3 (2.3%) | 4 (1.9%) | 6 (2.1%) |
| No one | 16 (4.3%) | 3 (2.3%) | 9 (4.2%) | 10 (3.5%) |
| Don't know | 13 (3.5%) | 2 (1.5%) | 3 (1.4%) | 12 (4.2%) |
| **Responsibility for checking that AI is safe (select all that apply)** |  |  |  |  |
| Mental health professional | 92 (25%) | 43 (33%) | 59 (27%) | 76 (27%) |
| Company that made the computer program | 242 (66%) | 86 (66%) | 150 (69%) | 178 (63%) |
| Hospital or clinic that bought the computer program | 205 (56%) | 77 (59%) | 118 (55%) | 163 (58%) |
| Government agency that approved the computer program | 179 (49%) | 59 (45%) | 101 (47%) | 137 (48%) |
| Someone else | 6 (1.6%) | 1 (0.8%) | 4 (1.9%) | 3 (1.1%) |
| No one | 13 (3.5%) | 5 (3.8%) | 7 (3.2%) | 11 (3.9%) |
| Don't know | 10 (2.7%) | 3 (2.3%) | 4 (1.9%) | 9 (3.2%) |
| **Importance of bioethical constructs** |  |  |  |  |
| **How important in general: That people are able to make up their own mind about their risk for depression based on AI output [autonomy]** |  |  |  |  |
| Very important | 198 (54%) | 82 (63%) | 115 (53%) | 164 (58%) |
| Somewhat important | 136 (37%) | 42 (32%) | 81 (38%) | 97 (34%) |
| Not important | 18 (4.9%) | 4 (3.1%) | 12 (5.6%) | 10 (3.5%) |
| Don't know | 17 (4.6%) | 3 (2.3%) | 8 (3.7%) | 12 (4.2%) |
| **How important in general: That AI will improve depressive symptoms [beneficence]** |  |  |  |  |
| Very important | 169 (46%) | 58 (44%) | 94 (44%) | 133 (47%) |
| Somewhat important | 142 (38%) | 57 (44%) | 95 (44%) | 103 (36%) |
| Not important | 28 (7.6%) | 10 (7.6%) | 13 (6.0%) | 25 (8.8%) |
| Don't know | 30 (8.1%) | 6 (4.6%) | 14 (6.5%) | 22 (7.8%) |
| **How important in general: That AI will reduce the chance of negative outcomes [non-maleficence]** |  |  |  |  |
| Very important | 250 (68%) | 87 (66%) | 140 (65%) | 196 (69%) |
| Somewhat important | 98 (27%) | 39 (30%) | 62 (29%) | 75 (27%) |
| Not important | 13 (3.5%) | 3 (2.3%) | 10 (4.6%) | 6 (2.1%) |
| Don't know | 8 (2.2%) | 2 (1.5%) | 4 (1.9%) | 6 (2.1%) |
| **How important in general: That people can understand how likely it is that they develop depression in the next year according to the AI [justice]** |  |  |  |  |
| Very important | 248 (67%) | 80 (61%) | 149 (69%) | 179 (63%) |
| Somewhat important | 92 (25%) | 42 (32%) | 53 (25%) | 80 (28%) |
| Not important | 12 (3.3%) | 3 (2.3%) | 7 (3.2%) | 8 (2.8%) |
| Don't know | 17 (4.6%) | 6 (4.6%) | 7 (3.2%) | 16 (5.7%) |
| **How important in general: That AI does not reduce people’s trust in their mental health care professionals [trust]** |  |  |  |  |
| Very important | 109 (30%) | 42 (32%) | 58 (27%) | 92 (33%) |
| Somewhat important | 180 (49%) | 64 (49%) | 113 (52%) | 131 (46%) |
| Not important | 54 (15%) | 18 (14%) | 34 (16%) | 38 (13%) |
| Don't know | 26 (7.0%) | 7 (5.3%) | 11 (5.1%) | 22 (7.8%) |
| **How important in general: That people are aware of how their personal data is being used for AI [privacy]** |  |  |  |  |
| Very important | 276 (75%) | 86 (66%) | 160 (74%) | 202 (71%) |
| Somewhat important | 66 (18%) | 26 (20%) | 36 (17%) | 55 (19%) |
| Not important | 14 (3.8%) | 16 (12%) | 14 (6.5%) | 16 (5.7%) |
| Don't know | 13 (3.5%) | 3 (2.3%) | 6 (2.8%) | 10 (3.5%) |
| **How important in general: That people can understand which of their individual risk factors for depression are used by the AI [transparency]** |  |  |  |  |
| Very important | 311 (84%) | 101 (77%) | 176 (81%) | 235 (83%) |
| Somewhat important | 44 (12%) | 24 (18%) | 31 (14%) | 37 (13%) |
| Not important | 5 (1.4%) | 5 (3.8%) | 4 (1.9%) | 6 (2.1%) |
| Don't know | 9 (2.4%) | 1 (0.8%) | 5 (2.3%) | 5 (1.8%) |
| **How important to you: That you are able to make up your own mind about your risk for depression based on AI output [autonomy]** |  |  |  |  |
| Very important | 236 (64%) | 89 (68%) | 135 (62%) | 189 (67%) |
| Somewhat important | 109 (30%) | 38 (29%) | 72 (33%) | 75 (27%) |
| Not important | 14 (3.8%) | 2 (1.5%) | 6 (2.8%) | 10 (3.5%) |
| Don't know | 10 (2.7%) | 2 (1.5%) | 3 (1.4%) | 9 (3.2%) |
| **How important to you: That AI will improve your depression/ depressive symptoms [beneficence]** |  |  |  |  |
| Very important | 218 (59%) | 84 (64%) | 124 (57%) | 178 (63%) |
| Somewhat important | 112 (30%) | 32 (24%) | 73 (34%) | 70 (25%) |
| Not important | 23 (6.2%) | 10 (7.6%) | 10 (4.6%) | 23 (8.1%) |
| Don't know | 16 (4.3%) | 5 (3.8%) | 9 (4.2%) | 12 (4.2%) |
| **How important to you: That AI will decrease the chance of negative outcomes [non-maleficence]** |  |  |  |  |
| Very important | 263 (71%) | 94 (72%) | 150 (69%) | 206 (73%) |
| Somewhat important | 90 (24%) | 33 (25%) | 55 (25%) | 68 (24%) |
| Not important | 11 (3.0%) | 2 (1.5%) | 9 (4.2%) | 4 (1.4%) |
| Don't know | 5 (1.4%) | 2 (1.5%) | 2 (0.9%) | 5 (1.8%) |
| **How important to you: That you can understand how likely it is that you develop depression within the next year according to the AI [justice]** |  |  |  |  |
| Very important | 257 (70%) | 86 (66%) | 153 (71%) | 190 (67%) |
| Somewhat important | 87 (24%) | 34 (26%) | 51 (24%) | 69 (24%) |
| Not important | 10 (2.7%) | 5 (3.8%) | 4 (1.9%) | 11 (3.9%) |
| Don't know | 15 (4.1%) | 6 (4.6%) | 8 (3.7%) | 13 (4.6%) |
| **How important to you: That using AI does not reduce your trust in your mental health care provider [trust]** |  |  |  |  |
| Very important | 137 (37%) | 49 (37%) | 81 (38%) | 105 (37%) |
| Somewhat important | 157 (43%) | 58 (44%) | 94 (44%) | 120 (42%) |
| Not important | 52 (14%) | 13 (9.9%) | 29 (13%) | 36 (13%) |
| Don't know | 23 (6.2%) | 11 (8.4%) | 12 (5.6%) | 22 (7.8%) |
| **How important to you: That you are aware of how your personal data is being used for AI [privacy]** |  |  |  |  |
| Very important | 265 (72%) | 83 (63%) | 155 (72%) | 192 (68%) |
| Somewhat important | 79 (21%) | 28 (21%) | 45 (21%) | 62 (22%) |
| Not important | 17 (4.6%) | 17 (13%) | 13 (6.0%) | 21 (7.4%) |
| Don't know | 8 (2.2%) | 3 (2.3%) | 3 (1.4%) | 8 (2.8%) |
| **How important to you: That you can understand which of your individual risk factors for depression are used by the AI [transparency]** |  |  |  |  |
| Very important | 297 (80%) | 105 (80%) | 167 (77%) | 234 (83%) |
| Somewhat important | 58 (16%) | 19 (15%) | 39 (18%) | 38 (13%) |
| Not important | 11 (3.0%) | 6 (4.6%) | 9 (4.2%) | 8 (2.8%) |
| Don't know | 3 (0.8%) | 1 (0.8%) | 1 (0.5%) | 3 (1.1%) |
| *Statistical significance represented by grey boxes. Statistical significance determined using Fisher's Exact Test for Count Data with simulated p-value (based on 2000 replicates).* | | | | |
